# Genomic Insights Into the Mechanism of Carbapenem Resistance Dissemination in Enterobacterales From a Tertiary Public Heath Setting in South Asia

**DOI:** 10.1093/cid/ciac287

**Published:** 2022-04-27

**Authors:** Refath Farzana, Lim S Jones, Md Anisur Rahman, Kirsty Sands, Andries J van Tonder, Edward Portal, Jose Munoz Criollo, Julian Parkhill, Martyn F Guest, W John Watkins, Monira Pervin, Ian Boostrom, Brekhna Hassan, Jordan Mathias, Md Abul Kalam, Timothy R Walsh

**Affiliations:** Department of Zoology, University of Oxford, Oxford, United Kingdom; Department of Medical Microbiology, Institute of Infection and Immunity, School of Medicine, Cardiff University, Cardiff, United Kingdom; Public Health Wales Microbiology, University Hospital of Wales, Cardiff, United Kingdom; Abdul Malek Ukil Medical College, Noakhali, Bangladesh; Department of Zoology, University of Oxford, Oxford, United Kingdom; Department of Veterinary Medicine, University of Cambridge, Cambridge, United Kingdom; Department of Medical Microbiology, Institute of Infection and Immunity, School of Medicine, Cardiff University, Cardiff, United Kingdom; Advanced Research Computing @Cardiff (ARCCA), Cardiff University, Cardiff, United Kingdom; Department of Veterinary Medicine, University of Cambridge, Cambridge, United Kingdom; Advanced Research Computing @Cardiff (ARCCA), Cardiff University, Cardiff, United Kingdom; Institute of Infection and Immunity, School of Medicine, Cardiff University, Cardiff, United Kingdom; Department of Virology, Dhaka Medical College, Dhaka, Bangladesh; Department of Medical Microbiology, Institute of Infection and Immunity, School of Medicine, Cardiff University, Cardiff, United Kingdom; Department of Medical Microbiology, Institute of Infection and Immunity, School of Medicine, Cardiff University, Cardiff, United Kingdom; Department of Medical Microbiology, Institute of Infection and Immunity, School of Medicine, Cardiff University, Cardiff, United Kingdom; Sheikh Hasina National Institute of Burn and Plastic Surgery, Dhaka, Bangladesh; Department of Zoology, University of Oxford, Oxford, United Kingdom

**Keywords:** carbapenem-resistant Enterobacterales, outbreak, plasmid-mediated resistance, Bangladesh, South Asia

## Abstract

**Summary:**

10.6% patients were CRE positive. Only 27% patients were prescribed at least 1 antibiotic to which infecting pathogen was susceptible. Burn and ICU admission and antibiotics exposures facilitate CRE acquisition. *Escherichia coli* ST167 was the dominant CRE clone.

**Background:**

Given the high prevalence of multidrug resistance (MDR) across South Asian (SA) hospitals, we documented the epidemiology of carbapenem-resistant Enterobacterales (CRE) infections at Dhaka Medical College Hospital between October 2016 and September 2017.

**Methods:**

We enrolled patients and collected epidemiology and outcome data. All Enterobacterales were characterized phenotypically and by whole-genome sequencing. Risk assessment for the patients with CRE was performed compared with patients with carbapenem-susceptible Enterobacterales (CSE).

**Results:**

10.6% of all 1831 patients with a clinical specimen collected had CRE. In-hospital 30-day mortality was significantly higher with CRE [50/180 (27.8%)] than CSE [42/312 (13.5%)] (*P* = .001); however, for bloodstream infections, this was nonsignificant. Of 643 Enterobacterales isolated, 210 were CRE; *bla*_NDM_ was present in 180 isolates, *bla*_OXA-232_ in 26, *bla*_OXA-181_ in 24, and *bla*_KPC-2_ in 5. Despite this, ceftriaxone was the most commonly prescribed empirical antibiotic and only 27% of patients were prescribed at least 1 antibiotic to which their infecting pathogen was susceptible. Significant risk factors for CRE isolation included burns unit and intensive care unit admission, and prior exposure to levofloxacin, amikacin, clindamycin, and meropenem. *Escherichia coli* ST167 was the dominant CRE clone. Clustering suggested clonal transmission of *Klebsiella pneumoniae* ST15 and the MDR hypervirulent clone, ST23. The major trajectories involved in horizontal gene transfer were IncFII and IncX3, IS*26*, and Tn*3*.

**Conclusions:**

This is the largest study from an SA public hospital combining outcome, microbiology, and genomics. The findings indicate the urgent implementation of targeted diagnostics, appropriate antibiotic use, and infection-control interventions in SA public institutions.

Carbapenem-resistant Enterobacterales (CRE) are one of the World Health Organization’s (WHO’s) listed critical priority pathogens [1]. The emergence and spread of CRE in a clinical setting drastically limit therapeutic options, increasing mortality and morbidity [2–4]. While the clonal expansion of multidrug-resistant (MDR) pathogens in nosocomial infections frequently occurs in settings with poor infection prevention and control policies, horizontal gene transfer plays a pivotal role in the spread of antimicrobial resistance (AMR) [3,5].

Several studies have documented the burden of carbapenem resistance in healthcare-associated infections in South Asia (SA) [6–8], and the genes *bla*_NDM_ and *bla*_OXA-181_ have been shown to be the predominant mechanisms of carbapenem resistance in the region [9–11]. However, there are significant data gaps and lack of AMR surveillance programs in SA (Supplementary Table 1) [6–8,12,13]. None of the previous studies in SA have described the impact, burden, and transmission dynamics of CRE by combining epidemiological, clinical, and genomic data (Supplementary Table 2).

This study was designed to better understand the molecular epidemiology of carbapenem resistance mechanisms at Dhaka Medical College Hospital (DMCH). The combination of whole-genome–based analysis with rigorous epidemiological data provides a powerful spatiotemporal assessment to explore the mechanisms and drivers of AMR in a Bangladeshi hospital setting.

## METHODS

### Study Design, Hospital Setting, Participants, and Sampling

We performed an observational cohort study at DMCH, the largest public hospital setting of Bangladesh, containing 2600 allocated beds, from October 2016 to September 2017. This study was approved by the Ethical Review Committee of DMCH (Supplementary Methods) [14].

**Figure 1.**
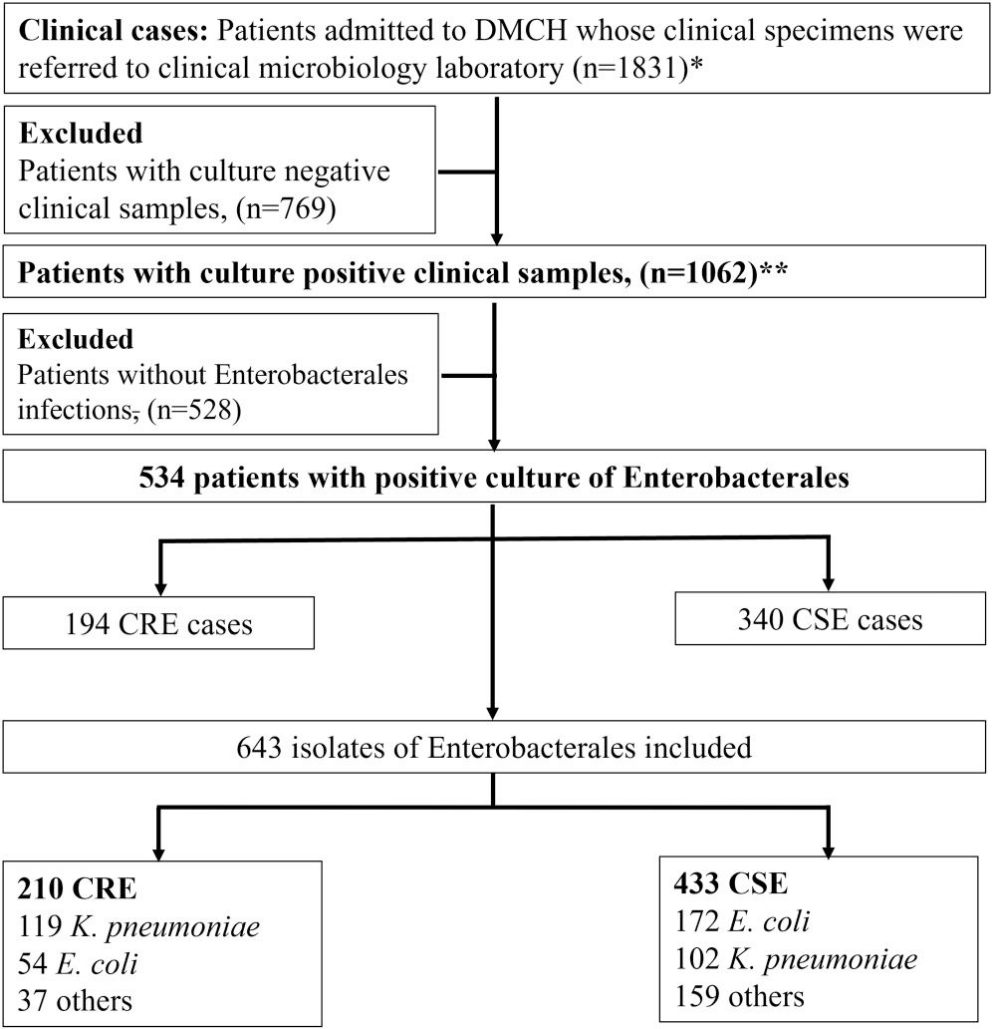
Flowchart diagram of participants included in this study. *Multiple clinical specimens were collected from 61 patients (blood and wound swab, n = 51; blood and urine, n = 3; blood and tracheal aspirate, n = 2; wound swab and urine, n = 2; blood and catheter tip, n = 1; urine and tracheal aspirates, n = 1; blood, urine, and catheter tip, n = 1).**Thirty-five patients had multiple culture-positive samples (blood and wound swab, n = 26; blood and urine, n = 2; blood and tracheal aspirate, n = 2; wound swab and urine, n = 2; blood and catheter tip, n = 1; urine and tracheal aspirates, n = 1; blood, urine, and catheter tip, n = 1). Abbreviations: CRE, carbapenem-resistant Enterobacterales; CSE, carbapenem-susceptible Enterobacterales; DMCH, Dhaka Medical College Hospital; *E. coli*, *Escherichia coli*; *K. pneumoniae*, *Klebsiella pneumoniae*.

Specimens referred to the DMCH microbiology laboratory for culture and sensitivity based on local physicians’ clinical judgment on suspected infections were included [15]. Participants’ demographic and clinical information was recorded (Supplementary Methods). All isolates recovered at the DMCH microbiology laboratory were transferred to Cardiff University and investigated further (n = 643) (Figure 1). Patients were enrolled for this study if at least 1 of their specimens was positive for Enterobacterales.

### Case Definition

Isolates were categorized as carbapenem-susceptible Enterobacterales (CSE) if sensitive to both imipenem and meropenem and CRE if resistant or increased exposure to either. For *Proteus*, *Providencia*, and *Morganella*, imipenem was excluded from the definitions because of intrinsic resistance [16]. The participants with at least 1 positive culture of CRE were considered as CRE cases. Any patient with a positive CSE culture was regarded as a CSE case.

### Phenotypic Characterization of Enterobacterales

Blood specimens were cultured using BacT/ALERT 3D (bioMerieux, USA) at DMCH. Isolates were subcultured onto chromogenic urinary tract infection agar (E&O Laboratories Ltd, Scotland, UK). The species were identified by matrix-assisted laser desorption/ionization time-of-flight mass spectrometry (Bruker Daltonics, Bremen, Germany). Minimum inhibitory concentrations (MICs) to clinically relevant antimicrobials were determined by agar dilution and interpreted according to European Committee on Antimicrobial Susceptibility Testing breakpoints (v10.0) [17,18].

### Whole-Genome Sequencing

All Enteribacterales isolated were sequenced on the Illumina MiSeq platform (Illumina, Inc, San Diego, CA, USA) and a set of *bla*_NDM-positive_ isolates by minION sequencing (Oxford Nanopore Technologies, Oxford, UK). Details of the bioinformatics analysis are described in the Supplementary Methods. Briefly, the Kmer database (v3.0.2) (available at the Center for Genomic Epidemiology [CGE]) was deployed for species identification, the Clermont phylotyping for *Escherichia coli* (v1.4.0), Kaptive (v0.7.3) for *Klebsiella pneumoniae* capsular typing. Comprehensive Antibiotic Resistance Database (CARD) and PlasmidFinder were deployed for antimicrobial resistance genes (ARGs) and plasmid replicon types with a cutoff of ≥95% coverage and ≥95% identity, respectively, using ABRicate (database for mass screening of contigs for antimicrobial resistance or virulence genes) (v0.9.7). Multilocus sequence type (MLST) was assigned based on 7 loci MLST databases in CGE (v2.0.0), where appropriate. Time-calibrated evolutionary analysis was performed using the BEAST package (v1.10.4) to estimate the date of the most recent common ancestor (MRCA).

### Scrutinizing “High-Risk” Clones for Carbapenem Resistance in Bangladeshi Hospital

The clonal relatedness of isolates was assessed using core-genome alignment following clustering for the presence and absence of genes, MLST profiling, and pairwise single nucleotide polymorphism (SNP) distances. A cutoff of ≤10 SNPs combining with epidemiological information was used to define possible clonal transmission clusters using the R library iGRAPH [19]. Clusters were removed if they did not contain any carbapenemase producer.

### Statistical Analysis

Binary logistic regression was used to analyze the association of CRE with categorical variables of interest. The *P* values were adjusted by the Benjamini-Hochberg procedure when repetitive variable types were tested. A Cox proportional hazards model was used to compare all-cause in-hospital 30-day mortality between CRE and CSE cases. Patients discharged alive or with an in-hospital mortality over 30 days were used as the competing variable for outcome analysis. Statistical analyses were conducted using IBM SPSS (v26; IBM Corporation) and Tableau (v2020.4).

## RESULTS

### Prevalence of Carbapenem Resistance in Clinical Enterobacterales

A total of 1893 clinical specimens from 1831 patients were included. Fifty-eight percent (1098/1893) of the specimens were culture positive and 1583 isolates were recovered (Supplementary Tables 3–5). The proportion of Enterobacterales isolated was 33.9% (643/1893) and CRE comprised 11.1% (210/1893). The prevalence of CRE cases was 10.6% (194/1831) (Figure 1).

Of 643 Enterobacterales, 210 were CRE (12.6% were recovered from wound swabs, 7.8% from urine, and 7.5% from blood) and 433 were CSE (28.5% from wound swabs, 24.1% from urine, and 10.1% from blood) (Figure 1) (Supplementary Table 4).

The predominant species were *E. coli* (226/1583, 14.3%), *K. pneumoniae* (221/1583, 14%), *Proteus mirabilis* (64/1583, 4%), and *Enterobacter cloacae* complex (39/1583, 2.5%) (Supplementary Table 5).

### Antimicrobial Resistance Profile

**Figure 2.**
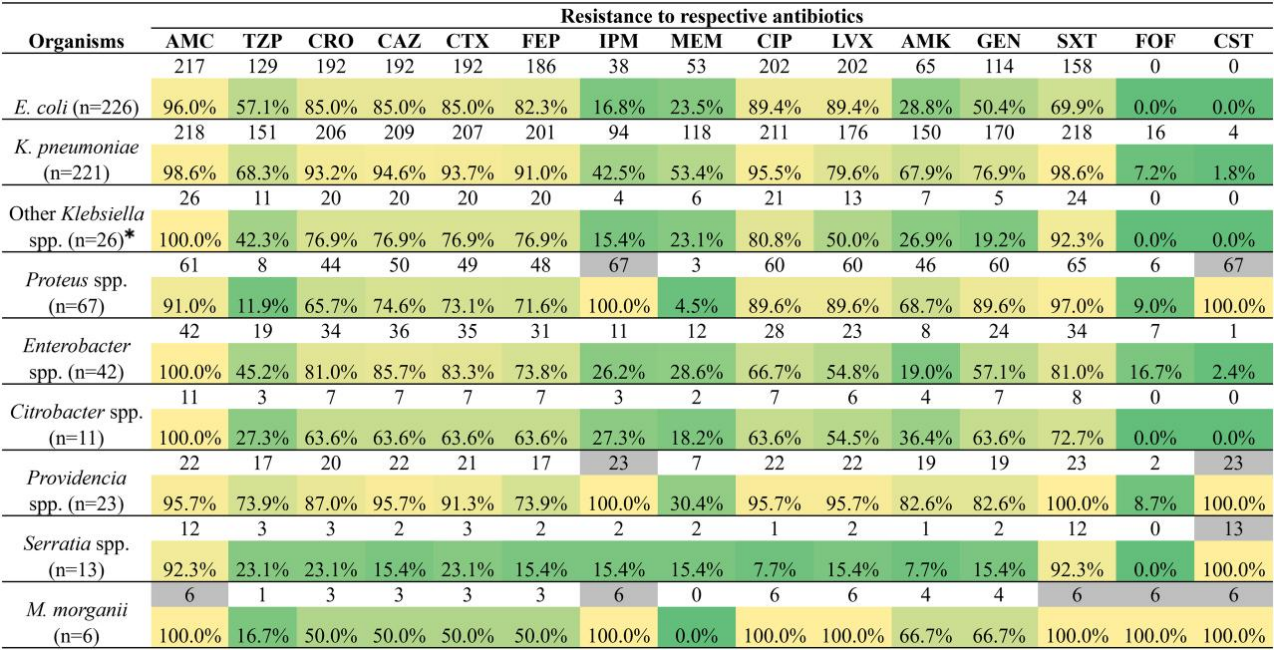
Antimicrobial susceptibility patterns of different species of Enterobacterales. Data on *Salmonella* spp. (n = 5), *P. anthophila* (n = 1), *L. adecarboxylata* (n = 1), and *E. hermannii* (n = 1) are not included in this table. *Klebsiella species other than *K. pneumoniae*. The upper cells corresponding to each species represent the frequency of resistance and the lower cells represent percentage. The heatmap indicates higher (yellow) to lower (green) percentages of resistance. Cells are highlighted in gray if the respective organism is intrinsically resistant to the pertinent antibiotic. Abbreviations: AMC, amoxicillin-clavulanic acid; AMK, amikacin; CAZ, ceftazidime; CIP, ciprofloxacin; CRO, ceftriaxone; CST, colistin; CTX, cefotaxime; *E. coli*, *Escherichia coli*; FEP, cefepime; FOF, fosfomycin; GEN, gentamicin; IPM, imipenem; *K. pneumoniae*, *Klebsiella pneumoniae*; LVX, levofloxacin; *M. morganii*, *Morganelli morganii*; MEM, meropenem; SXT, sulfamethoxazole-trimethoprim; TZP, piperacillin-tazobactam.

We found a high frequency of resistance in Enterobacterales against both β-lactam and non–β-lactam antibiotics except for colistin and fosfomycin. Excluding intrinsically resistant species, 4.9% (31/636) was resistant to fosfomycin and 0.9% (5/534) to colistin (Figure 2). CRE exhibited significantly higher resistance rates to ciprofloxacin, levofloxacin, amikacin, gentamicin, and sulfamethoxazole-trimethoprim than CSE (*P* < .0001) (Table 1).

**Table 1.** Phenotypic and Genomic Resistance Profile of Carbapenem-Resistant Enterobacterales

Antimicrobial Groups	Phenotypic Resistance of Enterobacterales, n (%) (n = 643)	Associations Between Phenotypic Resistance and CRE,^a^ n (%)			Carbapenemase Alleles Identified in This Study	ARGs Significantly Associated With Carbapenem-Resistant Genes^b^	
		CRE (n = 210)	CSE (n = 433)	*P*		*bla*_NDM-5_	*bla*_NDM-1_
AMC	617 (96)	210 (100)	407 (93.9)		…	*bla*_TEM-1_	…
TZP	342 (53.2)	208 (99)	134 (30.9)		*…*	*bla*_OXA-1_	*bla*_OXA-1_, *bla*_OXA-9_
CRO	531 (82.6)	210 (100)	321 (74.1)		…	*bla*_CMY-59_, *bla*_CTX-M-15_, *bla*_VEB-5_	…
CAZ	543 (84.4)	210 (100)	333 (76.9)		…	…	…
CTX	539 (83.8)	210 (100)	329 (76)		…	…	…
FEP	517 (80.4)	210 (100)	307 (70.9)		…	…	…
IPM	248 (38.6)	166 (79)	82 (18.9)		*bla*_NDM-5_, *bla*_NDM-5_, *bla*_NDM-7_, *bla*_NDM-4_, *bla*_OXA-181_, *bla*_OXA-232_	…	…
MEM	203 (31.6)	203 (96.7)	0 (0)		…	…	…
CIP	559 (86.9)	206 (98.1)	353 (81.5)	<.0001	…	*qnrS1*	*qnrA1*, *qnrB17*, *qnrD1*
LVX	511 (79.5)	191 (91)	320 (73.9)	<.0001	…	…	…
AMK	305 (47.4)	190 (90.5)	115 (26.5)	<.0001	…	*aadA2*, *APH(3*″*)-Ib*, *APH(3*″*)-Ia*, *APH(6)-Id*, *armA*, *rmtB*	*AAC(2*′*)-Ia, aadA2*, *APH(3’)-Ia*, *APH(3’)-VI, armA, rmtF*
GEN	407 (63.3)	197 (93.8)	210 (48.5)	<.0001	…	…	…
SXT	550 (85.5)	200 (95.2)	350 (80.8)	<.0001	…	*dfrA12*, *sul1*, *sul2*	*dfrA14, sul1*
FOF	38 (5.9)	18/210 (8.6)^c^	13/426 (3.1)^c^	.002	…	…	…
CST	97 (15.1)	1/194 (0.5)^d^	4/340 (1.2)^d^	.446	…	…	…

Abbreviations: AMC, amoxicillin-clavulanic acid; AMK, amikacin; ARG, antimicrobial resistance gene; CAZ, ceftazidime; CIP, ciprofloxacin; CRE, carbapenem-resistant Enterobacterales; CRO, ceftriaxone; CSE, carbapenem-sensitive Enterobacterales; CST, colistin; CTX, cefotaxime; FEP, cefepime; FOF, fosfomycin; GEN, gentamicin; IPM, imipenem; LVX, levofloxacin; MEM, meropenem; SXT, sulfamethoxazole-trimethoprim; TZP, piperacillin-tazobactam;.

To compare the differences in resistance to non–β-lactams between CRE and CSE, *P* values were calculated.

As *bla*_NDM-5_ and *bla*_NDM-1_ are the major carbapenemases in this study, ARGs significantly associated with *bla*_NDM-5_ and *bla*_NDM-1_ compared with *bla*_NDM-5_-negative and *bla*_NDM-1_-negative Enterobacterales were included in this table. Details about the analysis are described in Supplementary Tables 5 and 6.

*Morganella morganii* (n = 6) and *Leclercia adecarboxylata* (n = 1) were excluded from the analysis as the species are intrinsically resistant to fosfomycin.

*Proteus* spp. (n = 67), *Providencia* spp. (n = 23), *Serratia marcescens* (n = 13), and *M. morganii* (n = 6) were excluded from the analysis as the species are intrinsically resistant to colistin.

**Figure 3.**
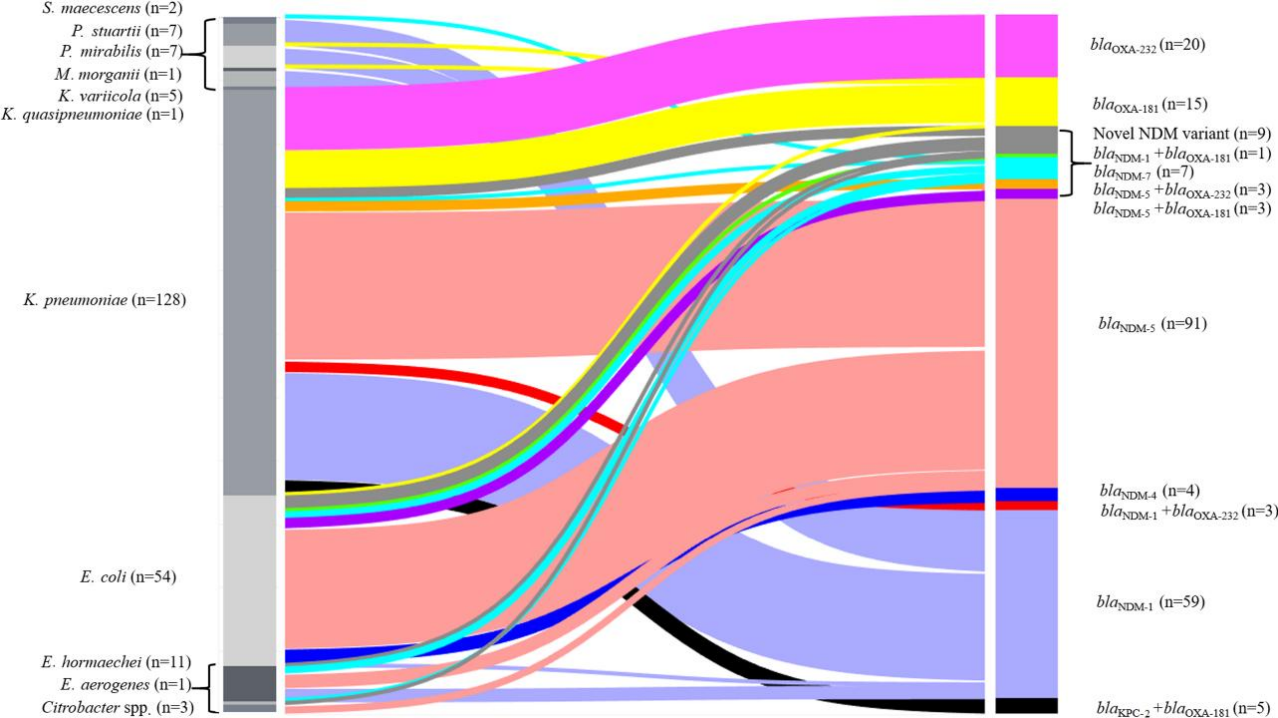
Sankey diagram representing the distribution of carbapenemase alleles among different species of Enterobacterales. Abbreviations: *E.*, *Escherichia*; *K.*, *Klebsiella*; KPC, *Klebsiella pneumoniae* carbapenemase; *M.*, *Morganella*; NDM, New Delhi metallo-beta-lactamase; OXA, oxacillinases; *P.*, *Providencia*; *S.*, *Serratia*.

Carbapenemases identified were *bla*_NDM_ (180/643, 28%) (predominantly *bla*_NDM-5_ [97/643, 15.1]), *bla*_OXA-232_ (26/643, 4%), *bla*_OXA-181_ (24/643, 3.7%), and *bla*_KPC-2_ (5/643, 0.8%) (Figure 3). A small number of phenotypically carbapenem susceptible isolates were positive for *bla*_OXA-232_ (n = 9) and *bla*_OXA-181_ (n = 1) (Supplementary Table 9).

The clinically important resistance genes—*aadA2*, *APH*, *armA*, *bla*_CTX-M-15_, and *bla*_TEM-1_—were associated with *bla*_NDM_-positive isolates (*P* < .05) (Table 1).

### Risk and Outcome Analysis

Of 534 clinical cases, 194 (36.3%) were CRE cases and 340 (63.7%) were CSE cases. Our data indicated that 94.1% (503/534) of the patients with Enterobacterales infections were treated with empirical antibiotics on admission to DMCH. Ceftriaxone was the most commonly prescribed antimicrobial (328/534, 61.4%) among the participants, followed by metronidazole (159/534, 29.8%), ciprofloxacin (123/534, 23%), and amikacin (103/534, 19.3%). Carbapenem usage was 13.1% (meropenem) and 1.5% (imipenem).

Being part of the 6- to 25-year age group (*P* = .041), being female (*P* = .029), burns unit and (*P* < .0001), intensive care unit (ICU) admission (*P* = .001), and exposure to certain antibiotics (levofloxacin [*P* < .0001], amikacin [*P* = .008], clindamycin [*P* = .008], and meropenem [*P* = .044]) were associated with increased risk of CRE infections. Statistical associations remained unchanged in the adjusted models (Table 2).

**Table 2.** Descriptive Statistics for Risk Assessment of Carbapenem-Resistant Enterobacterales Clinical Cases Compared With Carbapenem-Susceptible Enterobacterales Cases

Attributes		CRE (n = 194)	CSE (n = 340)	Unadjusted Logistic Regression			Adjusted for Patients Admitted to Burns Unit			Adjusted for Patients Infected With CSE With Carbapenemases Producers^a^		
				*P*	OR	95% CI	*P*	OR	95% CI	*P*	OR	95% CI
Age, y^b^	0 to 5	27 (13.9)	49 (14.4)	.875	.960	.578–1.594	.976	.992	.592–1.662	.918	.973	.584–1.622
	6 to 25	67 (34.5)	89 (26.2)	.041	1.488	1.015–2.180	.231	1.274	.857–1.893	.033	1.521	1.034–2.235
	26 to 50	66 (34)	130 (38.2)	.331	.833	.576–1.204	.514	.882	.605–1.285	.307	.824	.569–1.194
	>50	34 (17.5)	73 (21.5)	.273	.777	.495–1.221	.542	.866	.546–1.374	.238	.761	.484–1.198
Gender^c^	Female	81 (41.8)	110 (32.4)	.029	.667	.463–.961	.052	.692	.477–1.003	.027	.660	.457–.953
	Male	113 (58.2)	230 (67.6)									
SE group^b^	BPL	84 (43.3)	161 (47.4)	.366	1.178	.826–1.680	.412	1.164	.810–1.671	.415	1.160	.812–1.657
	Poor	74 (38.1)	134 (39.4)	.773	1.055	.734–1.515	.671	1.083	.749–1.568	.725	1.067	.742–1.536
	LM	34 (17.5)	40 (11.8)	.064	.627	.382–1.030	.062	.618	.372–1.024	.070	.631	.383–1.038
	UM^d^	2 (1)	4 (1.2)		…	…		…	…		…	…
	UH^d^	0 (0)	1 (0.3)		…	…		…	…		…	…
Admitting wards^b^	Burns	74 (38.1)	69 (20.3)	<.0001	.413	.279–.611		…	…	<.0001	.368	.246–.549
	Surgery	14 (7.2)	73 (21.5)	<.0001	3.515	1.925–6.420		…	…	<.0001	3.588	1.963–6.559
	Urology	23 (11.9)	64 (18.8)	.036	1.724	1.032–2.880		…	…	.027	1.789	1.070–2.990
	ICU	33 (17)	27 (7.9)	.001	.421	.245–.724		…	…	.003	.435	.253–.749
	Other wards	50 (25.8)	107 (31.5)	.165	1.323	.891–1.963		…	…	.126	1.363	.917–2.025
Comorbidity (DM)^c^	Yes	15 (7.7)	58 (17.1)	.003	2.454	1.350–4.463	.015	2.127	1.160–3.901	.003	2.492	1.369–4.536
	No	179 (92.3)	282 (82.9)									
Comorbidity (malignancy)^c^	Yes	7 (3.6)	15 (4.4)	.653	1.233	.494–3.078	.935	1.039	.411–2.625	.606	1.272	.509–3.177
	No	187 (96.4)	324 (95.6)									
Antibiotics exposure during hospital stay before sampling^b,e^	Ceftriaxone	128 (66)	200 (58.8)	.102	.737	.510–1.063	.625	.908	.617–1.336	.058	.700	.484–1.012
	Metronidazole	49 (25.3)	110 (32.4)	.085	1.415	.953–2.102	.889	1.031	.671–1.584	.072	1.440	.968–2.142
	Ciprofloxacin	27 (13.9)	96 (28.2)	<.0001	2.434	1.521–3.894	.007	1.958	1.202–3.191	<.0001	2.500	1.561–4.006
	Amikacin	49 (25.3)	54 (15.9)	.008	.559	.362–.863	.021	.592	.380–.923	.006	.541	.348–.840
	Meropenem	33 (17)	37 (10.9)	.044	.596	.359–.989	.017	.532	.317–.894	.061	.616	.371–1.023
	Flucloxacillin	27 (13.9)	70 (20.6)	.054	1.604	.988–2.602	.064	1.594	.974–2.608	.080	1.546	.949–2.519
	Levofloxacin	45 (23.2)	35 (10.3)	<.0001	.380	.234–.616	.116	.615	.336–1.128	<.0001	.343	.209–.565
	Clindamycin	28 (14.4)	25 (7.4)	.008	.471	.266–.833	.075	.585	.324–1.056	.009	.465	.261–.828
Number of antibiotics prescribed^c,f^	Monotherapy	19 (10.3)	50 (15.7)	.093	.620	.353–1.087	.315	.745	.419–1.324	.072	.596	.340–1.048
	More than 1 drug	165 (89.7)	269 (84.3)									
Hospital stay before sampling**^c^**	≤7 days	68 (35.1)	136 (40)	.258	.810	.561–1.167	.590	.902	.619–1.313	.184	.780	.540–1.126
	>7 days	126 (64.9)	204 (60)									

Values in parentheses indicate column percentage. Binary logistic regression was performed to assess risks, and to calculate OR and 95% CI. Abbreviations: BPL, below the poverty level; CI, confidence interval; CRE, carbapenem-resistant Enterobacterales; CSE, carbapenem-sensitive Enterobacterales; DM, diabetes mellitus; ICU, intensive care unit; LM, lower middle; OR, odds ratio; SE, socioeconomic; UH, upper high; UM, upper middle.

We found the presence of *bla*_OXA-232_ (n = 9) and *bla*_OXA-181_ (n = 1) in phenotypically carbapenem-susceptible isolates.

The attributes having >2 possible values; each value was compared with all the others combined: eg, for age group 0 to 5 years, a binary variable 0 to 5 against all other age groups was used, for 6 to 25, the binary age variable was 6 to 25 versus all other age bands.

Attributes with 2 categories such as sex, number of antibiotics prescribed, hospital stay before sampling, and comorbidity; the logistic regressions had 1 of the categories as the reference value.

Statistical analysis was not performed due to low frequency of cases.

Eight common antibiotics prescribed at the Dhaka Medical College Hospital are included in this descriptive analysis.

Patients without any antibiotic (n = 31) were excluded from the analysis.

Excluding patients discharged against medical advice, all-cause in-hospital 30-day mortality was significantly associated with CRE cases, occurring in 50 of 180 (27.8%) CRE and 42 of 312 (13.5%) CSE cases (*P* = .001). Significant associations were also observed after adjusting for the confounders (Table 3). No significant association of mortality was observed with CRE for the cohort of patients with Enterobacterales bloodstream infections (BSIs) (Table 4).

**Table 3.** Cox Proportional Hazards Models to Analyze the Impact of Carbapenem Resistance and Mortality Among the Patients With Positive Culture of Enterobacterales

Cohort	All-Cause In-Hospital 30-Day Mortality	Discharged Alive/In-Hospital Mortality After 30 Days	*P* ^a^	SHR^a^	95% CI^a^
Patients with positive culture of Enterobacterales (n = 492)					
ȃCRE (n = 180)	50 (27.8)	130 (72.2)	.001	0.491	.325–.741
ȃCSE (n = 312)	42 (13.5)	270 (86.5)			
Model 1: Adjusted by age and gender			.001	0.510	.337–.771
Model 2: Model 1 + adjusted by admission to burn unit^b^			.007	0.561	.367–.855
Model 3: Model 1 + adjusted by admission to ICU^b^			.051	0.654	.428–1.001
Model 4: Model 1 + adjusted by exposure to amikacin^b^			.004	0.537	.354–.816
Model 5: Model 1 + adjusted by exposure to meropenem^b^			.003	0.535	.353–.812
Model 6: Model 1 + adjusted by exposure to levofloxacin^b^			.008	0.562	.368–.859
Model 7: Model 1 + adjusted by exposure to clindamycin^b^			.003	0.529	.349–.803

Values in parenthesis indicate row percentage. Patients who were discharged against medical advice (n = 40) and outlier cases (n = 2) (hospital stay >100 days from “time from infection” to outcome) were excluded from the outcome analysis. Abbreviations: CI, confidence interval; CRE, carbapenem-resistant Enterobacterales; CSE, carbapenem-sensitive Enterobacterales; SHR, subdistribution hazard ratio.

A Cox proportional hazards model was fitted with time points “time from Enterobacterales isolation to outcome” as “time-to-event” and “time from admission to Enterobacterales isolation” as covariates.

Confounders such as age, gender, admission to burn unit and ICU, and exposure to amikacin, meropenem, levofloxacin, and clindamycin were adjusted to understand the changes in *P* values (level of statistical significance) in the adjusted model.

**Table 4.** Cox Proportional Hazards Models to Analyze the Impact of Carbapenem Resistance and Mortality Among the Patients with Positive Blood Culture of Enterobacterales

Cohort	All-Cause In-Hospital 30-Day Mortality	Discharged Alive/In-Hospital Mortality After 30 Days	*P*	SHR	95% CI
Patients with positive blood culture of Enterobacterales (n = 83)					
ȃCRE (n = 38)	19 (50)	19 (50)	.571	0.834	.445–1.562
ȃCSE (n = 45)	22 (48.9)	23 (51.1)			

Values in parenthesis indicate row percentage. Patients who were discharged against medical advice (n = 40) and outlier cases (n = 2) (hospital stay >100 days from “time from infection” to outcome) were excluded from the outcome analysis. A Cox proportional hazards model was fitted with time points “time from Enterobacterales isolation to outcome” as “time-to-event” and “time from admission to Enterobacterales isolation” as covariates. Abbreviations: CI, confidence interval; CRE, carbapenem-resistant Enterobacterales; CSE, carbapenem-sensitive Enterobacterales; SHR, subdistribution hazard ratio.

Based on the available data, only 27% (144/534) of the patients were prescribed at least 1 antibiotic to which their infecting pathogens were susceptible (Supplementary Table 10); however, data were not available regarding dosage, duration, and indication of antibiotics therapy.

### “High-Risk” Clones for Carbapenem Resistance

#### Escherichia coli

**Figure 4.**
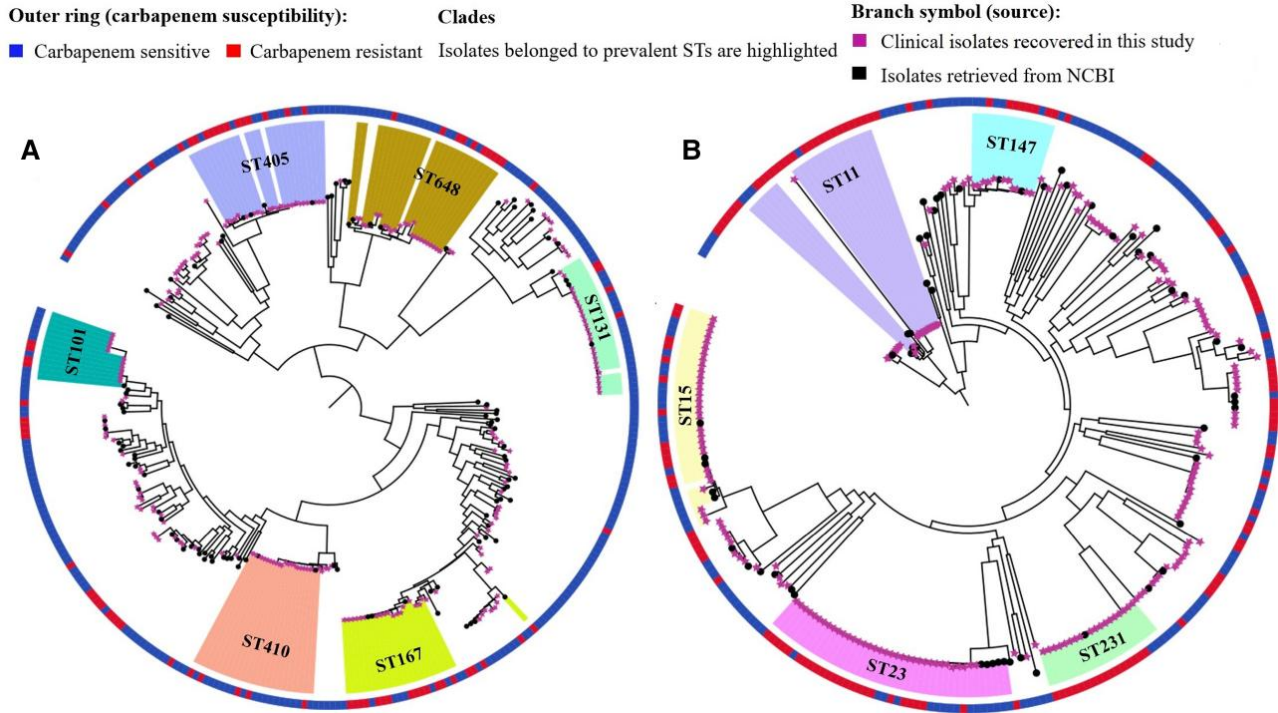
ML tree generated from core-genome analysis of *Escherichia coli* and *Klebsiella pneumoniae* isolated in this study. (*A*) ML tree generated from core-genome analysis of *E. coli*. (*B*) ML tree generated from core-genome analysis of *K. pneumoniae*. Core-genome alignment was performed using roary (a tool that rapidly builds large-scale pan genomes, identifying the core and accessory genes) (v3.12.0). The ML trees from the core genome were built with RAxML-ng (v0.9.0.git-mpi) using a GTR evolutionary model and gamma correction with bootstrapping. Isolates retrieved from the NCBI for the phylogenetic analysis in this figure are stated in Supplementary Table 17. Abbreviations: GTR, generalised time reversible; ML, maximum likelihood; NCBI, National Center for Biotechnology Information; ST, sequence type.

The prevalent sequence types (STs) among clinical *E. coli* included ST131 (23/226, 10.2%), ST405 (21/226, 9.3%), ST648 (21/226, 9.3%), ST410 (21/226, 9.3%), and ST167 (18/226, 8%) (Figure 4*A* ). ST167 was significantly associated with carbapenem resistance (*P* = .004). The majority of *E. coli* belonged to phylogroup A (53/226, 23.5%) and D (49/226, 21.7%) followed by others (Supplementary Tables 11 and 12).

**Figure 5.**
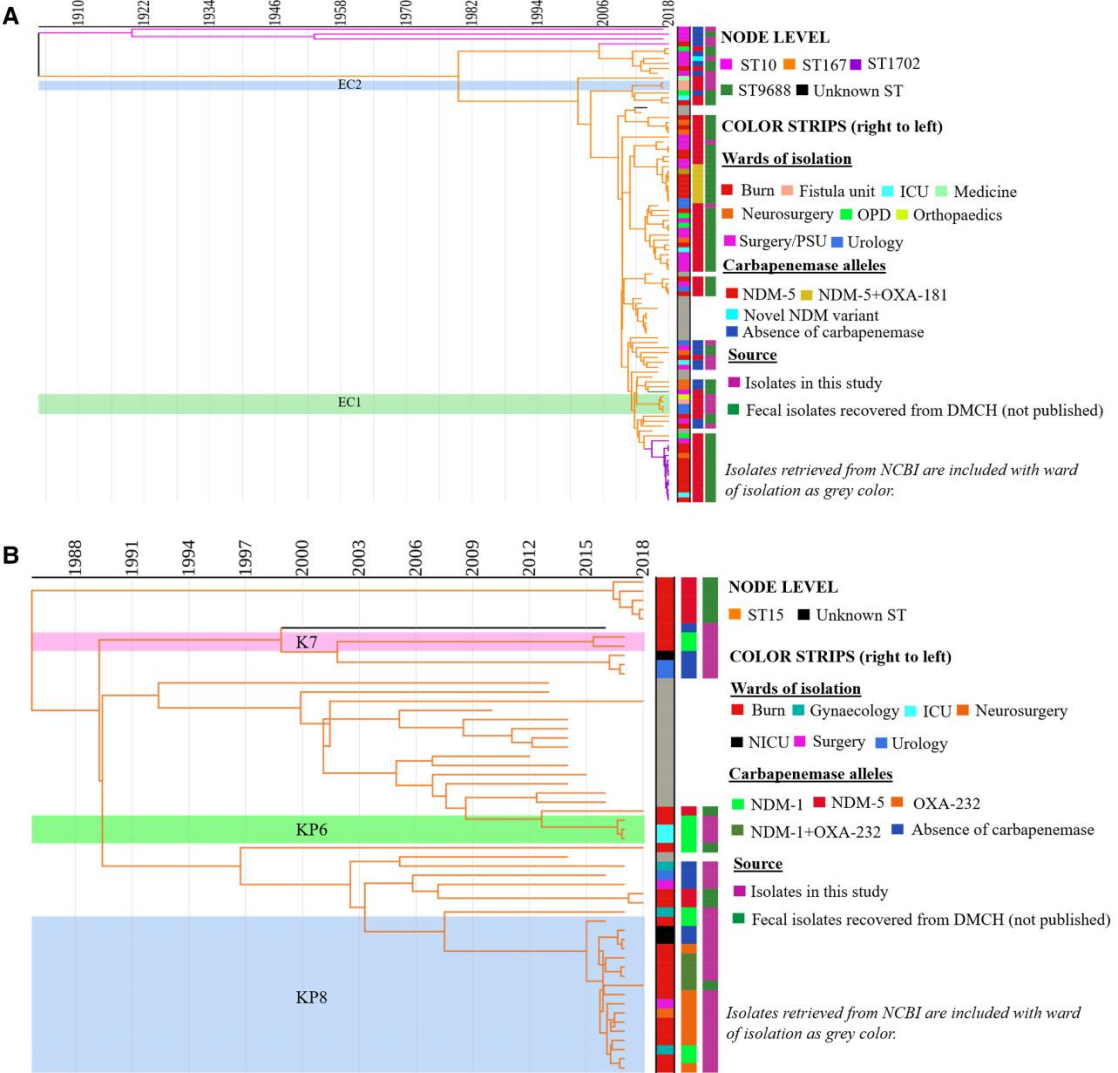
Time-calibrated phylogenetic tree generated from *Escherichia coli* and *Klebsiella pneumoniae* genomes. (*A*) Phylogenetic tree generated from *E. coli* genomes belonging to ST167. The total number of isolates in this analysis was 97. Closely related isolates from other STs (ST10, ST1702, and novel allele) identified by core-genome phylogeny and pairwise SNPs count (if isolates differed by ≤100 SNPs from any isolate of ST167) were included in this analysis. (*B*) Phylogenetic tree generated from *K. pneumoniae* genomes belonging to ST15. The total number of isolates in this analysis was 54. Closely related isolates from a novel allele identified by core-genome phylogeny and pairwise SNP count (if isolates differed by ≤100 SNPs from any isolate of ST15) were included in this analysis. Putative transmission clades (0–10 SNP differences) are highlighted in green. MRCA and clock rate are stated in Supplementary Table 1^3^. Isolates retrieved from the NCBI for the phylogenetic analysis in this figure are shown in Supplementary Table 17. Abbreviations: DMCH, Dhaka Medical College Hospital; ICU, intensive care unit; NCBI, National Center for Biotechnology Information; NDM, New Delhi metallo-beta-lactamase; NICU, neonatal intensive care unit; OPD, outpatient department; PSU, pediatric surgery; SNP, single nucleotide polymorphism; ST, sequence type.

Bayesian phylogenetic analysis suggests that the populations of major *E. coli* clones had the MRCA between 1978 and 2007, including isolates from outside the hospital (taken from National Center for Biotechnology Information [NCBI]). Dates of MRCAs for hospital-only subclades ranged from 2004–2015 for ST167, 2008–2017 for ST448, 2010–2013 for ST8346, 1990–1999 for ST405, and 2006–2013 for ST648 (Figure 5*A* , Supplementary Figures 2–5). The average median substitution rate of *E. coli* was 3.11 SNPs per genome/year (Supplementary Table 1^3^).

#### Klebsiella pneumoniae

Predominant *K. pneumoniae* STs included ST23 (35/221, 15.8%) and ST15 (29/221, 13.1%) (Figure 4*B* ). All isolates belonging to ST16 and ST515 were resistant to carbapenems. The prevalent clinical clone, ST23, having KL1 capsular type did not have a significant association with carbapenem resistance; however, 68.6% (24/35) of isolates of ST23 were found to be carbapenem resistant (odds ratio: .478; 95% confidence interval: .222–1.033) (Supplementary Tables 14 and 15).

The dates for the MRCAs of *K. pneumoniae* including NCBI isolates were between 1932 and 1980. The DMCH’s subclades emerged between 1998 and 2016 for ST15 and 2014 and 2017 for ST16 (Figure 5*B* , Supplementary Figure 5). The average median substitution rate was 2.22 SNPs per genome/year (Supplementary Table 1^3^).

### Clonal Transmission of Carbapenem Resistance

**Figure 6.**
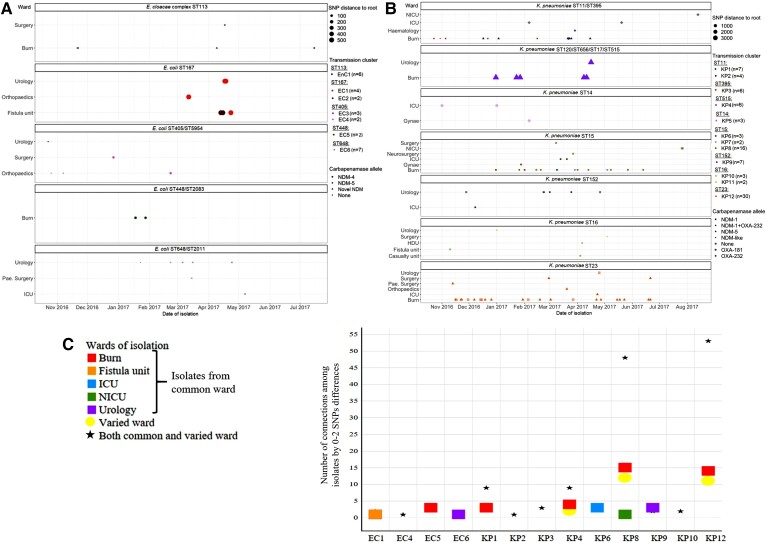
Spatiotemporal assessment to investigate putative clonal transmission of carbapenem resistance. (*A*) Putative transmission clusters of *Escherichia coli* at a ≤10-SNP threshold between the isolates in the respective clusters. (*B*) Putative transmission clusters of *Klebsiella pneumoniae* at a ≤10-SNP threshold between the isolates in the respective clusters. The ancestral sequence at each node including the root was inferred using pyjar and the pairwise SNP distance between the roots and each isolate was calculated using pairsnp (v0.0.7). Pairwise SNPs between isolates were generated using pairsnp (v0.0.7). (*C*) Diagram representing number of linkages among the isolates in the clusters by the 0- to 2-SNP threshold, aligning with epidemiological data. Isolates differed by 0 to 2 SNPs without overlapping of pertinent patients’ hospital stay are represented as a common group in the figure using Tableau (v2020.4). Abbreviations: HDU, high dependency unit; ICU, intensive care unit; NDM, New Delhi metallo-beta-lactamase; NICU, neonatal intensive care unit; SNP, single nucleotide polymorphism; ST, sequence type.

We identified 5 clusters of *E. coli* (EC1, EC2, and EC4 to EC6), 12 of *K. pneumoniae* (KP1 to KP12), and 1 of *E. cloacae* complex (EnC1) using a 10-SNP threshold between isolates with common carbapenemase alleles and none of cluster-contained isolates outside DMCH included in our analysis (Figure 6). We found a linkage between isolates in the clusters at a ≤2-SNP threshold using overlapping of patients’ hospital stays and common wards of isolation. These connections were observed in the burns unit, fistula unit, ICU, neonatal ICU, and urology. The largest cluster was KP12 (ST23) followed by KP8 (ST15) (Figure 6*C* ). The dated phylogenetic tree of major clones revealed that the putative transmission clusters were predicted to have been introduced into DMCH between 2013 and 2016 (Supplementary Table 1^3^).

### Investigating Transmission of Carbapenem Resistance Due to Horizontal Transfer of Plasmids

A total of 125 isolates were characterized by hybrid assembly of short-reads and long-reads sequence data, yielding complete, circular plasmids harboring *bla*_NDM-5_ (n = 74), *bla*_NDM-1_ (n = 37), *bla*_NDM-7_ (n = 6), *bla*_NDM-4_ (n = 3), *bla*_OXA-232_ (n = 7), and *bla*_OXA-181_ (n = 4). The major incompatibility (Inc) types in association with different carbapenemase alleles were the following: IncFII (n = 41), IncX3 (n = 9), and IncFIA (n = 9) in association with *bla*_NDM-5_; IncC (n = 10) and IncFIB and IncHI1B (n = 6) in association with *bla*_NDM-1_; IncX3 (n = 6) in association with *bla*_NDM-7_; and ColKP3 (n = 6) in association with *bla*_OXA-232_ (Table 5). Plasmids of different Inc types harboring *bla*_NDM_ typically carried multiple ARGs except for IncX3 (Supplementary Figure 6). The clinically important ARGs in associations with *bla*_NDM_-positive plasmids were as follows: *bla*_TEM-1_ (70/120, 58.3%) and *bla*_CTX-M-15_ (21/120, 17.5%) for β-lactams; *aadA* (73/120, 60.8%), *rmt* (72/120, 60%), *armA* (19, 15.8%), *AAC(6*′*)-Ib* (38/120, 31.7%), *APH(3*″*)* (20/120, 16.7%), *ANT(3*″*)-IIa* (5/120, 4.2%), *AAC(3)* (3/120, 2.5%), and *APH(6)-Id* (3/120, 2.5%) for aminoglycosides; *sul* (93/120, 77.5%) for sulphonamides; *dfrA* (76/120, 63.3%) for trimethoprim; *qnrB* (8/120, 6.7%); and *qnrS1* (5/120, 4.2%) for quinolones.

**Table 5.** Stratification of Plasmids Based on Resistance Patterns, Inc Types, and Plasmid Size

Plasmid Inc Type	Size of Plasmid	Similarity of Plasmids in a Group at the Nucleotide Level	Group Designation for This Study	Bacterial Host (n^a^)
NDM-5–positive plasmids				
ȃIncFII	∼71 kb	…	FII_N5_1	*Escherichia coli*: ST410 (1)
	∼80 to ∼87 kb	Coverage: 87% to 100%; identity: ≥99%	**FII_N5_2**	*E. coli*: ST101 (2), ST405 (2), ST2083 (1), ST617 (1) *Klebsiella pneumoniae*: ST11 (1)
	∼91 to ∼99 kb	Coverage: 83% to 100%; identity: ≥99%	**FII_N5_3**	*K. pneumoniae*: ST23 (11), ST515 (3), ST147 (2), ST48 (2), ST11 (1), ST16 (1), ST490 (1) *E. coli*: ST5954 (1), ST10820 (1), ST2659 (1), ST405 (1), ST448 (1), ST8346 (1) *E. cloacae* (1) *Citrobacter rodentium* (2)
	∼127 kb	…	FII_N5_4	*E. coli* ST648 (1)
	∼238 kb	…	FII_N5_5	*K. pneumoniae* ST23 (1)
ȃIncX3	∼45 to ∼49 kb	Coverage: 100%; identity: ≥99%	X3_N5_1	*E. coli*: ST448 (3), ST167 (2) *E. cloacae* ST113 (3) *K. pneumoniae* ST16 (1)
ȃIncFIA	∼106 kb	…	FIA_N5_1	*E. coli* ST648 (1)
	∼118 kb	…	FIA_N5_2	*E. coli* ST167 (1)
	∼127 to ∼128 kb	Coverage: 100%; identity: ≥99%	FIA_N5_3	*E. coli* ST167 (4)
	∼131 kb	…	FIA_N5_4	*E. coli* ST131 (1)
	∼152 kb	…	FIA_N5_5	*E. coli* ST405 (1)
	∼159 kb	…	FIA_N5_6	*E. coli* ST167 (1)
ȃIncFIB(pQil)	∼134 kb	Coverage: 99% to 100%; identity: ≥99%	FIB(pQil)_N5_1	*K. pneumoniae* ST231 (3)
	∼163 kb	…	FIB(pQil)_N5_2	*K. pneumoniae* ST16 (1)
ȃIncR	∼112 kb	…	R_N5_1	*E. coli* ST410 (1)
	∼143 kb	Coverage: 99% to 100%; identity: ≥99%	R_N5_2	*K. pneumoniae* ST23 (3)
ȃIncFIB	∼123 to ∼128 kb	Coverage: 100%; identity: ≥99%	FIB_N5_1	*E. coli* ST405 (2)
ȃIncFIB and IncFII	∼190 kb	Coverage: 100%; identity: ≥99%	FIB and FII_N5_1	*K. pneumoniae* ST11 (2)
ȃIncC	∼196 kb	…	C_N5_1	*K. pneumoniae* ST515 (1)
ȃIncFIB(pQil) and IncFII	∼203 kb	…	FIB(pQil) and FII_N5_1	*K. pneumoniae* ST11 (1)
IncFII and IncC	∼275 kb	…	FII and C_N5_1	ST515 (1)
NDM-1–positive plasmids				
ȃIncC	∼72 kb	…	C_N1_1	*K. pneumoniae* ST11 (1)
	∼154 to ∼174 kb	Coverage: 99% to 100%; identity: ≥99%	C_N1_2	*K. pneumoniae* ST395 (5)
	∼287 to ∼296 kb	Coverage: 97% to 100%; identity: ≥99%	C_N1_3	*P. stuartii* (4)
ȃIncFIB and IncHI1B	∼279 to ∼345 kb	Coverage: 84% to 100%; identity: ≥99%	**FIB and HI1B_N1_1**	*K. pneumoniae*: ST15 (3), ST15 (2), ST1998 (1)
ȃIncFIB(pQil)	∼119 kb	Coverage: 100%; identity: ≥99%	**FIB(pQil)_N1_1**	*K. variicola* (1), *K. pneumoniae* ST14 (1)
	∼135 kb	Coverage: 100%; identity: ≥99%	FIB(pQil)_N1_2	*K. pneumoniae* ST15 (2)
	∼163 kb	Coverage: 100%; identity: ≥99%	FIB(pQil)_N1_3	*K. pneumoniae* ST16 (2)
ȃIncFIA	∼141 kb	Coverage: 97%; identity: ≥99%	**FIA_N1_1**	*K. pneumoniae*: ST152 (1), ST16 (1)
ȃIncR	∼70 kb	…	R_N1_1	*K. pneumoniae* ST572 (1)
	∼152 kb	…	R_N1_2	*K. pneumoniae* ST17 (1)
ȃIncFIB and IncFII	∼215 kb	…	FIB and FII_N1_1	*K. pneumoniae* ST147 (1)
	∼206 kb	…	FIB and FII_N1_2	*K. pneumoniae* ST16 (1)
ȃIncFIB	∼150 kb	…	FIB_N1_1	*K. pneumoniae* ST16 (1)
ȃIncFIB and IncC	∼304 kb	…	FIB and C_N1_1	*K. pneumoniae* ST15 (1)
ȃIncFII	∼158 kb	…	FII_N1_1	*E. cloacae* (1)
ȃIncHI1A	∼182 kb	…	HI1A_N1_1	*E. cloacae* (1)
ȃIncHI1B	∼242 kb	…	HI1B_N1_1	*E. cloacae* (1)
ȃIncHI2	∼276 kb	…	HI2_N1_1	*E. coli* ST38(1)
ȃIncX3	∼58 kb	…	X3_N1_1	*E. cloacae* (1)
ȃUnknown	∼100 kb	…	un_N1_1	*Providencia stuartii* (1)
	∼100 kb	…	un_N1_2	*Serratia marcescens* (1)
NDM-7–positive plasmids				
ȃIncX3	∼46 kb	Coverage: 97% to 100%; identity: ≥99%	**X3_N7_1**	*E. coli*: ST101 (2), ST448 (1) *C. farmeri* (1) *E. cloacae* (1) *S. marcescens* (1)
NDM-4–positive plasmids				
ȃIncFIA	∼79 kb	Coverage: 99%; identity: ≥99%	FIA_N4_1	*E. coli* ST648 (3)
OXA-181–positive plasmids				
ȃIncX3	∼51 kb	…	X3_O181_1	*E. coli* ST410 (1)
ȃIncA/C2	∼182 kb	Coverage: 100%; identity: ≥99%	**A/C2_O181_1**	*E. coli*: ST2659 (1), ST8346 (1)
ȃIncFIC(FII)	∼79 kb	…	FIC(FII)_O181_1	*E. coli* ST448 (1)
OXA-232–positive plasmids				
ȃColKP3	∼61 kb	Coverage: 100%; identity: ≥99%	**ColKP3_O232_1**	*K. pneumoniae*: ST231 (3), ST15 (3)
ȃIncFIB(pQil)	∼134 kb	…	FIB(pQil)_O232_1	*K. pneumoniae* ST231 (1)

Abbreviations: Inc, incompatibility; NDM, New Delhi metallo-beta-lactamase; OXA, oxacillinases; ST, sequence type.

'n' indicates the number of isolates from which plasmids were characterized. Groups are underlined and bold according to whether horizontal transfer of plasmids was predicted for any group based on similarities of plasmids in a group and distribution of plasmids in wide range of species or wide clonal types.

To investigate the plasmid-mediated dissemination of carbapenem resistance, plasmids characterized in this study were grouped based on common carbapenemase allele, common Inc type, similar molecular weight, and similarities at ≥99% identity with >80% coverage between plasmids at the nucleotide level. Accordingly, NDM-5–positive plasmids fell into 21 groups, NDM-1–positive plasmids into 21 groups, OXA-181–positive plasmids into 3 groups, OXA-232–positive plasmids into 2 groups, 1 group of NDM-7, and 1 group of NDM-4–positive plasmids. Plasmid-mediated horizontal transfer of carbapenem resistance was predicted for the groups designated as FII_N5_2 (n = 7), FII_N5_3 (n = 31), X3_N5_1 (n = 9), FIB and HI1B_N1_1 (n = 6), FIB(pQil)_N1_1 (n = 2), FIA_N1_1 (n = 2), X3_N7_1 (n = 6), A/C2_O181_1 (n = 2), and ColKP3_O232_1 (n = 6), based on the distribution of plasmids within a wide range of bacterial hosts (Table 5, Supplementary Figures 7–14).

### Investigating the Possible Role of Mobile Genetic Elements in the Spread of Carbapenem Resistance

**Figure 7.**
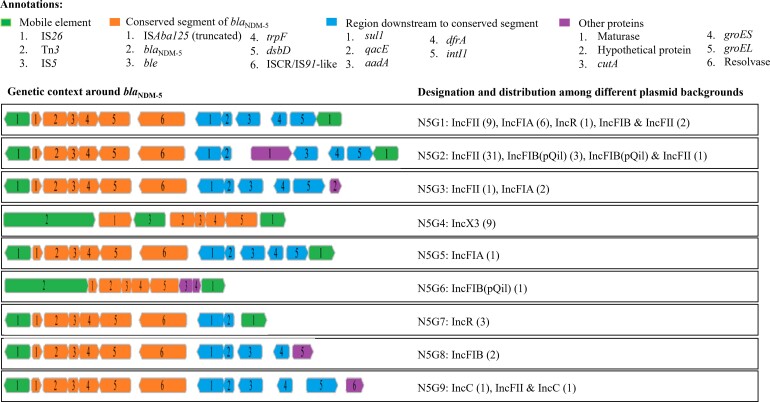
Schematic layout of genetic context around *bla*_NDM-5_ in different plasmid backgrounds. Arrows represent the position and transcriptional direction of the open reading frames. Accession numbers of specific plasmids’ sequences are stated in Supplementary Table 18. The layout of genetic context has been outlined using Geneious (v11.0.2).

Based on the variation in genes immediate to *bla*_NDM-5_, plasmids were divided into 9 groups, designated as N5G1 to N5G9 (Figure 7). A conserved region (incomplete IS*Aba125*, *bla*_NDM-5_, *ble*, *trpF*, *dsbD*, IS*91*) was common across all plasmids harboring *bla*_NDM-5_, except for N5G4 (IncX3) and N5G6 (IncFIB[pQil]), which lacked IS*91*. The conserved region of *bla*_NDM-5_ in association with complex class 1 integron ([*sul1*-*qacE*-*aadA*-*dfrA*- *intI*], or [*sul1*-*qacE*-Δmaturase-*aadA*-*dfrA*- *intI*]), flanked by intact IS*26* at both the 3′ and 5′ end in the same orientation, was found among plasmids of N5G1 and N5G2 (Figure 7).

**Figure 8.**
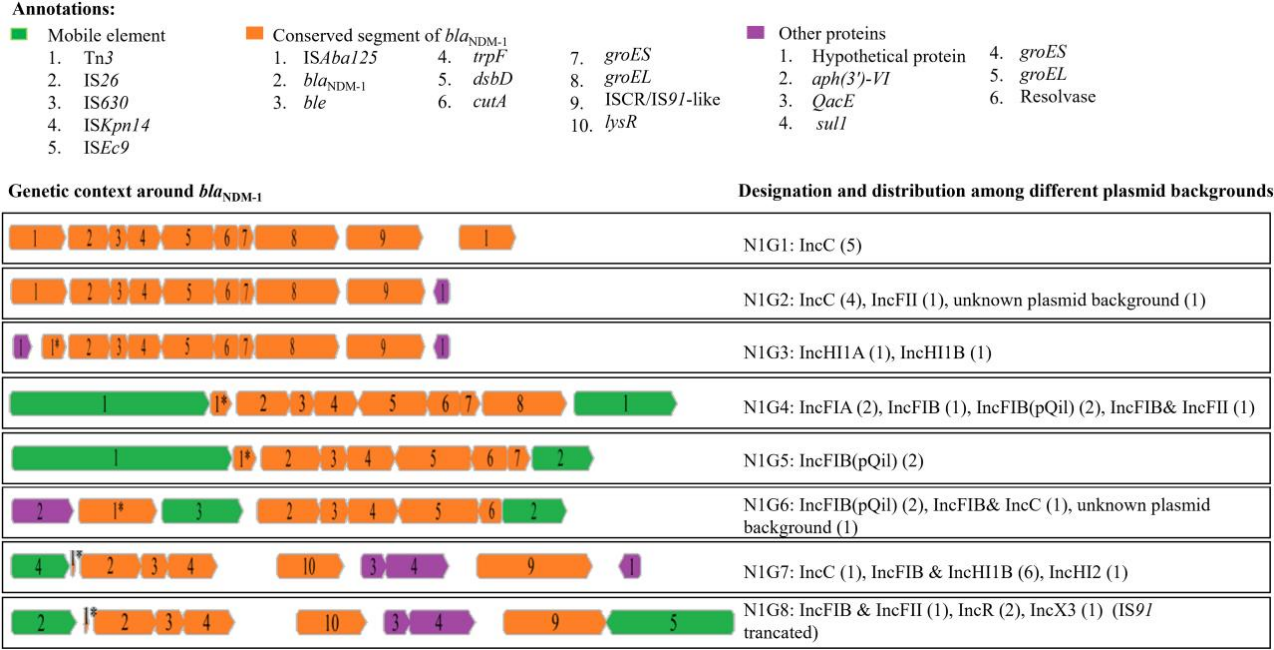
Schematic layout of genetic context around *bla*_NDM-1_ in different plasmid backgrounds. Arrows represent the position and transcriptional direction of the open reading frames. Truncated genes are denoted by “*”. Accession numbers of specific plasmids’ sequences are stated in Supplementary Table 18. The layout of genetic context has been outlined using Geneious (v11.0.2).

NDM-1–positive plasmids were divided into 8 groups (designated as NIG1 to N1G8) based on the genetic environment around *bla*_NDM-1_. The genetic structure Tn*125* (*bla*_NDM-1_*-ble*-*trpF-dsbD-cutA-groES-groEL-*IS*91*), bordered by intact IS*Aba125* at the upstream and downstream, was observed in plasmids of IncC (n = 5) (Figure 8). The insertion sequence IS*Aba125* was absent at the downstream of the plasmids of N1G2 to N1G8 and an incomplete IS*Aba125* was present at the upstream among plasmids of N1G3 to N1G8. Variation in genes in the conserved region was observed among the plasmids of N1G4 to N1G8. Plasmids of N1G4 were flanked by Tn*3* at both 3′ and 5′ ends in the same orientation (Figure 8).

## DISCUSSION

AMR collaborators provide a sobering analysis on the burden caused by common MDR/extremely drug-resistant extensively drug-resistant (XDR) infections and a warning that, as a global community, we are rapidly surrendering any advantage we had on treating infections such as pneumonia and sepsis. Furthermore, AMR collaborators highlight significant gaps, not least from low- and middle-income countries (LMICs), and advocate the acute need for large LMIC clinical studies that combine detailed microbiology (and genomics) with recorded outcome data [20]. In this study, we present the largest dataset comprising clinical outcome, microbiology, and genomic data from SA and, in particular, based in a large public hospital where such datasets capture different socioeconomic cohorts from previous studies (Supplementary Table 2). Public hospitals in SA are grossly oversubscribed (typically, 4–5 times the number of inpatients/beds), antibiotics are delivered empirically, and a limited number of clinical specimens are only sent for culture sensitivity (Supplementary Table 16) [21,22]. The problem of AMR is considerably higher in health settings with unsubscribed antimicrobial usage [23]. Our data revealed that *bla*_NDM_, *bla*_OXA-181_/*bla*_OXA-232_, and *bla*_KPC-2_ were main carbapenem resistance determinants in CRE in Bangladesh. New Delhi metallo-beta-lactamase (NDM)-positive plasmids except for IncX3 commonly co-harbored multiple ARGs (Supplementary Figure 6). The association of CRE acquisition with the usage of multiple antibiotic classes may be explained by co-selection in hospital settings, both of MDR CRE clones and their key MDR plasmids (Table 2) [9,23,24]. Moreover, several factors, such as introduction of artificial devices, long-term antibiotic exposure, prolonged hospital stays, and clinical comorbidities, can be responsible for CRE acquisition among patients in burn units and the ICU [25–27].

Previous reports suggest that CRE was associated with a 3-fold greater mortality than CSE cases [2–4]. This study recorded substantially higher mortality with CRE (27.8%) than CSE cases (13.5%) (*P* = .001), including a cohort with positive culture of Enterobacterales from both sterile (blood) and nonsterile sampling sites (wound swabs, tracheal aspirates, etc). Given the level of statistical significance in adjusted models, it was possible that comorbidities might influence mortality among patients in the ICU and burn units (Table 3) [25–27]. Worse patient outcomes are invariably associated with limited therapeutic options [2,4,28,29]. We demonstrated that 73% of the patients did not receive at least 1 appropriate antibiotic (Supplementary Table 10). However, this study was unable to conclude whether ineffective antibiotic therapy or any other confounders influenced mortality among the patients with CRE due to the limited number of BSIs and limited clinical information (eg, comorbidities or antibiotic therapy).

To date, the presence of *bla*_NDM_ in different clonal lineages has mainly been reported from China, Europe, or the United States and, as such, there is a bias in the global reporting of their geographical distribution [30]. This study documented numerous prevalent clones with *bla*_NDM_ (ST167, ST648, ST448, and ST405 of *E. coli*, and ST15, ST23, ST147, and ST16 of *K. pneumoniae*) (Figures 4 and 5, Supplementary Figures 1–5). Additionally, *E. coli* ST8346 was recognized as a newly emerging clone carrying *bla*_NDM-1_ (Supplementary Figure 2, Supplementary Table 1^1^).

Based on spatiotemporal analysis, it is possible that CRE clones had been established at DMCH over an extended period of time. The average substitution rates of these clones (3 SNPs/genome/year) were in line with previous reports (Supplementary Table 1^3^) [31,32]. We observed the presence of common carbapenemases among the isolates differing by ≤10 SNPs, some of which represented tight clades (0–2 SNP threshold), combined with evidence of a common ward of isolation and overlapping of patients’ hospital stay (Figure 6), suggesting potential recent transmission or acquisition of clones from a common source. The number of such events was considerably higher among patients with *K. pneumoniae* infections compared with other species (Figure 6), indicating higher transmissibility of *K. pneumoniae* [33]. Of particular concern was the spread of *bla*_NDM-5_ via a highly virulent *K. pneumoniae* ST23 (KP12) clone having the KL1 locus [34].

This study documented the plasmid-mediated horizontal dissemination of *bla*_NDM_ among different species of Enterobacterales, mostly by IncFII and IncX3 (Table 5, Supplementary Figures 9 and 13). IncX3 is widely spread throughout China and SA and associated with *bla*_NDM_, particularly, *bla*_NDM-5_ [35,36. However we found plasmids of a wide variety of Inc types (IncFII IncFIA IncR IncFIB and IncFII IncFIBpQil], and IncFIB(pQil) and IncFII) harboring *bla*_NDM-5_, yet that the plasmids had identical conserved regions followed by a class 1 integron together flanked by IS*26* (Figures 7 and 8). It can therefore be hypothesized that transposition of the IS*26*-flanked segment occurred via 2-step recombination where IS*26* released a DNA segment from a donor plasmid and IS*91* facilitated its insertion into the recipient plasmid by rolling circle replication [37,38].

A limitation of this study was the inability to capture all possible Enterobacterales infections due to the practice norms at DMCH in terms of microbiological sampling. These are typical of many public hospitals in SA. However, this study represents the most comprehensive report on the epidemiology and mechanism of CRE in Enterobacterales in SA. This study has also (1) shown the high burden of CRE compared with previously reported studies, (2) provided data on outcome and inappropriate antibiotic use that will inform better antibiotic stewardship programs including antibiotic access and affordability in the public sector, and (3) demonstrated genomic evidence on the clonal spread of virulent CRE, prioritizing infection-control programs in limited financial settings. While this study was conducted in the largest public hospital in Bangladesh, many of these findings can be extrapolated across SA, which encompasses a population of nearly 2 billion and signals the need for greater engagement and targeted investment.

## Supplementary Material

ciac287_Supplementary_Data

## Supplementary Data

Supplementary materials are available at *Clinical Infectious Diseases* online. Consisting of data provided by the authors to benefit the reader, the posted materials are not copyedited and are the sole responsibility of the authors, so questions or comments should be addressed to the corresponding author.

## Notes

***Author Contributions.*** R. F. and T. R. W contributed equally to the work. R. F., L. S. J., and T. R. W. designed the study; R. F. and T. R. W. obtained funding; R. F., M. A. R., M. P., and M. A. K. were involved with project administration and data collection; R. F., K. S., E. P., I. B., B. H., and J. M. performed laboratory work; R. F. and W. J. W. performed the statistical analysis; R. F., K. S., A. J. v. T., J. M. C., J. P., and M. F. G. performed the bioinformatic analysis; R. F., L. S. J., A. J. v. T., M. A. K., and T. R. W. verified the data and drafted the manuscript. All authors critically reviewed the manuscript and approved the final report before submission. R.F. conducted this study as part of her PhD project.

***Acknowledgments.*** The authors thank Prof Ismail Khan, Former Principal, Dhaka Medical College, for providing access to collecting clinical data and undertaking initial processing of clinical samples at Dhaka Medical College. The authors are grateful to Prof Abul Kalam for allowing sampling from the burns unit of Dhaka Medical College Hospital.

***Data availability.*** The study methods, statistical and bioinformatics analysis are available in detail in the main text and supplementary material. Genomes of Enterobacterales have been deposited in NCBI under the BioProject number of PRJNA722682, PRJNA719593, and PRJNA714521.

***Disclaimer.*** The funder of the study had no role in study design, data collection, data analysis, data interpretation, or writing of the report

***Financial support.*** R. F. is the recipient of a Commonwealth Scholarship (BDCS-2016-53). Sequencing and molecular data were supported by Cardiff University.
