# Supplementary material for: Genomic Insights Into the Mechanism of Carbapenem Resistance Dissemination in Enterobacterales From a Tertiary Public Heath Setting in South Asia
Source: Clin Infect Dis. 2022 Apr 27;76(1):119–33. doi: 10.1093/cid/ciac287 (PMC9825829; doi:10.1093/cid/ciac287)
Supplement: ciac287_Supplementary_Data [file ciac287_supplementary_data.zip › 03_DMCH_paper_supplementary_Table_clean_04032022.docx]

**Supplementary Table 1. The recent update of AMR surveillance in the countries of SA according to GLASS Early Implementation Report 2020.**

| Country | NAP | NCC | Surveillance site | Data on infection origin | Data on number of tested patients | Carbapenem resistance [NR/NT] | Colistin resistance [NR/NT] | Current surveillance status |
| --- | --- | --- | --- | --- | --- | --- | --- | --- |
| Afghanistan | NP | Established | One | Reported | No data reported | 0/10 (IPM-S) | NA | The country is working to enhance diagnostic capacity and to expand the number of participating surveillance sites and laboratories in coming years. |
| Bangladesh | P | Established | Eight | No data reported | No data reported | 88/469 (IPM-S) | NA | IEDCR is conducting AMR surveillance implemented as case-based surveillance of clinical syndromes. |
| Bhutan | P | Established | Three | - | - | NA | NA | No AMR data reported to GLASS. |
| India | P | Established | 130 | <70% reported | Reported | 5780/13751 (IPM-S); 6280/13892 (MEM-S) | 0/107 | There are 3 AMR surveillance networks participating in the National AMR Surveillance in the country. 1. National AMR Surveillance network, NCDC. 2. Antimicrobial Surveillance and Research network, ICMR; 3. Gonococcal Antimicrobial Resistance surveillance network, Safdarjung Hospital. |
| Maldives | P | Established | Four | Reported | Reported |  |  | All national surveillance sites reported to GLASS |
| Nepal | P | Established | 21 | <70% reported | Reported | 236/899 (IPM-S); 269/1675 (MEM-S) | NA | AMR surveillance started in Nepal since 1999 with six participating laboratories/hospitals. The network has extended and includes 21 hospitals. |
| Pakistan | P | Established | 10 | <70% reported | No data reported | 629/8406 (IPM-S); 511/4037 (MEM-S) | NA | All national surveillance sites reported to GLASS |
| Sri Lanka | P | Established | 17 | No data reported | Reported | NA | NA | National AMR Surveillance System for public as well as the private sectors has been established in 2017. Each sector will develop their own surveillance system under One Health approach. |

IEDCR, Institute of Epidemiology Disease Control and Research; IPM-S, imipenem susceptibility; MEM-S, meropenem susceptibility; NA, no data available; NAP, National Action Plan; NCC, National Coordinating Centre; NP, not in place; NR, number of resistant strains; NT, number of tested strains by antimicrobial susceptibility testing (AST). P, place.

**Supplementary Table 2. Scoping findings relevant to this study from previous literature searches.**

| Article attributes | Country | Type of hospital* | Sample size | Species | Keynotes for relevant findings | Relevant findings |
| --- | --- | --- | --- | --- | --- | --- |
| Stewardson et al. Lancet Infect Dis. 2019;19(6):601-610. | Multinational (LMICs) | Public & private | 297 | Enterobacterales | Mortality assessment | Carbapenem resistant bloodstream infections (BSIs) were significantly associated with increased mortality and increased length of hospital stay. Investigation of clonal relatedness based on Multilocus sequence type (MLST). |
| Snyder et al. Epidemiol Infect. 2019;147:e137. | India | Private | 213 | *Klebsiella pneumoniae* | Risks assessment | CVC placement, prior carbapenem use and ICU admission were the risk for the development of BSI with NDM-1 producing and other MDR strains. |
| Choudhuri et al. Saudi J Anaesth. 2018;12(3):389-394. | India | Public | 106 | MDR bacteria | Mortality and risks assessment | Significant higher mortality was found in MDR group. The independent predictors of MDR bacterial infection were Child-Pugh score >10, prior carbapenem use, antibiotic use for more than 10 days, total parenteral nutrition, and concurrent antifungal administration. |
| Shankar et al. J Assoc Physicians India. 2018;66(12):13-16. | India | Private | 86 | *K. pneumoniae* | Mortality assessment | Significant higher mortality was observed in carbapenem resistant *K. pneumoniae*. There were also associations between hypermucoviscous *K. pneumoniae* and mortality. |
| Naim et al. J Glob Infect Dis. 2018;10(3):133-139. | India | Public | 116 | Gram-negative Bacilli | Risks assessment | Major risk factors in patients infected with MBLs were in-dwelling devices, prolonged hospital stay, and prior antibiotic treatment, but the risks were not assessed with any comparator. |
| Kaur et al. Am J Infect Control. 2017;45(11):1289-1291. | India | Private | 75 | *K. pneumoniae* | Prevalence of mortality in CRE | 5-year survey of dual colistin- and carbapenem-resistant BSIs showed high mortality (69.3%). |
| Mariappan et al. Int J Appl Basic Med Res. 2017;7(1):32-39. | India | Private | 111 | Enterobacterales | Mortality and risks assessment | Carbapenem resistance had significant association with mortality among the patients with mechanical ventilation and indwelling invasive device. |
| Kumar et al. J Clin Diagn Res. 2015;9(11):DC08-DC13. | India | Public | 186 | *Escherichia coli* and *Klebsiella* Spp. | Risks assessment | Co-morbidity, ICU admission, and administration of artificial device were shown to risks for infections by NDM-1 producers. |
| Kalam et al. J Pak Med Assoc. 2014;64(5):530-536. | Pakistan | Public | 243 | Gram-negative bacteria | Mortality and risks assessment | MDR bacteraemia is a significant risk for mortality. Risk factors for carbapenem resistant bacteraemia were age > 50 years, septic shock on presentation, ICU stay of > 72 hours, and receiving immunosuppressant medications. |
| Ramanathan et al. Indian J Med Microbiol. 2018;36(4):572-576. | India | Private | 102 | Enterobacterales | Risks assessment | Data showed that the development of infections following CRE colonisation in critical care unit. Patients exposed to high end antibiotic and past history of surgery had significant association with CRE colonization |
| Singh et al. Am J Infect Control. 2018;46(6):e31-e35. | India | Public | 300 | Enterobacterales | Risks assessment | Statistically significant risk factors of CRE colonisation of neonates in NICU were found to be NG tube, breastfeeding, NG feeding, top feeding, expressed breastmilk, ventilation, antibiotic administration, and duration of hospitalization |
| Bharadwaj et al. BMC Infect Dis. 2018;18(1):504. Published 2018 Oct 4. | India | Public | 897 | Enterobacterales | Risks assessment | CRE colonisation was significantly associated with recent healthcare and ICU admission. |
| Mohan et al. Indian J Med Microbiol. 2017;35(4):555-562. | India | Public | 232 | Enterobacterales | Risks assessment | ICU admission, administration of indwelling device, and nasogastric tube were the independent risks for CRE colonisation. |
| Mittal et al. BMC Microbiol. 2016;16(1):138. | India | Public | 100 | Enterobacterales | Risks assessment | The risk factors associated with CRE carriage were duration of ICU stay, use of ventilator and aminoglycosides |
| Datta et al. Indian J Med Microbiol. 2015;33(4):612-613. | India | Public | 75 | Enterobacterales | Risks assessment | Patients with CRE colonisation in ICU were more likely to be associated with co-morbidity, prior surgery, intrahospital transfer, and prior exposure to carbapenem, cephalosporin, fluoroquinolones, and metronidazole. |
| Naha et al. Int J Antimicrob Agents. 2020;55(3):105903. | India | Public | 4 | *K. pneumoniae* | Molecular characterization by WGS | Characterization of KPC producers by WGS. |
| Shankar et al. BMC Microbiol. 2019;19(1):137. | India | Private | 49 | *K. pneumoniae* | Molecular characterization by WGS | Clonal relatedness of isolates harbouring *bla*_OXA-48-like_ and genetic background of OXA-48-like using WGS. Genetic distance based on SNP was not revealed. |
| Shankar et al. J Infect Public Health. 2019;12(5):741-743. | India | Private | One | *K. pneumoniae* | Molecular characterization by WGS | Characterization of an isolate of NDM producer by WGS. |
| Zhu et al. Front Microbiol. 2018;9:2044 | Sri Lanka | Public | 379 | *K. pneumoniae* | Molecular characterization by WGS | Transmission dynamics of OXA-181 producers by WGS. |
| Lomonaco et al. PLoS One. 2018;13(6):e0198526. | Pakistan | Private | 10 | *K. pneumoniae* | Molecular characterization by WGS | Transmission dynamics of 10 carbapenemase producers by WGS. |
| Shankar et al. J Med Microbiol. 2018;67(7):927-930. | India | Private | One | *K. pneumoniae* | Molecular characterization by WGS | Characterization of KPC producers by WGS. |
| Shrestha et al. Antimicrob Agents Chemother. 2017;61(12):e01425-17. | Nepal | Public | 250 | *E. coli* | Molecular characterization by WGS | Clonal relatedness of isolates harbouring variants of *bla*_NDM_, revealed by WGS. Genetic distance based on SNP was not revealed. |
| Subramanian et al. J Glob Antimicrob Resist. 2017;8:121-122. | India | Public | One | *E. coli* | Molecular characterization by WGS | Characterization of an isolate of NDM producer by WGS. |
| Nahid et al. PLoS One. 2017;12(12):e0189438. | Pakistan | Public | One | *K. pneumoniae* | Molecular characterization by WGS | Characterization of an isolate of OXA-181 producer by WGS. |
| Ranjan et al. Antimicrob Agents Chemother. 2016;60(11):6795-6805. | India | Public | 510 | *E. coli* | Molecular characterization by WGS | Distribution of *bla*_NDM_ in diverse STs of *E. coli*. Typing was done ERIC-PCR along with WGS of 5 isolates. |
| Wailan et al. Antimicrob Agents Chemother. 2015;59(12):7405-7410. | Pakistan | Unknown | Four | Gram-negative bacteria | Molecular characterization by WGS | Characterization of 11 NDM-1 producers isolated from stool of four patients. WGS was deployed to characterize the isolates. |
| Stoesser et al. Antimicrob Agents Chemother. 2014;58(12):7347-7357. | Nepal | Private | 94 | *K. pneumoniae* | Molecular characterization by WGS | Clonal relatedness and transmission dynamic of K. pneumoniae by WGS following an outbreak in a neonatal unit. Genetic distance based on SNP was revealed. |
| McGann et al. Antimicrob Agents Chemother. 2012;56(4):1673-1679. | Afghanistan | U.S./Coalition medical facility | One | *Providencia stuartii* | Molecular characterization by WGS | Characterization of an isolate of NDM producer by WGS. |
| Wangkheimayum et al. BMC Infect Dis. 2020;20(1):544. | India | Public | 329 | *E. coli* | Characterization by molecular approaches other than WGS | Characterization of NDM and OXA-48 producers by MLST and PBRT. |
| Gondal et al. Infect Drug Resist. 2020;13:2105-2115. | Pakistan | Public | 227 | *K. pneumoniae* | Characterization by molecular approaches other than WGS | Evidence of clonal and non-clonal dissemination of NDM, and OXA-48 producers, demonstrated by MSLT. |
| Khalid et al. Microb Drug Resist. 2020;26(3):284-289. | India | Unknown | 18 | Gram-negative bacteria | Characterization by molecular approaches other than WGS | Prevalence of NDM producers in a neonatal unit along with probable spread of carbapenem resistance by plasmid. |
| Mukherjee et al. Infect Genet Evol. 2019;69:166-175. | India | Public | 200 | *K. pneumoniae* | Characterization by molecular approaches other than WGS | Associations of *bla*_NDM_ in diverse STs of *K. pneumoniae* from neonatal septicaemia, revealed by REP-PCR, PFGE, and MLST. |
| Ahmad et al. Int J Antimicrob Agents. 2019;53(4):525-529. | India | Public | 14 | *K. pneumoniae* | Characterization by molecular approaches other than WGS | MLST based clonal relatedness with characterization of genetic context and plasmid of NDM producers from a neonatal unit. |
| Qamar et al. Future Microbiol. 2019;14:691-704. | Pakistan | Public | 117 | *E. coli*, and *K. pneumoniae* | Characterization by molecular approaches other than WGS | Transmission dynamics of carbapenemase producers by PFGE and DNA hybridization. |
| Remya et al. J Lab Physicians. 2019;11(4):312-316. | India | Private | 370 | *K. pneumoniae* | Characterization by molecular approaches other than WGS | Clonal relatedness of NDM and KPC producers by PFGE. |
| Choudhury et al. J Infect Public Health. 2018;11(1):111-114. | India | Public | One | *E. coli* | Characterization by molecular approaches other than WGS | Clonal relatedness of NDM-4 producers, revealed by MLST and PFGE along with plasmid characterization. |
| Rahman et al. J Glob Antimicrob Resist. 2018;14:154-157. | India | Public | 33 | *E. coli* | Characterization by molecular approaches other than WGS | Clonal relatedness of NDM producers. |
| Ahmad et al. Microb Drug Resist. 2018;24(2):161-165. | India | Public | 402 | Enterobacterales | Characterization by molecular approaches other than WGS | Characterize genetic context of NDM producers and plasmid typing by PBRT. |
| Gajamer et al. J Glob Antimicrob Resist. 2018;14:228-232. | India | Public | 973 | *E. coli* | Characterization by molecular approaches other than WGS | Characterization of plasmids harbouring *bla*_NDM_, isolated from urine specimens. |
| Ahmad et al. Front Microbiol. 2018;9:407. | India | Public | 44 | Enterobacterales | Characterization by molecular approaches other than WGS | Characterization of plasmids harbouring *bla*_NDM_. |
| Paul et al. Microb Drug Resist. 2017;23(7):815-821. | India | Public | 900 | Carbapenem resistant bacteria | Characterization by molecular approaches other than WGS | Evaluated the horizontal transmission of NDM producers. |
| Paul et al. J Infect Chemother. 2017;23(4):206-210. | India | Public | Six | *E. coli* | Characterization by molecular approaches other than WGS | Characterization of plasmids harbouring blaNDM-7. |
| Khan et al. J Pak Med Assoc. 2016;66(8):999-1004. | Pakistan | Private | 114 | Enterobacterales | Characterization by molecular approaches other than WGS | Clonal relatedness of NDM-1 producers by tandem repeat analysis. |
| Subramanian et al. Indian J Med Microbiol. 2016;34(3):286-292. | India | Public | Three | *K. pneumoniae* | Characterization by molecular approaches other than WGS | Characterization of plasmids harbouring *bla*_NDM_. |
| Krishnaraju et al. Indian J Med Microbiol. 2015;33(1):30-38. | India | Private | 76 | Enterobacterales | Characterization by molecular approaches other than WGS | Characterization of NDM producers. |
| Pesesky et al. Emerg Infect Dis. 2015;21(6):1034-1037. | Pakistan | Public & private | 78 | Enterobacterales | Characterization by molecular approaches other than WGS | Characterization of plasmid harbouring *bla*_NDM-1_ and *bla*_KPC_ from Pakistan and USA by sequencing of plasmid. |
| Hall et al. J Med Microbiol. 2014;63(Pt 8):1087-1092. | Sri Lanka | Public | 22 | *K. pneumoniae* | Characterization by molecular approaches other than WGS | Clonal relatedness of carbapenemase producers by PFGE. |
| Sartor et al. Antimicrob Agents Chemother. 2014;58(9):5589-5593. | Pakistan | Military biodefense | 66 | Enterobacterales | Characterization by molecular approaches other than WGS | Clonal spread of NDM producers, revealed by rep-PCR. |
| Khajuria et al. Indian J Pathol Microbiol. 2014;57(1):65-68. | India | Public & private | Six | *K. pneumoniae* | Characterization by molecular approaches other than WGS | Outbreak caused by NDM producers in a neonatal unit. |
| Rahman et al. Int J Antimicrob Agents. 2014;44(1):30-37. | India | Public | 464 | Enterobacterales | Characterization by molecular approaches other than WGS | Characterization of plasmid harbouring *bla*_NDM_. |
| Khajuria et al. Chemother Res Pract. 2014;2014:972646. | India | Public | 130 | Enterobacter Species | Characterization by molecular approaches other than WGS | Clonal relatedness and plasmid characterization of NDM producers. |
| Khajuria et al. J Clin Diagn Res. 2014;8(6):DC01-DC4. | India | Public | 300 | *E. coli* | Characterization by molecular approaches other than WGS | Characterization of co-producing NDM-1 and OXA-48 carbapenemases by ERIC-PCR and PBRT. |
| Castanheira et al. Diagn Microbiol Infect Dis. 2013;75(2):210-213. | India | Private | 13 | Enterobacterales | Characterization by molecular approaches other than WGS | Characterization of plasmids harbouring *bla*_NDM_. |
| Islam et al. Eur J Clin Microbiol Infect Dis. 2012;31(10):2593-2600. | Bangladesh | Private | 1,816 | Gram-negative bacteria | Characterization by molecular approaches other than WGS | Characterization of NDM producers by PFGE. |
| Castanheira et al. Antimicrob Agents Chemother. 2011;55(3):1274-1278. | India | Public & private | 39 | Enterobacterales | Characterization by molecular approaches other than WGS | Clonal dissemination of carbapenemase producing bacteria, revealed by PFGE. |
| Roy et al. J Antimicrob Chemother. 2011;66(12):2773-2780. | India | Public | 99 | *E. coli* | Characterization by molecular approaches other than WGS | Clonal relatedness NDM producers by PFGE, phenotyping (A, B1, B2 and D), and based on virulence factors (*hly, papC, sfa, iroNE. coli, cnf1,* *iucC* and *ibeA*). |

CRE, carbapenem-resistant Enterobacterales; NR, not reported. *Type of hospital from where samples were collected.

**Supplementary Table 3. Total number of culture-positive clinical specimens from different wards of DMCH in this study (n=1893*).**

| Ward | Blood (n=527) | | | CT (n=6) | | | CV tip (n=7) | | | TA (n=99) | | | Urine (n=442) | | | WS (n=809) | | | Total |
| --- | --- | --- | --- | --- | --- | --- | --- | --- | --- | --- | --- | --- | --- | --- | --- | --- | --- | --- | --- |
|  | S | M | NG | S | M | NG | S | M | NG | S | M | NG | S | M | NG | S | M | NG |  |
| Burn | 90 | 45 | 121 | - | 3 | - | 1 | - | - | - | - | - | 4 | 3 | 6 | 122 | 110 | 172 | 677 |
| Casualty unit | - | - | 1 | - | - | - | - | - | - | - | - | - | - | - | 1 | 6 | 4 | 9 | 21 |
| CCU | - | - | - | - | - | - | - | - | - | - | - | - | - | - | 1 | - | - | - | 1 |
| Dialysis Unit | - | - | - | - | - | - | - | 1 | - | - | - | - | - | - | - | - | - | - | 1 |
| Fistula unit | - | - | - | - | - | - | - | - | - | - | - | - | 31 | 11 | 7 | - | 1 | - | 50 |
| Gynaecology | 5 | - | 2 | - | - | - | - | - | - | - | - | - | 5 | 5 | 4 | 33 | 11 | 18 | 83 |
| Haematology | 6 | 1 | 9 | - | - | 1 | - | - | - | - | - | - | 5 | 4 | 3 | 1 | - | 3 | 33 |
| HDU | 1 | 1 | 2 | - | - | - | - | - | 1 | 3 | 1 | 2 | 1 | - | 5 | 2 | 1 | 7 | 27 |
| ICU | 8 | 6 | 11 | - | 1 | - | 2 | - | - | 32 | 47 | 14 | 6 | 10 | 16 | 1 | 1 | 1 | 156 |
| Medicine | 6 | - | 5 | - | - | - | - | - | - | - | - | - | 7 | - | 20 | 4 | 1 | 10 | 54 |
| Nephrology | 1 | - | - | - | - | - | - | - | - | - | - | - | 8 | 5 | 14 | - | - | - | 28 |
| Neurology | 1 | - | - | - | - | - | - | - | - | - | - | - | - | - | - | - | - | - | 1 |
| Neurosurgery | - | - | 6 | - | - | - | - | - | - | - | - | - | 1 | - | - | 1 | 2 | - | 10 |
| NICU | 43 | 12 | 118 | - | - | - | - | - | - | - | - | - | - | - | - | - | - | - | 173 |
| OCC | - | - | - | - | - | - | - | - | - | - | - | - | - | - | - | 1 | - | - | 1 |
| Oncology | - | - | - | - | - | - | - | - | - | - | - | - | 1 | - | - | - | - | 1 | 2 |
| Orthopaedics | 1 | - | 2 | - | - | - | - | - | - | - | - | - | 2 | 2 | 1 | 27 | 12 | 7 | 54 |
| PS | 3 | 1 | 2 | - | - | 1 | - | - | - | - | - | - | 12 | 6 | 13 | 5 | 2 | 4 | 49 |
| Paediatrics | 2 | 1 | 9 | - | - | - | - | - | - | - | - | - | - | - | 11 | 1 | 1 | 8 | 33 |
| Postnatal | - | - | - | - | - | - | - | - | - | - | - | - | - | - | - | - | - | 1 | 1 |
| Surgery | 3 | - | 3 | - | - | - | - | 1 | 1 | - | - | - | 8 | 1 | 8 | 99 | 45 | 70 | 239 |
| Urology | - | - | - | - | - | - | - | - | - | - | - | - | 70 | 62 | 62 | 2 | 2 | - | 198 |
| Total | 170 | 67 | 291 | - | 4 | 2 | 3 | 2 | 2 | 35 | 48 | 16 | 161 | 109 | 172 | 305 | 193 | 311 | 1,891* |

Legend. CT, catheter tip; CV, central venous; TA, tracheal aspirate; WS, wound swab; S, single growth; M, mixed growth; NG, no growth; CCU, coronary care unit; HDU, high dependency unit; ICU, intensive care unit; NICU, neonatal intensive care unit; OCC, one-stop crisis centres; PS, paediatric surgery. *One bile and one urethral discharge were collected which were culture negative and positive, respectively.

**Supplementary Table 4. The frequency of isolation of CRE and CSE from different clinical samples.**

| Sample types | CR | CS | Total |
| --- | --- | --- | --- |
| WS | 81 | 183 | 264 |
| Urine | 50 | 155 | 205 |
| Blood | 48 | 65 | 113 |
| TA | 26 | 28 | 54 |
| Catheter tip | 4 | 1 | 5 |
| UD | 1 | 0 | 1 |
| CV tip | 0 | 1 | 1 |
| Total | 210 | 433 | 643 |

Legend. CT, catheter tip; CV, central venous; UD, urethral discharge; TA, tracheal aspirate; WS, wound swab.

**Supplementary Table 5. Total number of clinical isolates identified in this study (n=1583).**

| Organisms | Blood | CT | CV tip | TA | UD | Urine | WS | Total |
| --- | --- | --- | --- | --- | --- | --- | --- | --- |
| *Acinetobacter baumannii* | 36 | 1 | 1 | 32 | 0 | 15 | 84 | 169 |
| *Acinetobacter* spp. (other than *A. baumannii*)* | 8 | 0 | 0 | 4 | 0 | 15 | 5 | 32 |
| B*urkholderia* spp.* | 19 | 0 | 0 | 1 | 0 | 1 | 0 | 21 |
| *Candida* spp.* | 6 | 0 | 0 | 0 | 0 | 7 | 2 | 15 |
| *Citrobacter freundii* | 0 | 0 | 0 | 0 | 0 | 1 | 2 | 3 |
| *Citrobacter koseri* | 0 | 0 | 0 | 0 | 0 | 1 | 0 | 1 |
| *Citrobacter* spp.** | 2 | 0 | 0 | 0 | 0 | 0 | 4 | 6 |
| *Citrobacter youngae* | 0 | 0 | 0 | 0 | 0 | 0 | 1 | 1 |
| *Corynebacterium striatum* | 2 | 0 | 0 | 0 | 0 | 1 | 8 | 11 |
| *Entercoccus* spp.* | 23 | 1 | 2 | 11 | 0 | 57 | 74 | 168 |
| *Enterobacter bugandensis* | 0 | 0 | 0 | 0 | 0 | 0 | 1 | 1 |
| *Enterobacter cloacae* complex | 8 | 0 | 0 | 1 | 0 | 9 | 21 | 39 |
| *Escherichia coli* | 16 | 2 | 0 | 10 | 0 | 117 | 81 | 226 |
| *Escherichia hermannii* | 1 | 0 | 0 | 0 | 0 | 0 | 0 | 1 |
| *Klebsiella aerogenes* | 0 | 0 | 0 | 0 | 0 | 0 | 2 | 2 |
| *Klebsiella oxytoca* | 0 | 0 | 0 | 0 | 0 | 3 | 1 | 4 |
| *Klebsiella pneumoniae* | 43 | 3 | 1 | 29 | 1 | 52 | 92 | 221 |
| *Klebsiella quasipneumoniae* | 1 | 0 | 0 | 1 | 0 | 2 | 4 | 8 |
| *Klebsiella variicola* | 14 | 0 | 0 | 0 | 0 | 0 | 0 | 14 |
| *Leclercia adecarboxylata* | 0 | 0 | 0 | 0 | 0 | 1 | 0 | 1 |
| *Morganella morganii* | 0 | 0 | 0 | 0 | 0 | 5 | 1 | 6 |
| *Pantoea anthophila* | 0 | 0 | 0 | 1 | 0 | 0 | 0 | 1 |
| *Proteus mirabilis* | 10 | 0 | 0 | 8 | 0 | 7 | 39 | 64 |
| *Proteus terrae* | 0 | 0 | 0 | 0 | 0 | 2 | 0 | 2 |
| *Proteus vulgaris* | 1 | 0 | 0 | 0 | 0 | 0 | 0 | 1 |
| *Providencia rettgeri* | 0 | 0 | 0 | 0 | 0 | 0 | 1 | 1 |
| *Providencia* spp.** | 0 | 0 | 0 | 0 | 0 | 2 | 2 | 4 |
| *Providencia stuartii* | 5 | 0 | 0 | 1 | 0 | 0 | 11 | 17 |
| *Providencia thailandensis* | 1 | 0 | 0 | 0 | 0 | 0 | 0 | 1 |
| *Pseudomonas aeruginosa* | 65 | 1 | 1 | 24 | 1 | 60 | 203 | 355 |
| *Pseudomonas* spp. (other than *P. aeruginosa*)* | 5 | 1 | 0 | 0 | 0 | 6 | 14 | 26 |
| *Salmonella* spp.** | 5 | 0 | 0 | 0 | 0 | 0 | 0 | 5 |
| *Serratia marcescens* | 6 | 0 | 0 | 2 | 0 | 2 | 1 | 11 |
| *Serratia ureilytica* | 0 | 0 | 0 | 1 | 0 | 1 | 0 | 2 |
| *Staphylococcus aureus* | 3 | 0 | 1 | 0 | 0 | 2 | 39 | 45 |
| *Staphylococcus* spp. (other than *S. aureus*)* | 22 | 0 | 0 | 0 | 0 | 7 | 10 | 39 |
| *Stenotrophomonas maltophilia* | 1 | 0 | 0 | 10 | 0 | 4 | 2 | 17 |
| Others | 8 | 0 | 1 | 4 | 0 | 19 | 10 | 42 |
| Total | 311 | 9 | 7 | 140 | 2 | 399 | 715 | 1583 |

Legend. CT, catheter tip; CV, central venous; TA, tracheal aspirate; UD, urethral discharge; WS, wound swab. *The organisms at the species level identified were: *A. bereziniae* (n=9), *A. junii* (n=6), *A. pittii* (n=5), *A. schindleri* (n=3), *Acinetobacter ursingii* (n=3), *Acinetobacter baylyi* (n=2), *A. radioresistens* (n=2), *Acinetobacter towneri* (n=2), *Burkholderia cepacia* (n=16), *Burkholderia cenocepacia* (n=4), *Burkholderia gladioli* (n=1), *Candida tropicalis* (n=9), *Candida albicans* (n=4), *Candida auris* (n=2), *Enterococcus faecalis* (n=116), *Enterococcus faecium* (n=39), *Enterococcus gallinarum* (n=6), *Enterococcus hirae* (n=3), *Enterococcus avium* (n=2), *Enterococcus casseliflavus* (n=2), *Enterococcus raffinosus* (n=1), *Enterococcus thailandicus* (n=1), *Pseudomonas mendocina* (n=11), *P. putida* (n=6), *Pseudomonas mosselii* (n=2), *Pseudomonas oleovorans* (n=2), *Pseudomonas brassicacearum* (n=1), *Pseudomonas composti* (n=1), *Pseudomonas guariconensis* (n=1), *Pseudomonas pseudalcaligenes* (n=1), *Pseudomonas stutzeri* (n=1), *Staphylococcus haemolyticus* (n=19), *Staphylococcus sciuri* (n=6), *Staphylococcus epidermidis* (n=5), *Staphylococcus hominis* (n=3), *Staphylococcus arlettae* (n=2), *Staphylococcus cohnii* (n=2), *Staphylococcus capitis* (n=1), *Staphylococcus saprophyticus* (n=1). **Organism could not be identified at the species level by MALDI or according to best match by Kmer database. ***If the frequency of non-Enterobacterales was less than 10, then the respective species were considered as ‘others’.

**Supplementary Table 6. The associations of prevalent ARGs with** ***bla*_NDM-5_-positive isolates compared to *bla*_NDM-5_-negative isolates.**

| ARGs | Presence of ARG, n (%) | | Adjusted *p* value* | OR | 95% CI |
| --- | --- | --- | --- | --- | --- |
|  | ***bla*_NDM-5_-positive isolates (n=97)** | ***bla*_NDM-5_-negative isolates (n=539)** |  |  |  |
| *aadA2* | 77 (79.4) | 110 (20.4) | <0.0001 | 15.015 | 8.797-25.628 |
| *APH(3'')-Ib* | 48 (49.5) | 152 (28.2) | <0.0001 | 2.494 | 1.606-3.873 |
| *APH(3'')-Ia* | 15 (15.5) | 46 (8.5) | 0.035 | 1.960 | 1.046-3.673 |
| *APH(6)-Id* | 47 (48.5) | 181 (33.6) | 0.005 | 1.859 | 1.202-2.877 |
| *armA* | 28 (28.9) | 86 (16) | 0.003 | 2.138 | 1.302-3.510 |
| *catI* | 26 (26.8) | 55 (10.2) | <0.0001 | 3.223 | 1.899-5.468 |
| *bla*_CTX-M-15_ | 84 (86.6) | 332 (61.6) | <0.0001 | 4.029 | 2.190-7.410 |
| *bla*_CMY-59_ | 39 (40.2) | 78 (14.5) | <0.0001 | 3.974 | 2.480-6.368 |
| *bla*_OXA-1_ | 61 (62.9) | 195 (36.2) | <0.0001 | 2.989 | 1.910-4.678 |
| *bla*_TEM-1_ | 87 (89.7) | 224 (41.6) | <0.0001 | 12.234 | 6.220-24.065 |
| *bla*_VEB-5_ | 20 (20.6) | 11 (2) | <0.0001 | 12.468 | 5.752-27.024 |
| *dfrA12* | 77 (79.4) | 98 (18.2) | <0.0001 | 17.325 | 10.112-29.682 |
| *sul1* | 87 (89.7) | 244 (45.3) | <0.0001 | 10.518 | 5.350-20.681 |
| *sul2* | 55 (56.7) | 189 (35.1) | <0.0001 | 2.425 | 1.564-3.761 |
| *qnrS1* | 38 (39.2) | 107 (19.9) | <0.0001 | 2.600 | 1.643-4.116 |
| *rmtB* | 73 (75.3) | 19 (3.5) | <0.0001 | 83.246 | 43.466-159.430 |
| *ermB* | 48 (49.5) | 52 (9.6) | <0.0001 | 9.174 | 5.620-14.977 |
| *mphA* | 85 (87.6) | 185 (34.3) | <0.0001 | 13.554 | 7.219-25.450 |
| *mphB* | 39 (40.2) | 151 (28) | 0.017 | 1.728 | 1.105-2.702 |

Values in parentheses indicate column percentage. We calculated the linkage of any ARGs with *bla*_NDM-5_ whether the frequency of ARG was above 20. The associations between *bla*_NDM-5_, with other ARGs were mentioned in the table if *p* values were <0.05. As chromosomal class A β-lactamase *bla*_SHV_, *bla*_OKP_ and *bla*_LEN_ genes have been directly associated with KPI (*K. pneumoniae*), KPII (*K. quasipneumoniae*), and KPIII (*K. variicola*) respectively, the associations between these genes (*bla*_SHV_, *bla*_OKP_ and *bla*_LEN_) and *bla*_NDM-5_ were not assessed. The associations of *bla*_ampC_, *bla*_ampH_ and *fosA* with *bla*_NDM-5_ were not assessed as we found the presence of *bla*_ampC_ and *bla*_ampH_ only in *E. coli* and *fosA* among the species of *Klebsiella*. The number of efflux pumps was not included in this analysis. One strain of *Proteus* spp. (due to low quality sequence data), *Salmonella* spp. (n=5), and *L. adecarboxylata* (n=1) were not included in the analysis. *The *p* values were adjusted by the BH procedure.

**Supplementary table 7.** **The associations of prevalent ARGs with *bla*_NDM-1_-positive isolates compared to *bla*_NDM-1_-negative isolates.**

| ARGs | Presence of ARG, n (%) | | Adjusted *p* value* | OR | 95% CI |
| --- | --- | --- | --- | --- | --- |
|  | ***bla*_NDM-1_-positive isolates (n=62)** | ***bla*_NDM-1_-negative isolates (n=574)** |  |  |  |
| *AAC(2')-Ia* | 6 (9.7) | 12 (2.1) | 0.003 | 5.018 | 1.814-13.884 |
| *aadA2* | 25 (40.3) | 162 (28.2) | 0.051 | 1.718 | 1.002-2.946 |
| *APH(3')-Ia* | 19 (30.6) | 42 (7.3) | <0.0001 | 5.597 | 2.997-10.453 |
| *APH(3')-VI* | 16 (25.8) | 3 (0.5) | <0.0001 | 66.203 | 18.607-235.545 |
| *armA* | 24 (38.7) | 90 (15.7) | <0.0001 | 3.396 | 1.943-5.936 |
| *arr-2* | 20 (32.3) | 52 (9.1) | <0.0001 | 4.780 | 2.613-8.745 |
| *arr-3* | 6 (9.7) | 17 (3) | 0.015 | 3.511 | 1.330-9.264 |
| *catB3* | 6 (9.7) | 17 (3) | 0.014 | 3.511 | 1.330-9.264 |
| *dfrA14* | 16 (25.8) | 83 (14.5) | 0.024 | 2.058 | 1.113-3.804 |
| *mphE* | 30 (48.4) | 127 (22.1) | <0.0001 | 3.300 | 1.931-5.638 |
| *bla*_OXA-1_ | 39 (62.9) | 217 (37.8) | 0.0004 | 2.790 | 1.622-4.798 |
| *bla*_OXA-9_ | 9 (14.5) | 20 (3.5) | 0.0005 | 4.704 | 2.040-10.848 |
| *SAT-1* | 9 (14.5) | 46 (8) | 0.088 | 1.949 | 0.904-4.202 |
| *qnrA1* | 6 (9.7) | 16 (2.8) | 0.012 | 3.737 | 1.406-9.933 |
| *qnrB17* | 18 (29) | 82 (14.3) | 0.005 | 2.455 | 1.352-4.455 |
| *qnrD1* | 8 (12.9) | 29 (5.1) | 0.019 | 2.784 | 1.213-6.392 |
| *rmtF* | 14 (22.6) | 27 (4.7) | <0.0001 | 5.909 | 2.906-12.016 |
| *sul1* | 48 (77.4) | 283 (49.3) | 0.0001 | 3.525 | 1.901-6.537 |

Values in parentheses indicate column percentage. We calculated the linkage of any ARGs with *bla*_NDM-1_ whether the frequency of ARG was above 20. The associations between *bla*_NDM-1_, with other ARGs were mentioned in the table if *p* values were <0.05. As chromosomal class A β-lactamase *bla*_SHV_, *bla*_OKP_ and *bla*_LEN_ genes have been directly associated with KPI (*K. pneumoniae*), KPII (*K. quasipneumoniae*), and KPIII (*K. variicola*) respectively, the associations between these genes (*bla*_SHV_, *bla*_OKP_ and *bla*_LEN_) and *bla*_NDM-1_ were not assessed. The associations of *bla*_ampC_, *bla*_ampH_ and *fosA* with *bla*_NDM-5_ were not assessed as we found the presence of *bla*_ampC_ and *bla*_ampH_ only in *E. coli* and *fosA* among the species of *Klebsiella*. The number of efflux pumps was not included in this analysis. One strain of *Proteus* spp. (due to low quality sequence data), *Salmonella* spp. (n=5), and *L. adecarboxylata* (n=1) were not included in the analysis. *The *p* values were adjusted by the BH procedure.

**Supplementary Table 8.** **The associations of prevalent ARGs with *bla*_OXA-181_-positive isolates compared to *bla*_OXA-181_-negative isolates.**

| ARGs | Presence of ARG, n (%) | | Adjusted *p* value* | OR | 95% CI |
| --- | --- | --- | --- | --- | --- |
|  | ***bla*_OXA-181_-positive isolates (n=24)** | ***bla*_OXA-181_-negative isolates (n=612)** |  |  |  |
| *aadA2* | 12 (50) | 175 (28.6) | 0.028 | 0.4 | 0.177-0.908 |
| *rmtF* | 10 (41.7) | 31 (5.1) | <0.0001 | 0.075 | 0.031-0.182 |
| *bla*_CMY-59_ | 10 (41.7) | 107 (17.5) | 0.013 | 0.297 | 0.128-0.686 |
| *bla*_CTX-M-15_ | 23 (95.8) | 393 (64.2) | 0.019 | 0.078 | 0.010-0.582 |
| *arr-2* | 13 (54.2) | 59 (9.6) | <0.0001 | 0.09 | 0.039-0.211 |
| *arr-3* | 3 (12.5) | 20 (3.3) | 0.031 | 0.236 | 0.065-0.858 |
| *dfrA12* | 13 (54.2) | 162 (26.5) | 0.010 | 0.305 | 0.134-0.694 |
| *mphA* | 16 (66.7) | 254 (41.5) | 0.024 | 0.355 | 0.150-0.842 |
| *ermB* | 9 (37.5) | 91 (14.9) | 0.008 | 0.291 | 0.124-0.685 |

Values in parentheses indicate column percentage. We calculated the linkage of any ARGs with *bla*_OXA-181_ whether the frequency of ARG was above 20. The associations between *bla*_OXA-181_, and other ARGs were mentioned in the table if *p* values were <0.05. As chromosomal class A β-lactamase *bla*_SHV_, *bla*_OKP_ and *bla*_LEN_ genes have been directly associated with KPI (*K. pneumoniae*), KPII (*K. quasipneumoniae*), and KPIII (*K. variicola*) respectively, the associations between these genes (*bla*_SHV_, *bla*_OKP_ and *bla*_LEN_) and *bla*_OXA-181_ were not assessed. The associations of *bla*_ampC_, *bla*_ampH_ and *fosA* with *bla*_OXA-181_ were not assessed as we found the presence of *bla*_ampC_ and *bla*_ampH_ only in *E. coli* and *fosA* among the species of *Klebsiella*. The number of efflux pumps was not included in this analysis. One strain of *Proteus* spp. (due to low quality sequence data), *Salmonella* spp. (n=5), and *L. adecarboxylata* (n=1) were not included in the analysis. *The *p* values were adjusted by the BH procedure.

**Supplementary Table 9. Range of carbapenems’ MIC of OXA-232 and OXA-181 producing Enterobacterales.**

| Carbapenemase variants | Carbapenems | Range of MIC (mg/l) |
| --- | --- | --- |
| *bla*_NDM-1_+*bla*_OXA-232_ (n=3) | IPM | 8 to 16 |
|  | MEM | 16 to 32 |
| *bla*_NDM-5_+*bla*_OXA-232_ (n=3) | IPM | 16 to 32 |
|  | MEM | 64 to 128 |
| *bla*_OXA-232_ (n=11) | IPM | 1 to 8 |
|  | MEM | 16 |
| *bla*_OXA-232_ (phenotypically characterized as CSE) (n=9) | IPM | 0.5 to 2 |
|  | MEM | 0.5 to 2 |
| *bla*_KPC-2_+*bla*_OXA-181_ (n=5) | IPM | 16 to 32 |
|  | MEM | 32 to 64 |
| *bla*_NDM-5_+*bla*_OXA-181_ (n=3) | IPM | 2 to 8 |
|  | MEM | 8 to 32 |
| *bla*_NDM-7_+*bla*_OXA-181_ (n=1) | IPM | 256 |
|  | MEM | ≥256 |
| *bla*_OXA-181_ (n=14) | IPM | 4 to 32 |
|  | MEM | 0.06 to 64 |
| *bla*_OXA-181_ (phenotypically characterized as CSE) (n=1) | IPM | 0.125 |
|  | MEM | 0.06 |

IPM, imipenem; MEM, meropenem.

**Supplementary Table 10. The frequency of patients with at least one effective antimicrobial stratified based on patients’ outcome.**

| Clustering of patients based on usage of antimicrobials* | Number of patients with at least one effective antimicrobial tested in this study | | |
| --- | --- | --- | --- |
|  | DAMA | All-cause in-hospital 30-day mortality | Discharged alive/in-hospital mortality after 30 days |
| AMC (n=1) | - | - | 0 |
| AMC+CAZ (n=1) | 1 | - | - |
| AMC+MTZ (n=1) | - | - | 0 |
| AMK (n=1) | - | - | 0 |
| AMK+AZM+MTZ (n=2) | 1 | 1 | - |
| AMK+CAZ (n=6) | 1 | 1 | 1 |
| AMK+CAZ+CLT+LVX (n=1) | - | - | 0 |
| AMK+CAZ+CRO+MTZ (n=1) | - | - | 1 |
| AMK+CAZ+FLU+MEM+VAN (n=1) | - | - | 1 |
| AMK+CAZ+MEM (n=1) | - | - | 0 |
| AMK+CFX+CRO+LVX (n=1) | - | - | 0 |
| AMK+CIP (n=2) | - | - | 0 |
| AMK+CIP+FLU+LVX+LZD+MTZ (n=1) | - | - | 0 |
| AMK+CIP+IPM (n=1) | - | 0 | - |
| AMK+CIP+MEM (n=1) | - | - | 1 |
| AMK+CIP+MTZ (n=2) | - | - | 2 |
| AMK+CLI+LVX+MEM (n=1) | - | - | 1 |
| AMK+CLT+LVX (n=1) | - | - | 0 |
| AMK+CRO (n=25) | 2 | 1 | 6 |
| AMK+CRO+CIP+MTZ (n=6) | - | 0 | 0 |
| AMK+CRO+FLU (n=6) | - | 1 | 4 |
| AMK+CRO+FLU+CLI (n=1) | - | - | 0 |
| AMK+CRO+LVX (n=15) | - | 2 | 6 |
| AMK+CRO+MEM (n=4) | - | 0 | 0 |
| AMK+CRO+MEM+MTZ (n=2) | 0 | 1 | - |
| AMK+CRO+MTZ (n=9) | 1 | 1 | 4 |
| AMK+CRO+MXF (n=1) | - | 0 | - |
| AMK+CST (n=2) | - | 1 | 1 |
| AMK+CST+CRO (n=2) | - | - | 1 |
| AMK+MEM (n=4) | - | 1 | 3 |
| AMK+MEM+MTZ (n=3) | - | 1 | - |
| AZM+CAZ+FLU (n=1) | - | - | 0 |
| AZM+CLI+PEN G (n=1) | - | 0 | - |
| AZM+CRO (n=1) | - | - | 0 |
| AZM+CRO+MEM (n=1) | - | 0 | - |
| AZM+IPM+LVX (n=1) | 1 | - | - |
| AZM+LVX (n=1) | - | - | 0 |
| CAZ+CLI (n=2) | - | - | 0 |
| CAZ+CRO (n=1) | - | - | 0 |
| CAZ+CRO+FLU (n=1) | - | - | 0 |
| CAZ+CRO+LVX (n=1) | - | - | 0 |
| CAZ+STP (n=1) | - | - | 0 |
| CFX (n=6) | - | 0 | 0 |
| CFX+CIP+CRO (n=1) | - | - | 0 |
| CFX+CIP+CRO+MTZ (n=2) | - | - | 0 |
| CFX+CIP+MTZ (n=3) | 0 | - | 1 |
| CFX+CRO+FLU (n=1) | - | - | 0 |
| CFX+CRO+LVX (n=2) | 1 | - | 0 |
| CFX+CRO+MTZ (n=4) | - | - | 3 |
| CFX+FLU (n=8) | - | - | 1 |
| CFX+IPM (n=1) | - | - | 0 |
| CFX+LVX (n=1) | - | - | 0 |
| CIP (n=23) | 0 | 0 | 3 |
| CIP+CLI+MEM (n=1) | - | - | 1 |
| CIP+CRO (n=3) | - | - | 2 |
| CIP+FLU (n=1) | - | - | 0 |
| CIP+MEM (n=2) | - | 1 | 1 |
| CIP+MEM+MTZ (n=1) | - | - | 1 |
| CIP+MTZ (n=40) | 0 | 0 | 5 |
| CLI (n=1) | - | - | 0 |
| CLI+CRO (n=20) | 1 | 0 | 2 |
| CLI+CRO+CST (n=1) | - | - | 0 |
| CLI+CRO+LVX (n=6) | - | 1 | 1 |
| CLI+CRO+MEM (n=5) | - | 0 | 2 |
| CLI+CRO+MTZ (n=1) | - | 0 | - |
| CLI+CST (n=1) | - | - | 1 |
| CLI+CST+MEM+MTZ (n=1) | - | 1 | - |
| CLI+DXC+LVX+MEM (n=1) | - | - | 0 |
| CLI+FLU (n=1) | - | - | 0 |
| CLI+FLU+MEM (n=2) | - | 0 | 1 |
| CLI+IPM (n=1) | 1 | - | - |
| CLI+LVX (n=1) | - | - | 0 |
| CLI+MEM+MXF (n=1) | - | 0 | - |
| CLI+MEM+MXF+TZP (n=1) | - | 0 | - |
| CRO (n=25) | 0 | 0 | 0 |
| CRO+CIP+FLU (n=2) | - | - | 0 |
| CRO+CIP+MTZ (n=31) | 0 | 1 | 4 |
| CRO+CLI+MEM+MTZ (n=1) | - | 0 | - |
| CRO+CLI+MEM+VAN (n=1) | - | - | 1 |
| CRO+CST+IPM+LVX (n=2) | - | 1 | 1 |
| CRO+CST+LVX (n=2) | - | - | 2 |
| CRO+CST+LZD (n=1) | - | 0 | - |
| CRO+CST+MEM (n=1) | - | - | 1 |
| CRO+FLU (n=43) | 2 | 1 | 5 |
| CRO+FLU+LVX (n=2) | - | 0 | 0 |
| CRO+FLU+MEM (n=4) | - | 2 | 1 |
| CRO+FLU+MEM+MTZ (n=2) | 0 | 0 | - |
| CRO+FLU+MTZ (n=10) | 0 | - | 1 |
| CRO+GEN (n=7) | 1 | 1 | 3 |
| CRO+GEN+MTZ (n=1) | 1 | - | - |
| CRO+IPM (n=1) | 0 | - | - |
| CRO+LVX (n=34) | - | 3 | 3 |
| CRO+LVX+MEM (n=1) | - | 0 | - |
| CRO+MEM+MTZ (n=2) | - | 1 | 0 |
| CRO+MEM+MTZ+VAN (n=1) | - | 0 | - |
| CRO+MTZ (n=17) | 0 | - | 4 |
| CRO+MTZ+LVX (n=1) | - | 0 | - |
| CRO+VAN (n=12) | - | 0 | 2 |
| CST+FLU (n=1) | - | - | 1 |
| CST+LVX+MEM (n=1) | - | 1 | - |
| CST+MEM (n=1) | - | - | 1 |
| CST+MEM+MTZ (n=1) | - | - | 1 |
| CXM (n=2) | 1 | - | 1 |
| FLU (n=1) | - | 0 | - |
| FLU+IPM (n=1) | - | - | 1 |
| FLU+LVX (n=1) | - | - | 0 |
| FLU+MEM (n=3) | - | 0 | 3 |
| FLU+MEM+MTZ+VAN (n=1) | - | - | 1 |
| FLU+MTZ (n=2) | - | - | 0 |
| LVX+MTZ (n=1) | - | - | 0 |
| MEM (n=5) | 1 | 1 | 2 |
| MEM+MTZ (n=5) | - | 0 | 2 |
| MEM+MTZ+LVX (n=1) | - | 1 | - |
| MEM+MTZ+VAN (n=1) | - | - | 0 |
| MEM+MXF (n=1) | - | 0 | - |
| MEM+VAN (n=4) | 1 | - | 2 |
| MTZ (n=3) | 0 | - | 0 |
| TET (n=1) | - | - | 0 |
| No antibiotic (n=31) | 0 | - | 0 |
| Total (n=534) | 17 | 27 | 100 |

AMC, amoxicillin-clavulanic acid; AMK, Amikacin; AZM, azithromycin; CAZ, ceftazidime; CFX, cefixime; CXM, cefuroxime; CIP, ciprofloxacin; CLI, clindamycin; CLT, clarithromycin; CRO, ceftriaxone; CST, colistin; CTX, cefotaxime; DXC, doxycycline; FEP, cefepime; FLU, flucloxacillin; FOF, fosfomycin; GEN, gentamicin; IPM, imipenem; LVX, levofloxacin; LZD, linezolid; MEM, meropenem; MTZ, metronidazole; MXF, moxifloxacin; PEN G, Penicillin G; STP, streptomycin; SXT, sulfamethoxazole-trimethoprim; TET, tetracycline; TZP, piperacillin-tazobactam; VAN, vancomycin; n, number of patients. *Total number of patients received respective antimicrobials. Antibiotics in the first column are highlighted whether the susceptibility patterns were tested in this study.

**Supplementary Table 11. Distribution of carbapenem-resistant *E. coli* among major clonal types compared to carbapenem-sensitive *E. coli*.**

| Sequence types | CRE (n=54) | CSE (n=172) | Adjusted *p* value* | OR | 95% CI |
| --- | --- | --- | --- | --- | --- |
| ST131 | 2 (3∙7) | 21 (12∙2) | 0∙179 | 3∙616 | 0∙820-15∙952 |
| ST405 | 9 (16∙7) | 12 (7∙0) | 0∙094 | 0∙375 | 0∙149-0∙946 |
| ST410 | 3 (5∙6) | 18 (10∙5) | 0∙477 | 1∙987 | 0∙562-7∙023 |
| ST648 | 9 (16∙7) | 12 (7∙0) | 0∙125 | 0∙375 | 0∙149-0∙946 |
| ST167 | 11 (20∙4) | 7 (4∙1) | 0∙004 | 0∙166 | 0∙061-0∙453 |
| ST101 | 4 (7∙4) | 8 (4∙7) | 0∙543 | 0∙610 | 0∙176-2∙110 |
| ST38 | 2 (3∙7) | 7 (4∙1) | 0∙904 | 1∙103 | 0∙222-5∙475 |
| ST448 | 5 (9∙3) | 1 (0∙6) | 0∙049 | 0∙057 | 0∙007-0∙502 |
| ST2659 | 2 (3∙7) | 3 (1∙7) | 0∙577 | 0∙462 | 0∙075-2∙837 |
| ST617 | 1 (1∙9) | 4 (2∙3) | 0∙929 | 1∙262 | 0∙138-11∙537 |
| ST224 | 0 (0) | 4 (2∙3) | - | - | - |
| ST226 | 0 (0) | 4 (2∙3) | - | - | - |
| ST8346 | 3 (5∙6) | 0 (0) | - | - | - |

Values in parentheses indicate column percentage. *The *p* values were adjusted by the BH procedure.

**Supplementary Table 12. Distribution of *E. coli* among different phylogroups and their corresponding STs (n=226).**

| Phylogroups | Prevalence [n (%)] | Corresponding STs (n) |
| --- | --- | --- |
| A | 53 (23.5) | ST167 (18), ST617 (5), ST10 (4), ST226 (4), ST361 (2), ST4204 (2), ST4981 (2), ST10820 (1), ST1139 (1), ST1286 (1), ST1421 (1), ST181 (1), ST206 (1), ST2253 (1), ST2509 (1), ST3107 (1), ST450 (1), ST46 (1), ST6083 (1), ST609 (1), ST656 (1), ST939 (1), ST9660 (1) |
| D | 49 (21.7) | ST405 (21), ST38 (9), ST101 (5), ST2659 (5), novel ST (2), ST394 (2), ST1011 (1), ST2076 (1), ST5954 (1), ST68 (1), ST69 (1) |
| B1 | 36 (15.9) | ST101 (7), ST448 (6), ST224 (4), ST8346 (3), ST2083 (2), ST424 (2), ST10821 (1), ST1326 (1), ST155 (1), ST156 (1), ST162 (1), ST1891 (1), ST2161 (1), ST2179 (1), ST443 (1), ST602 (1) |
| B2 | 34 (15) | ST131 (23), ST73 (4), ST12 (2), novel ST (1), ST1193 (1), ST421 (1), ST827 (1), ST8395 (1) |
| C | 25 (11.1) | ST410 (21), ST2851 (2), ST3221 (1), ST90 (1) |
| F | 25 (11.1) | ST648 (21), ST2011 (2), ST354 (1), ST8881 (1) |
| E | 2 (0.9) | ST9663 (1), ST2083 (1) |
| G | 2 (0.9) | ST117 (2) |

n=number of isolates.

**Supplementary Table 13. The overall information obtained from time-scaled tress in this study.**

| STs/putative transmission clusters | Median MRCA including both DMCH’s and NCBI strains (95% CI) | Range of median MRCA for DMCH’s subclades only | Median clock rate (SNP/genome/year) | Figure |
| --- | --- | --- | --- | --- |
| *E. coli* ST167 (including ST10, ST1702, and novel allele) | 1902 (1835-1953) | - | 2∙80 | Figure 4 |
| *E. coli* ST167 | 1978 | 2004-2015 | - | Figure 4 |
| *E. coli* ST448 (including ST2083, ST1702, and novel allele) | 1924 (1901-1943) | 2008-2017 | 3∙40 | Figure 4 |
| *E. coli* ST8346 | 2008 (1998-2013) | 2010-2013 | 4∙65 | Figure 4 |
| *E. coli* ST405 (including ST5954) | 1922 (1893-1941) | 1990-1999 | 1∙20 | Figure 4 |
| *E. coli* ST648 (including ST2011, ST6870, and ST9666) | 1999 (1992-2004) | 2006-2013 | 3∙50 | Figure 4 |
| *K. pneumoniae* ST15 (including a novel allele) | 1985 (1944-2004) | 1998-2016 | 1∙30 | Figure 5 |
| *K. pneumoniae* ST16 | 1932 (1919-1944) | 2014-2017 | 3∙15 | Figure 5 |
| EC1 | - | 2015 | - | Figure 4 |
| EC2 | - | 2016 | - | Figure 4 |
| EC5 | - | 2016 | - | Figure 4 |
| EC3 | - | 2013 | - | Figure 4 |
| EC4 | - | 2015 | - | Figure 4 |
| EC6 | - | 2015 | - | Figure 4 |
| KP6 | - | 2016 | - | Figure 5 |
| KP7 | - | 2015 | - | Figure 5 |
| KP8 | - | 2014 | - | Figure 5 |

**Supplementary Table 14.** **Distribution of carbapenem resistant *K. pneumoniae* among major clonal types compared to CSE.**

| Sequence types | CRE (n=119) | CSE (n=102) | Adjusted *p* value* | OR | 95% CI |
| --- | --- | --- | --- | --- | --- |
| ST23 | 24 (20·2) | 11 (10·8) | 0·201 | 0·478 | 0·222-1·033 |
| ST15 | 12 (10·1) | 17 (16·7) | 0·380 | 1·783 | 0·808-3·937 |
| ST231 | 15 (12·6) | 3 (2·9) | 0·160 | 0·210 | 0·059-0·748 |
| ST11 | 14 (11·8) | 3 (2·8) | 0·114 | 0·227 | 0·063-0·815 |
| ST152 | 6 (5) | 6 (5·9) | 0·784 | 1·177 | 0·368-3·769 |
| ST147 | 7 (5·9) | 4 (3·9) | 0·563 | 0·653 | 0·186-2·298 |
| ST14 | 6 (5) | 3 (2·9) | 0·623 | 0·571 | 0·139-2·342 |
| ST395 | 6 (5) | 3 (2·9) | 0·545 | 0·571 | 0·139-2·342 |
| ST16 | 8 (6·7) | 0 (0) | - | - | - |
| ST307 | 2 (1·7) | 5 (4·9) | 0·385 | 3·015 | 0·572-15·557 |
| ST515 | 6 (5) | 0 (0) | - | - | - |
| ST101 | 0 (0) | 6 (5·9) | - | - | - |
| ST43 | 4 (3∙4) | 0 (0) | - | - | - |

Values in parentheses indicate column percentage. *The *p* values were adjusted by the BH procedure.

**Supplementary Table 15. The prevalence of different capsular K loci among *K. pneumoniae* isolated in this study along with corresponding STs.**

| Capsular types | Prevalence [n (%)] | Corresponding STs (n) |
| --- | --- | --- |
| KL1 | 35 (15.8) | ST23 (35) |
| KL51 | 23 (10.4) | ST231 (18), ST16 (3), ST252 (1), ST15 (1) |
| KL112 | 21 (9.5) | ST15 (20), ST395 (1) |
| KL24 | 18 (8.1) | ST11 (14), ST15 (3), ST20 (1) |
| KL149 | 12 (5.4) | ST152 (12) |
| KL64 | 9 (4.1) | ST14 (5), ST147 (4) |
| KL17 | 8 (3.6) | ST101 (5), ST474 (2), ST870 (1) |
| KL48 | 8 (3.6) | ST16 (5), ST15 (2), novel ST (1) |
| KL10 | 6 (2.7) | ST147 (3), ST395 (2), ST1128 (1) |
| KL102 | 6 (2.7) | ST307 (6) |
| KL108 | 6 (2.7) | ST395 (6) |
| KL146 | 6 (2.7) | ST515 (6) |
| KL30 | 5 (2.3) | ST43 (4), ST1145 (1) |
| KL62 | 4 (1.8) | ST48 (1), ST39 (1), ST45 (1), ST664 (1) |
| KL124 | 3 (1.4) | ST48 (3) |
| KL2 | 3 (1.4) | ST14 (3) |
| KL3 | 3 (1.4) | ST490 (3) |
| KL8 | 3 (1.4) | ST15 (3) |
| KL105 | 2 (0.9) | ST11 (2) |
| KL113 | 2 (0∙9) | ST111 (1), ST234 (1) |
| KL114 | 2 (0∙9) | ST219 (2) |
| KL122 | 2 (0∙9) | ST11 (1), ST120 (1) |
| KL128 | 2 (0∙9) | ST147 (2) |
| KL14 | 2 (0∙9) | ST147 (1), ST656 (1) |
| KL16 | 2 (0∙9) | ST14 (1), ST215 (1) |
| KL19 | 2 (0∙9) | ST29 (7), ST17 (1) |
| KL5 | 2 (0∙9) | ST2050 (1), ST45 (1) |
| KL55 | 2 (0∙9) | ST17 (1), ST20 (1) |
| KL9 | 2 (0∙9) | ST584 (1), novel ST (1) |
| KL106 | 1 (0.5) | Novel ST (1) |
| KL107 | 1 (0.5) | ST3003 (1) |
| KL117 | 1 (0.5) | ST34 (1) |
| KL12 | 1 (0.5) | ST37 (1) |
| KL123 | 1 (0.5) | ST572 (1) |
| KL125 | 1 (0.5) | ST378 (1) |
| KL126 | 1 (0.5) | ST1586 (1) |
| KL148 | 1 (0∙5) | ST1787 (1) |
| KL15 | 1 (0∙5) | ST2616 (1) |
| KL155 | 1 (0∙5) | ST307 (1) |
| KL167 | 1 (0∙5) | ST3630 (1) |
| KL20 | 1 (0∙5) | ST420 (1) |
| KL21 | 1 (0∙5) | ST540 (1) |
| KL25 | 1 (0∙5) | ST17 (1) |
| KL34 | 1 (0∙5) | ST500 (1) |
| KL39 | 1 (0∙5) | ST327 (1) |
| KL52 | 1 (0∙5) | ST38 (1) |
| KL54 | 1 (0∙5) | ST711 (1) |
| KL74 | 1 (0∙5) | ST273 (1) |
| KL81 | 1 (0∙5) | ST147 (1) |

n=number of isolates.

**Supplementary Table 16. Brief description of microbiology capacity of the major MCHs of Bangladesh (Scoping exercise done by Timothy R. Walsh and Refath Farzana on May 2019).**

|  | CMCH | DMCH | KMCH | MMCH | RMCH | RpMCH | SOMCH |
| --- | --- | --- | --- | --- | --- | --- | --- |
| # core staff | 7 | 2 | 2 | 2 | 2 | 2 | 5 |
| # rotating staff | 14 | 14 | 5 | 15 | 15 | 11 | 14 |
| # beds | 1326 | 2600 | 500 | 1000 | 1200 | 1000 | 500 |
| # in-patients | 3000 | 7000 | 980 | 3000 | 3000 | 2100 | 2300 |
| # out-patients | 5500 | 5000 | 3500 | 5000 | 2500 | 4000 | 4500 |
| Automated blood culture | - | √√√ | √* | - | - | - | √√ |
| Sputum culture | - | √ | - | √ | √ | - | √ |
| Wound swab culture | - | √ | - | √ | √ | √ | √ |
| Urine culture | - | √ | - | √ | √ | √ | √ |
| ⊗AST – disc | √ | √√√ | √ | √ | √√√ | √ | √√√ |
| AST – other | - | - | - | - | - |  | - |
| Digital reporting | - | - | - | - | - | - | - |

CMCH, Chittagong Medical College Hospital; DMCH, Dhaka Medical College Hospital; KMCH, Khulna Medical College Hospital; MMCH, Mymensingh Medical College Hospital; RpMCH, Rangpur Medical College Hospital; RMCH, Rajshahi Medical College Hospital; SOMCH, Sylhet M.A.G. Osmani Medical College Hospital. *Dysfunctional. ⊗Number of ticks reflects the degree of understanding and correct methodology.

**Supplementary Table 17. Genome attributes of the isolates retrieved from NCBI.**

| Biosample | Species | ST Warwick | Application |
| --- | --- | --- | --- |
| SAMN13951922 | *E. coli* | ST656 | Figure 4A |
| SAMN08993917 | *E. coli* | ST117 | Figure 4A |
| SAMN05194390 | *E. coli* | ST405 | Figure 4A |
| SAMN07618120 | *E. coli* | ST929 | Figure 4A |
| SAMN07618121 | *E. coli* | ST349 | Figure 4A |
| SAMN07618127 | *E. coli* | ST539 | Figure 4A |
| SAMN03263950 | *E. coli* | ST10 | Figure 4A |
| SAMN08161275 | *E. coli* | ST1312 | Figure 4A |
| SAMN08161312 | *E. coli* | ST94 | Figure 4A |
| SAMN04158294 | *E. coli* | ST405 | Figure 4A |
| SAMN12569951 | *E. coli* | ST410 | Figure 4A |
| SAMEA2272277 | *E. coli* | ST414 | Figure 4A |
| SAMN06219550 | *E. coli* | ST4542 | Figure 4A |
| SAMN06219548 | *E. coli* | ST602 | Figure 4A |
| SAMN07792806 | *E. coli* | ST793 | Figure 4A |
| SAMN07807402 | *E. coli* | ST88 | Figure 4A |
| SAMN13242665 | *E. coli* | ST641 | Figure 4A |
| SAMN08579578 | *E. coli* | ST5350 | Figure 4A |
| SAMN08579581 | *E. coli* | ST967 | Figure 4A |
| SAMN08579566 | *E. coli* | ST205 | Figure 4A |
| SAMN08579558 | *E. coli* | ST655 | Figure 4A |
| SAMN11266496 | *E. coli* | ST583 | Figure 4A |
| SAMEA104093909 | *E. coli* | ST8217 | Figure 4A |
| SAMN08579587 | *E. coli* | ST646 | Figure 4A |
| SAMN07260764 | *E. coli* | ST635 | Figure 4A |
| SAMN03252409 | *E. coli* | ST38 | Figure 4A |
| SAMN03252413 | *E. coli* | ST295 | Figure 4A |
| SAMN08668598 | *E. coli* | ST5030 | Figure 4A |
| SAMN03612246 | *E. coli* | ST99 | Figure 4A |
| SAMN06928086 | *E. coli* | ST69 | Figure 4A |
| SAMN08663441 | *E. coli* | ST448 | Figure 4A |
| SAMN08663435 | *E. coli* | ST7786 | Figure 4A |
| SAMN10250173 | *E. coli* | ST206 | Figure 4A |
| SAMN10531954 | *E. coli* | ST448 | Figure 4A |
| SAMN06198937 | *E. coli* | ST167 | Figure 4A |
| SAMN06198938 | *E. coli* | ST156 | Figure 4A |
| SAMN08040561 | *E. coli* | ST764 | Figure 4A |
| SAMN03252419 | *E. coli* | ST642 | Figure 4A |
| SAMN03252421 | *E. coli* | ST90 | Figure 4A |
| SAMN03252423 | *E. coli* | ST58 | Figure 4A |
| SAMN03252425 | *E. coli* | ST224 | Figure 4A |
| SAMN08382661 | *E. coli* | ST131 | Figure 4A |
| SAMN11283108 | *E. coli* | ST167 | Figure 4A |
| SAMN02949644 | *E. coli* | ST847 | Figure 4A |
| SAMN07759721 | *E. coli* | ST2705 | Figure 4A |
| SAMN05511150 | *E. coli* | ST648 | Figure 4A |
| SAMN05511168 | *E. coli* | ST44 | Figure 4A |
| SAMEA4916090 | *E. coli* | ST8346 | Figure 4A |
| SAMN09710898 | *E. coli* | ST80 | Figure 4A |
| SAMN07503734 | *E. coli* | ST443 | Figure 4A |
| SAMN04625459 | *E. coli* | ST182 | Figure 4A |
| SAMN03996288 | *E. coli* | ST538 | Figure 4A |
| SAMN10163231 | *E. coli* | ST636 | Figure 4A |
| SAMN05210898 | *E. coli* | ST795 | Figure 4A |
| SAMN08450093 | *E. coli* | ST93 | Figure 4A |
| SAMN03963234 | *E. coli* | ST56 | Figure 4A |
| SAMD00168435 | *E. coli* | ST542 | Figure 4A |
| SAMN03252430 | *E. coli* | ST46 | Figure 4A |
| SAMN03252431 | *E. coli* | ST101 | Figure 4A |
| SAMN02604037 | *E. coli* | ST46 | Figure 4A |
| SAMEA3138234 | *E. coli* | ST62 | Figure 4A |
| SAMN07765387 | *E. coli* | ST648 | Figure 4A |
| SAMN04481707 | *E. coli* | ST131 | Figure 4A |
| SAMN00016779 | *E. coli* | ST1079 | Figure 4A |
| SAMN10219252 | *E. coli* | ST2161 | Figure 4A |
| SAMN11087649 | *E. coli* | ST515 | Figure 4A |
| SAMN09704974 | *E. coli* | ST73 | Figure 4A |
| SAMD00059754 | *E. coli* | ST167 | Figure 4A |
| SAMN03252442 | *E. coli* | ST5082 | Figure 4A |
| SAMN09462202 | *E. coli* | ST1193 | Figure 4A |
| SAMN03960341 | *E. coli* | ST607 | Figure 4A |
| SAMN03287565 | *E. coli* | ST131 | Figure 4A |
| SAMN06029894 | *E. coli* | ST617 | Figure 4A |
| SAMN06029895 | *E. coli* | ST617 | Figure 4A |
| SAMN06029893 | *E. coli* | ST617 | Figure 4A |
| SAMD00056131 | *E. coli* | ST457 | Figure 4A |
| SAMEA5128442 | *E. coli* | ST648 | Figure 4A |
| SAMN04448503 | *E. coli* | ST6140 | Figure 4A |
| SAMEA3529258 | *E. coli* | ST57 | Figure 4A |
| SAMEA3368339 | *E. coli* | ST8740 | Figure 4A |
| SAMN02673556 | *E. coli* | ST1011 | Figure 4A |
| SAMN07594018 | *E. coli* | ST446 | Figure 4A |
| SAMD00179457 | *E. coli* | ST4577 | Figure 4A |
| SAMN08165042 | *E. coli* | ST937 | Figure 4A |
| SAMN03081526 | *E. coli* | ST6130 | Figure 4A |
| SAMN03252453 | *E. coli* | ST48 | Figure 4A |
| SAMN03252454 | *E. coli* | ST442 | Figure 4A |
| SAMN03252457 | *E. coli* | ST517 | Figure 4A |
| SAMN06909178 | *E. coli* | ST410 | Figure 4A |
| SAMD00061087 | *E. coli* | ST156 | Figure 4A |
| SAMN02604066 | *E. coli* | ST354 | Figure 4A |
| SAMN02666696 | *E. coli* | ST6131 | Figure 4A |
| SAMN02647163 | *E. coli* | ST540 | Figure 4A |
| SAMN02800875 | *E. coli* | ST648 | Figure 4A |
| SAMN12214763 | *E. coli* | ST162 | Figure 4A |
| SAMN00017915 | *E. coli* | ST643 | Figure 4A |
| SAMN03294311 | *E. coli* | ST10 | Figure 4A |
| SAMN03963241 | *E. coli* | ST155 | Figure 4A |
| SAMN10956393 | *E. coli* | ST167 | Figure 4A |
| SAMN06909153 | *E. coli* | ST405 | Figure 4A |
| SAMN10691120 | *E. coli* | ST354 | Figure 4A |
| SAMN07974445 | *E. coli* | ST964 | Figure 4A |
| SAMN12233489 | *E. coli* | ST761 | Figure 4A |
| SAMN06218054 | *E. coli* | ST167 | Figure 5A |
| SAMN06284178 | *E. coli* | ST167 | Figure 5A |
| SAMN04361562 | *E. coli* | Unknown ST | Figure 5A |
| SAMN04157977 | *E. coli* | ST167 | Figure 5A |
| SAMN04157978 | *E. coli* | ST167 | Figure 5A |
| SAMN04157979 | *E. coli* | ST167 | Figure 5A |
| SAMN04157980 | *E. coli* | ST167 | Figure 5A |
| SAMN07344983 | *E. coli* | ST167 | Figure 5A |
| SAMN04157981 | *E. coli* | ST167 | Figure 5A |
| SAMN07344982 | *E. coli* | ST167 | Figure 5A |
| SAMN09985619 | *E. coli* | ST167 | Figure 5A |
| SAMD00059754 | *E. coli* | ST167 | Figure 5A |
| SAMN08281024 | *E. coli* | ST167 | Figure 5A |
| SAMN03220397 | *E. coli* | ST167 | Figure 5A |
| SAMN06909181 | *E. coli* | ST167 | Figure 5A |
| SAMN05977366 | *E. coli* | ST448 | Supplementary Figure 1 |
| SAMN06311279 | *E. coli* | ST448 | Supplementary Figure 1 |
| SAMN06311280 | *E. coli* | ST448 | Supplementary Figure 1 |
| SAMN08637775 | *E. coli* | Unknown ST | Supplementary Figure 1 |
| SAMN10531472 | *E. coli* | ST448 | Supplementary Figure 1 |
| SAMEA4672938 | *E. coli* | ST2083 | Supplementary Figure 1 |
| SAMEA4916063 | *E. coli* | ST448 | Supplementary Figure 1 |
| SAMEA4916089 | *E. coli* | ST448 | Supplementary Figure 1 |
| SAMN06973353 | *E. coli* | ST448 | Supplementary Figure 1 |
| SAMN04992597 | *E. coli* | ST448 | Supplementary Figure 1 |
| SAMN10105866 | *E. coli* | ST448 | Supplementary Figure 1 |
| SAMN12822948 | *E. coli* | ST205 | Supplementary Figure 1 |
| SAMN10531954 | *E. coli* | ST448 | Supplementary Figure 1 |
| SAMN06909173 | *E. coli* | ST448 | Supplementary Figure 1 |
| SAMN10620088 | *E. coli* | ST448 | Supplementary Figure 1 |
| SAMN08637787 | *E. coli* | ST8346 | Supplementary Figure 2 |
| SAMEA4916096 | *E. coli* | ST8346 | Supplementary Figure 2 |
| SAMEA4916090 | *E. coli* | ST8346 | Supplementary Figure 2 |
| SAMN13829989 | *E. coli* | ST405 | Supplementary Figure 3 |
| SAMN06311269 | *E. coli* | ST405 | Supplementary Figure 3 |
| SAMN06311274 | *E. coli* | ST405 | Supplementary Figure 3 |
| SAMN06311283 | *E. coli* | ST405 | Supplementary Figure 3 |
| SAMN08637779 | *E. coli* | ST405 | Supplementary Figure 3 |
| SAMN08637780 | *E. coli* | ST405 | Supplementary Figure 3 |
| SAMN08637778 | *E. coli* | ST405 | Supplementary Figure 3 |
| SAMN02801869 | *E. coli* | ST405 | Supplementary Figure 3 |
| SAMN02801870 | *E. coli* | ST405 | Supplementary Figure 3 |
| SAMN08637781 | *E. coli* | ST405 | Supplementary Figure 3 |
| SAMN10249152 | *E. coli* | ST405 | Supplementary Figure 3 |
| SAMN10248954 | *E. coli* | ST405 | Supplementary Figure 3 |
| SAMN04046665 | *E. coli* | ST405 | Supplementary Figure 3 |
| SAMD00076990 | *E. coli* | ST405 | Supplementary Figure 3 |
| SAMN06973348 | *E. coli* | ST405 | Supplementary Figure 3 |
| SAMN07312492 | *E. coli* | ST405 | Supplementary Figure 3 |
| SAMEA4643524 | *E. coli* | ST405 | Supplementary Figure 3 |
| SAMN11232786 | *E. coli* | ST405 | Supplementary Figure 3 |
| SAMN05604797 | *E. coli* | ST405 | Supplementary Figure 3 |
| SAMN03145051 | *E. coli* | ST405 | Supplementary Figure 3 |
| SAMN05786429 | *E. coli* | ST405 | Supplementary Figure 3 |
| SAMN07760941 | *E. coli* | ST405 | Supplementary Figure 3 |
| SAMN11233046 | *E. coli* | ST405 | Supplementary Figure 3 |
| SAMN11233061 | *E. coli* | ST405 | Supplementary Figure 3 |
| SAMN11233063 | *E. coli* | ST405 | Supplementary Figure 3 |
| SAMN11233068 | *E. coli* | ST405 | Supplementary Figure 3 |
| SAMN11233092 | *E. coli* | ST405 | Supplementary Figure 3 |
| SAMN11233094 | *E. coli* | ST405 | Supplementary Figure 3 |
| SAMN06924979 | *E. coli* | ST405 | Supplementary Figure 3 |
| SAMN06311264 | *E. coli* | ST648 | Supplementary Figure 4 |
| SAMN06806389 | *E. coli* | ST648 | Supplementary Figure 4 |
| SAMN02709589 | *E. coli* | ST648 | Supplementary Figure 4 |
| SAMN08103372 | *E. coli* | ST648 | Supplementary Figure 4 |
| SAMN08103377 | *E. coli* | ST648 | Supplementary Figure 4 |
| SAMN08519241 | *E. coli* | ST648 | Supplementary Figure 4 |
| SAMN08519242 | *E. coli* | ST648 | Supplementary Figure 4 |
| SAMN08519249 | *E. coli* | ST648 | Supplementary Figure 4 |
| SAMN03922986 | *E. coli* | ST648 | Supplementary Figure 4 |
| SAMD00052661 | *E. coli* | ST648 | Supplementary Figure 4 |
| SAMN05440277 | *E. coli* | ST648 | Supplementary Figure 4 |
| SAMN05440276 | *E. coli* | ST648 | Supplementary Figure 4 |
| SAMN11232909 | *E. coli* | ST648 | Supplementary Figure 4 |
| SAMN05511150 | *E. coli* | ST648 | Supplementary Figure 4 |
| SAMN11233027 | *E. coli* | ST648 | Supplementary Figure 4 |
| SAMN05774083 | *K. pneumoniae* | ST983 | Figure 4B |
| SAMN05231873 | *K. pneumoniae* | ST37 | Figure 4B |
| SAMN08391417 | *K. pneumoniae* | ST307 | Figure 4B |
| SAMN07638733 | *K. pneumoniae* | ST54 | Figure 4B |
| SAMN02603641 | *K. pneumoniae* | ST23 | Figure 4B |
| SAMN03097203 | *K. pneumoniae* | ST65 | Figure 4B |
| SAMN10343201 | *K. pneumoniae* | ST1685 | Figure 4B |
| SAMN08366843 | *K. pneumoniae* | ST1265 | Figure 4B |
| SAMN08366844 | *K. pneumoniae* | ST23 | Figure 4B |
| SAMN09476115 | *K. pneumoniae* | ST29 | Figure 4B |
| SAMN07187263 | *K. pneumoniae* | ST15 | Figure 4B |
| SAMN03076168 | *K. pneumoniae* | ST48 | Figure 4B |
| SAMN03076171 | *K. pneumoniae* | ST395 | Figure 4B |
| SAMN08212048 | *K. pneumoniae* | ST147 | Figure 4B |
| SAMN08222595 | *K. pneumoniae* | ST101 | Figure 4B |
| SAMN03196972 | *K. pneumoniae* | ST846 | Figure 4B |
| SAMN03076172 | *K. pneumoniae* | ST11 | Figure 4B |
| SAMN03197141 | *K. pneumoniae* | ST327 | Figure 4B |
| SAMN03197209 | *K. pneumoniae* | ST70 | Figure 4B |
| SAMN04868736 | *K. pneumoniae* | ST11 | Figure 4B |
| SAMN12264830 | *K. pneumoniae* | ST16 | Figure 4B |
| SAMN10678732 | *K. pneumoniae* | ST86 | Figure 4B |
| SAMN04868740 | *K. pneumoniae* | ST258 | Figure 4B |
| SAMN03197492 | *K. pneumoniae* | ST34 | Figure 4B |
| SAMN08222601 | *K. pneumoniae* | ST111 | Figure 4B |
| SAMN03197549 | *K. pneumoniae* | ST60 | Figure 4B |
| SAMN07760932 | *K. pneumoniae* | ST147 | Figure 4B |
| SAMN03197962 | *K. pneumoniae* | ST234 | Figure 4B |
| SAMN03197968 | *K. pneumoniae* | ST528 | Figure 4B |
| SAMN03455989 | *K. pneumoniae* | ST45 | Figure 4B |
| SAMN10790857 | *K. pneumoniae* | ST23 | Figure 4B |
| SAMN04014907 | *K. pneumoniae* | ST14 | Figure 4B |
| SAMN04014916 | *K. pneumoniae* | ST16 | Figure 4B |
| SAMN04014981 | *K. pneumoniae* | ST34 | Figure 4B |
| SAMN07291519 | *K. pneumoniae* | ST15 | Figure 4B |
| SAMN10432823 | *K. pneumoniae* | ST231 | Figure 4B |
| SAMEA3141919 | *K. pneumoniae* | ST91 | Figure 4B |
| SAMN10910564 | *K. pneumoniae* | ST15 | Figure 4B |
| SAMN02138655 | *K. pneumoniae* | ST186 | Figure 4B |
| SAMN02138664 | *K. pneumoniae* | ST15 | Figure 4B |
| SAMN02138667 | *K. pneumoniae* | ST661 | Figure 4B |
| SAMN02356586 | *K. pneumoniae* | ST540 | Figure 4B |
| SAMN02581290 | *K. pneumoniae* | ST17 | Figure 4B |
| SAMN03280412 | *K. pneumoniae* | ST23 | Figure 4B |
| SAMN03280413 | *K. pneumoniae* | ST23 | Figure 4B |
| SAMN02138593 | *K. pneumoniae* | ST133 | Figure 4B |
| SAMN02581256 | *K. pneumoniae* | ST592 | Figure 4B |
| SAMN02581302 | *K. pneumoniae* | ST17 | Figure 4B |
| SAMN03280382 | *K. pneumoniae* | ST16 | Figure 4B |
| SAMN12289282 | *K. pneumoniae* | ST12 | Figure 4B |
| SAMN10537194 | *K. pneumoniae* | ST11 | Figure 4B |
| SAMN03733725 | *K. pneumoniae* | ST941 | Figure 4B |
| SAMN02581304 | *K. pneumoniae* | ST1199 | Figure 4B |
| SAMN03280255 | *K. pneumoniae* | ST15 | Figure 4B |
| SAMN03280300 | *K. pneumoniae* | ST158 | Figure 4B |
| SAMN03280347 | *K. pneumoniae* | ST2121 | Figure 4B |
| SAMN02581368 | *K. pneumoniae* | ST14 | Figure 4B |
| SAMN09811763 | *K. pneumoniae* | ST726 | Figure 4B |
| SAMN02603941 | *K. pneumoniae* | ST38 | Figure 4B |
| SAMD00171892 | *K. pneumoniae* | ST668 | Figure 4B |
| SAMEA4364586 | *K. pneumoniae* | ST15 | Figure 5B |
| SAMEA4364670 | *K. pneumoniae* | ST15 | Figure 5B |
| SAMN02138664 | *K. pneumoniae* | ST15 | Figure 5B |
| SAMN10963497 | *K. pneumoniae* | ST15 | Figure 5B |
| SAMEA3721057 | *K. pneumoniae* | ST15 | Figure 5B |
| SAMEA3512047 | *K. pneumoniae* | ST15 | Figure 5B |
| SAMEA3649536 | *K. pneumoniae* | ST15 | Figure 5B |
| SAMEA3721053 | *K. pneumoniae* | ST15 | Figure 5B |
| SAMEA3721059 | *K. pneumoniae* | ST15 | Figure 5B |
| SAMEA3515136 | *K. pneumoniae* | ST15 | Figure 5B |
| SAMEA3538544 | *K. pneumoniae* | ST15 | Figure 5B |
| SAMEA3729663 | *K. pneumoniae* | ST15 | Figure 5B |
| SAMN10600498 | *K. pneumoniae* | ST15 | Figure 5B |
| SAMEA2273810 | *K. pneumoniae* | ST15 | Figure 5B |
| SAMN03281077 | *K. pneumoniae* | ST15 | Figure 5B |
| SAMEA4364585 | *K. pneumoniae* | ST16 | Supplementary Figure 5 |
| SAMEA4394736 | *K. pneumoniae* | ST16 | Supplementary Figure 5 |
| SAMEA23996668 | *K. pneumoniae* | ST16 | Supplementary Figure 5 |
| SAMEA23998168 | *K. pneumoniae* | ST16 | Supplementary Figure 5 |
| SAMN07173931 | *K. pneumoniae* | ST16 | Supplementary Figure 5 |
| SAMN07173932 | *K. pneumoniae* | ST16 | Supplementary Figure 5 |
| SAMEA104569902 | *K. pneumoniae* | ST16 | Supplementary Figure 5 |
| SAMEA3499992 | *K. pneumoniae* | ST16 | Supplementary Figure 5 |
| SAMN10592664 | *K. pneumoniae* | ST16 | Supplementary Figure 5 |
| SAMEA2273664 | *K. pneumoniae* | ST16 | Supplementary Figure 5 |
| SAMEA2273689 | *K. pneumoniae* | ST16 | Supplementary Figure 5 |
| SAMEA2273709 | *K. pneumoniae* | ST16 | Supplementary Figure 5 |
| SAMEA2273723 | *K. pneumoniae* | ST16 | Supplementary Figure 5 |
| SAMEA2273726 | *K. pneumoniae* | ST16 | Supplementary Figure 5 |
| SAMN07450698 | *K. pneumoniae* | ST16 | Supplementary Figure 5 |
| SAMEA3345126 | *K. pneumoniae* | ST16 | Supplementary Figure 5 |
| SAMEA3531804 | *K. pneumoniae* | ST16 | Supplementary Figure 5 |
| SAMEA3531868 | *K. pneumoniae* | ST16 | Supplementary Figure 5 |
| SAMN04008891 | *K. pneumoniae* | ST16 | Supplementary Figure 5 |

**Supplementary Table 18. Accession numbers of complete circular plasmids carried *bla*_NDM_ and *bla*_OXA_.**

| Local id | Degignation1* | Degignation2** | Accession no |
| --- | --- | --- | --- |
| b193b-ndm-1-p | C_N1_2 | N1G1 | CP095567 |
| dm12b-ndm-1-p | C_N1_2 | N1G1 | CP095597 |
| dm186b-ndm-1-p | C_N1_2 | N1G1 | CP095599 |
| dm330-ndm-1-p | C_N1_2 | N1G1 | CP095609 |
| dm478b-ndm-1-p | C_N1_2 | N1G1 | CP095620 |
| bpro_s102a-ndm-1-p | C_N1_3 | N1G2 | CP095591 |
| bpro_s12-ndm-1-p | C_N1_3 | N1G2 | CP095592 |
| bpro_s46a-ndm-1-p | C_N1_3 | N1G2 | CP095593 |
| dmeb_c388-ndm-1-p | FII_N1_1 | N1G2 | CP095673 |
| dmpro_s193a-ndm-1-p | C_N1_3 | N1G2 | CP095676 |
| dmpro_s749a-ndm-1-plasmid | un_N1_1 | N1G2 | CP095677 |
| dmeb_c17-ndm-1-p | HI1B_N1_1 | N1G3 | CP095671 |
| dmeb_c801b-ndm-1-p | HI1A_N1_1 | N1G3 | CP095674 |
| b56-ndm-1-p | FIB(pQil)_N1_2 | N1G4 | CP095585 |
| b82-ndm-1-p | FIB(pQil)_N1_2 | N1G4 | CP096183 |
| dm285b-ndm-1-p | FIA_N1_1 | N1G4 | CP095604 |
| dm356b-ndm-1-p | FIA_N1_1 | N1G4 | CP095612 |
| dm614a-ndm-1-p | FIB_N1_1 | N1G4 | CP095635 |
| dm883b-ndm-1-p | FIB&FII_N1_2 | N1G4 | CP095661 |
| dm147a-ndm-1-p | FIB(pQil)_N1_3 | N1G5 | CP095598 |
| dm654a-ndm-1-p | FIB(pQil)_N1_3 | N1G5 | CP095636 |
| dm129b-ndm-1-p | FIB(pQil)_N1_1 | N1G6 | CP095596 |
| dm602b-ndm-1-p | FIB&C_N1_1 | N1G6 | CP095570 |
| dmsm701b-ndm-1-p | un_N1_2 | N1G6 | CP095679 |
| kv-ndm-1-p | FIB(pQil)_N1_1 | N1G6 | CP095680 |
| b127b-ndm-1-p | FIB&HI1B_N1_1 | N1G7 | CP095577 |
| b141b-ndm-1-p | FIB&HI1B_N1_1 | N1G7 | CP095578 |
| dm570b-ndm-1-p | FIB&HI1B_N1_1 | N1G7 | CP095631 |
| dm600b-ndm-1-p | FIB&HI1B_N1_1 | N1G7 | CP095633 |
| dm770b-ndm-1-p | FIB&HI1B_N1_1 | N1G7 | CP095649 |
| dm849a-ndm-1-p | C_N1_1 | N1G7 | CP095658 |
| dm898b-ndm-1-p | HI2_N1_1 | N1G7 | CP095662 |
| dm978b-ndm-1-p | FIB&HI1B_N1_1 | N1G7 | CP095667 |
| dm228-ndm-1-p | R_N1_2 | N1G8 | CP095602 |
| dm740a-ndm-1-p | FIB&FII_N1_1 | N1G8 | CP095646 |
| dm796c-ndm-1-p | R_N1_1 | N1G8 | CP095653 |
| dmeb_c292b-ndm-1-p | X3_N1_1 | N1G8 | CP095672 |
| b199b-ndm-5-p | FII_N5_5 | N5G1 | CP095580 |
| b57b-ndm-5-p | FII_N5_2 | N5G1 | CP095586 |
| dm09-ndm-5-p | FII_N5_2 | N5G1 | CP095594 |
| dm336b-ndm-5-p | FII_N5_2 | N5G1 | CP095569 |
| dm363b-ndm-5-p | FIB&FII_N5_1 | N5G1 | CP095613 |
| dm372b-ndm-5-p | FII_N5_2 | N5G1 | CP095615 |
| dm382b-ndm-5-p | FII_N5_2 | N5G1 | CP095616 |
| dm514-ndm-5-p | FII_N5_1 | N5G1 | CP095625 |
| dm566-ndm-5-p | FIA_N5_3 | N5G1 | CP095630 |
| dm682b-ndm-5-p | FIA_N5_2 | N5G1 | CP095639 |
| dm693b-ndm-5-p | FIB&FII_N5_1 | N5G1 | CP095641 |
| dm702b-ndm-5-p | FIA_N5_3 | N5G1 | CP095643 |
| dm708c-ndm-5-p | FIA_N5_3 | N5G1 | CP095644 |
| dm728-ndm-5-p | FIA_N5_3 | N5G1 | CP095645 |
| dm832c-ndm-5-p | FII_N5_2 | N5G1 | CP095656 |
| dm882b-ndm-5-p | FIA_N5_4 | N5G1 | CP095660 |
| dm900-ndm-5-p | FII_N5_2 | N5G1 | CP095664 |
| dm277c-ndm-5-p | R_N5_1 | N5G1 | CP096176 |
| b119-ndm-5-p | FII_N5_3 | N5G2 | CP095576 |
| b148b-ndm-5-p | FII_N5_3 | N5G2 | CP095579 |
| b217b-ndm-5-p | FII_N5_3 | N5G2 | CP095582 |
| b46b-ndm-5-p | FIB(pQil)&FII_N5_1 | N5G2 | CP095584 |
| dm106c-ndm-5-p | FII_N5_3 | N5G2 | CP095595 |
| dm218b-ndm-5-p | FII_N5_3 | N5G2 | CP095601 |
| dm314c-ndm-5-p | FII_N5_3 | N5G2 | CP095605 |
| dm320b-ndm-5-p | FII_N5_3 | N5G2 | CP095606 |
| dm328b-ndm-5-p | FII_N5_3 | N5G2 | CP095608 |
| dm338b-ndm-5-p | FII_N5_3 | N5G2 | CP095610 |
| dm353b-ndm-5-p | FII_N5_3 | N5G2 | CP095611 |
| dm424b-ndm-5-p | FII_N5_3 | N5G2 | CP095618 |
| dm428b-ndm-p | FII_N5_3 | N5G2 | CP095619 |
| dm478c-ndm-5-p | FII_N5_3 | N5G2 | CP095621 |
| dm485b-ndm-5-p | FII_N5_3 | N5G2 | CP095622 |
| dm496c-ndm-5-p | FII_N5_3 | N5G2 | CP095623 |
| dm509b-ndm-5-p | FII_N5_3 | N5G2 | CP095624 |
| dm522a-ndm-5-p | FII_N5_3 | N5G2 | CP095626 |
| dm533-ndm-5-p | FII_N5_3 | N5G2 | CP095628 |
| dm599b-ndm-5-p | FII_N5_3 | N5G2 | CP095632 |
| dm604b-ndm-5-p | FII_N5_3 | N5G2 | CP095634 |
| dm651b-ndm-5-p | FIB(pQil)_N5_1 | N5G2 | CP095571 |
| dm730a-ndm-5-p | FIB(pQil)_N5_1 | N5G2 | CP096174 |
| dm760b-ndm-5-p | FII_N5_3 | N5G2 | CP095648 |
| dm778b-ndm-5-p | FII_N5_3 | N5G2 | CP095650 |
| dm780b-ndm-5-p | FIB(pQil)_N5_1 | N5G2 | CP095652 |
| dm815-ndm-5-p | FII_N5_3 | N5G2 | CP095655 |
| dm884b-ndm-5-p | FII_N5_3 | N5G2 | CP096181 |
| dm899b-ndm-5-p | FII_N5_3 | N5G2 | CP095663 |
| dm930b-ndm-5-p | FII_N5_3 | N5G2 | CP095666 |
| dmcr749c-ndm-5-p | FII_N5_3 | N5G2 | CP095669 |
| dmcs955-ndm-5-p | FII_N5_3 | N5G2 | CP095670 |
| beb_c250b-copy1-ndm-5-p | FII_N5_3 | N5G2 | CP095681 |
| dm664b-ndm-5-copy1-p | FII_N5_3 | N5G2 | CP095682 |
| b156-ndm-5-copy2-p | FII_N5_3 | N5G2 | CP095683 |
| b35c-ndm-5-p | FIA_N5_6 | N5G3 | CP095583 |
| dm321b-ndm-5-p | FIA_N5_1 | N5G3 | CP095607 |
| dm654b-ndm-5-p | FII_N5_4 | N5G3 | CP095637 |
| b73b-ndm-5-p | X3_N5_1 | N5G4 | CP095587 |
| b75c-ndm-5-p | X3_N5_1 | N5G4 | CP095568 |
| beb_c20b-ndm-5-p | X3_N5_1 | N5G4 | CP095589 |
| beb_c2b-ndm-5-p | X3_N5_1 | N5G4 | CP095590 |
| dm420b-ndm-5-p | X3_N5_1 | N5G4 | CP095617 |
| dm692-ndm-5-p | X3_N5_1 | N5G4 | CP095640 |
| dm694-ndm-5-p | X3_N5_1 | N5G4 | CP095642 |
| beb_c250b-copy2-ndm-5-p | X3_N5_1 | N5G4 | CP095573 |
| dm664b-ndm-5-copy3-p | X3_N5_1 | N5G4 | CP095685 |
| dm655-ndm-5-p | FIA_N5_5 | N5G5 | CP095638 |
| dm664b-ndm-5-copy2-p | FIB(pQil)_N5_2 | N5G6 | CP095574 |
| dm193b-ndm-5-p | R_N5_2 | N5G7 | CP095600 |
| dm749b-ndm-5-p | R_N5_2 | N5G7 | CP095647 |
| dm803b-ndm-5-p | R_N5_2 | N5G7 | CP095654 |
| dm874-ndm-5-p | FIB_N5_1 | N5G8 | CP095659 |
| dm925c-ndm-5-p | FIB_N5_1 | N5G8 | CP095665 |
| b78b-ndm-5 | FII&C_N5_1 | N5G9 | CP095588 |
| b156b-ndm-5-copy1-p | C_N5_1 | N5G9 | CP095572 |
| 730a-copy-1-OXA-232 | FIB(pQil)_O232_1 | - | CP096173 |
| 683b-OXA-232 | ColKP3_O232_1 | - | CP095575 |
| b207b-m-OXA-232 | ColKP3_O232_1 | - | CP095581 |
| b82-m-OXA-232 | ColKP3_O232_1 | - | CP096182 |
| dm264b-m-OXA-181 | A/C2_O181_1 | - | CP095603 |
| 277c-OXA-181 | X3_O181_1 | - | CP096177 |
| dm365c-ndm-7-p | X3_N7_1 | - | CP095614 |
| dm378A-ndm-7-p | X3_N7_1 | - | CP096179 |
| dm378A-m-OXA-181 | FIC(FII)_O181_1 | - | CP096178 |
| dm525c-ndm-7-p | X3_N7_1 | - | CP095627 |
| dm546c-ndm-4-p | FIA_N4_1 | - | CP095629 |
| 651b-OXA-232 | ColKP3_O232_1 | - | CP095571 |
| 730a-copy-2-OXA-232 | ColKP3_O232_1 | - | CP096175 |
| dm732-ndm-4-p | FIA_N4_1 | - | CP095684 |
| dm778c-ndm-4-p | FIA_N4_1 | - | CP095651 |
| 843-OXA-232 | ColKP3_O232_1 | - | CP095657 |
| dm884b-m-OXA-181 | A/C2_O181_1 | - | CP096180 |
| dmcf952-ndm-7-p | X3_N7_1 | - | CP095668 |
| dmeb_c853-ndm-7-p | X3_N7_1 | - | CP095675 |
| dmsm540-ndm-7-p | X3_N7_1 | - | CP095678 |

* Assigned in this study based on resistance patterns, Inc types, and plasmid size (please see Table 5). **Assigned in this study based on genetic environment immediate to carbapenem resistant gene (please see Figure 8).
