# Supplementary material for: Genomic Insights Into the Mechanism of Carbapenem Resistance Dissemination in Enterobacterales From a Tertiary Public Heath Setting in South Asia
Source: Clin Infect Dis. 2022 Apr 27;76(1):119–33. doi: 10.1093/cid/ciac287 (PMC9825829; doi:10.1093/cid/ciac287)
Supplement: ciac287_Supplementary_Data [file ciac287_supplementary_data.zip › 05_Supplementary method_clean_04032022.docx]

**Ethical Approval Certificate**


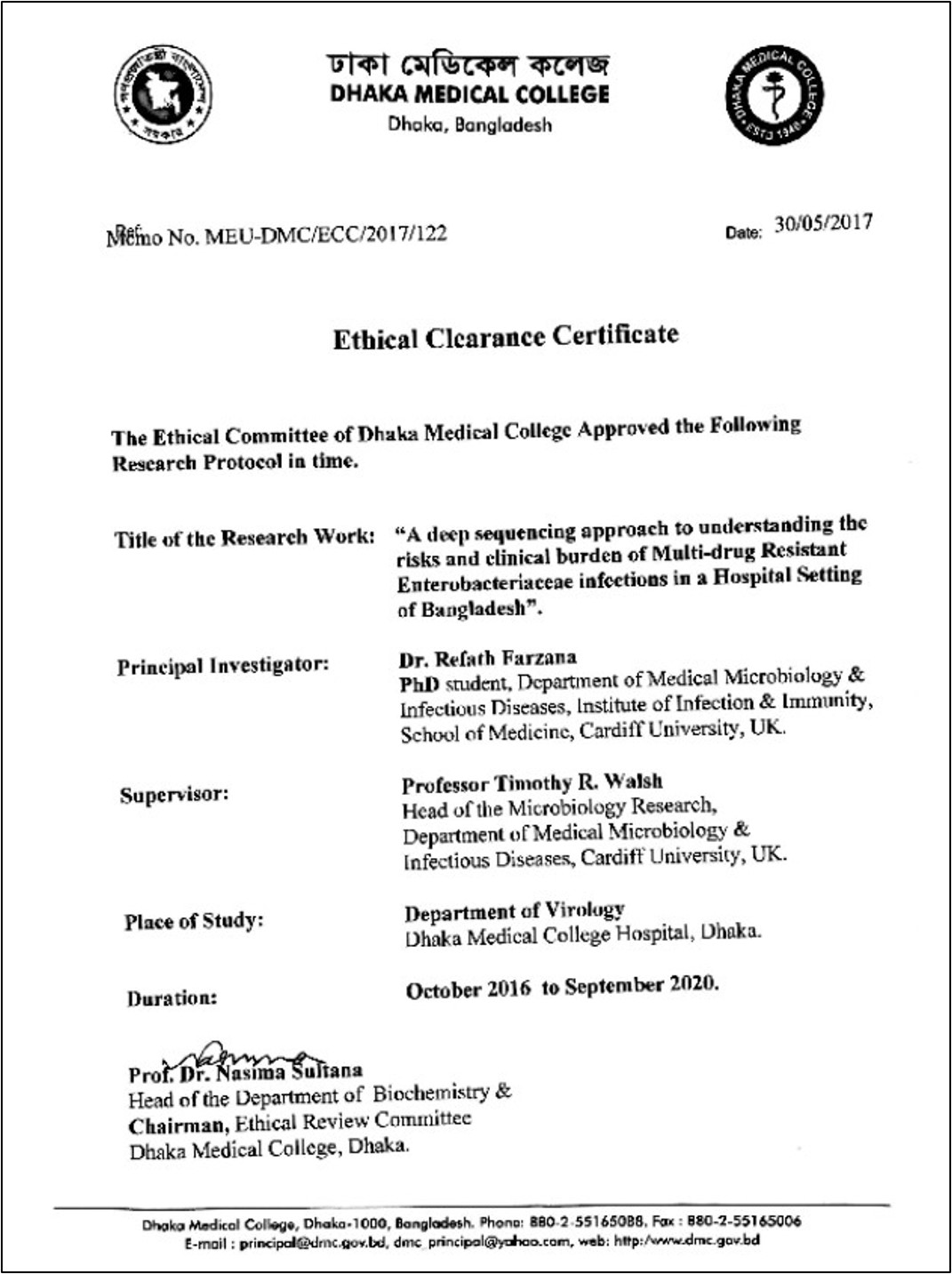


**Participants’ particulars.**

Patient data was anonymized and protected by encryption and passwords to protect patients in accordance with the Helsinki Declaration [1]. Patients’ name, age, sex, locality, family member, family income, clinical symptoms or reason for hospitalization, ward, type of specimen, outcome (discharge alive, DAMA, or death), date of admission, date of sample collection, date of outcome, and ongoing antibiotics used during hospitalisation were collected. Per capita monthly income (total monthly income of the family/total members of family) was used as parameter to determine the socio-economic condition of the patient in this study [2].

Per capita monthly income (total monthly income of the family/total members of family) was used as a parameter to determine the socio-economic condition of the patients in this study [2]. To calculate the original income scale range of each socio-economic class in Bangladesh in 2017 and 2018, the income range (INR) according to the modified proposed classification of the month of December 2004 was converted to Bangladeshi Taka (BDT) (1 INR is equivalent to about 1.25 BDT in 2017, and 2018) and then multiplied by a conversion factor [3]. The Conversion Factor (CF) between 2004 and the current year was determined by the following formula:

CF between 2004 and 2017 in Bangladesh= Consumer Price Index (CPI) in 2017 / CPI in 2004

In Bangladesh, the average CPIs were 235 and 245 in 2017 and 2018, respectively. In 2004, the CPI was around 90 [4]. Accordingly, the conversion factor was 2.61 and 2.72 for 2017 and 2018. The modification is illustrated in the following table. Fractions in income were ignored.

**Assessment of socio-economic status**

| Social classes | Per capita monthly family income limit | | | |
| --- | --- | --- | --- | --- |
|  | BG Prasad’s  Classification of  1961 (INR) | Modified proposed classification of the month of December 2004 (INR) | Modified for 2017 Bangladesh (on (BDT) | Modified for 2018 in Bangladesh (on (BDT) |
| I. Upper high | 100 and above | 10000 and above | 32,625, and above | 34,000, and above |
| II. High | 50-99 | 5000-9999 | 16,312-32,624 | 17,000-33,996 |
| III. Upper middle | 30-49 | 3000-4999 | 9,787-16,311 | 10,200-16,996 |
| IV. Lower middle | 15-29 | 1500-2999 | 4,893-9,786 | 5,100-10,196 |
| V. Poor | Below 15 | 500-1499 | 1,631-4,892 | 1,700-5,096 |
| VI. Below poverty level (BPL) |  | Below 500 | Below 1,630 | 1,700 |

**Illumina MiSeq sequencing**

All Enteribacterales isolated were sequenced on the Illumina MiSeq platform (Illumina Inc., San Diego, CA). DNA libraries were prepared for paired end sequencing (2x301 cycles) using Nextera XT v2. Genomic DNA was extracted from overnight culture using the QIAcube (Qiagen, Hilden, Germany), and resulting gDNA was quantified using the Qubit 3.0 (Thermos Fisher Scientific, Waltham, USA). Quality control of raw reads included fastqc (v0.11.2) and adaptor trimming was performed using Trimgalore (v0.4.3). Reads were assembled into contigs using the de novo assembler SPAdes (v3.9.0) (.fasta), and were aligned to the original fastq reads using Burrows-Wheeler aligner (BWA) (v0.7.15). Any error was corrected using Pilon (v1.2). Assembly metrics were evaluated using Quast (v2.1). The *de novo* assemblies comprised multiple contigs (145-330) and were then annotated with Prokka (v1.12).

**MinION sequencing**

A set of CRE (n=125) was characterized by minION sequencing (Oxford Nanopore technologies, Oxford, UK) in this study. AMPure XP (Beckman Coulter, California, USA) was used to process gDNA to obtain a concentration 53.3 ng/µl. A DNA library was prepared by pooling all samples. 1 µl of RAD was added to the pooled DNA. A final mixture of 75 µl (34 µl sequencing buffer, 25.5 μl loading beads, 4.5 µl water and 11 µl DNA library) was loaded to the flow cell. The MinION device was connected to the MinKNOW GUI to obtain the reads. Demultiplexing of reads was performed using Porechop (0.2.3). Unicycler (0.4.4) was used to yield hybrid assemblies using both Illumina short reads and minION long reads.

**Databases used in this study**

Genomes from the National Center for Biotechnology Information (NCBI) were retrieved for three purposes: 1) constructing phylogenetic trees for each species to characterise population structure and identify clonal lineages, 2) to act as reference genomes for whole genome-based SNP alignments, and 3) phylogeographic analysis. The Biosample accessions of the isolates retrieved from NCBI in this study are provided in Supplementary Table 21. Phylogeographic analysis also included faecal isolates recovered in 2018 from DMCH (not published, but the genomes have been submitted to NCBI under BioProjects of PRJNA722682, PRJNA719593, and PRJNA714521). The Kmer database (v3.0.2) [available in Center for Genomic Epidemiology (CGE)] was deployed for species identification, Clermont phylotyping (v1.4.0) for *Escherichia coli* and Kaptive (v0.7.3) for *Klebsiella pneumoniae* capsular typing. The Comprehensive Antibiotic Resistance Database (CARD) and PlasmidFinder were deployed for antimicrobial resistance genes (ARGs) and plasmid replicon types with a cut off of ≥95% coverage and ≥95% identity, respectively using ABRicate (v0.9.7). Multilocus sequence types (MLST) were assigned based on seven loci MLST databases in CGE (v2.0.0), where appropriate.

**Phylogenetic analysis**

The annotated assemblies were used as input for Roary (v3.12.0) (a gene was considered as core if it was present in 99% of isolates and clustering of isolates based on presence of genes showing minimum blastp percentage identity of 95%) to generate core genome alignments for each species. Maximum likelihood (ML) phylogenetic trees based on the core genome alignments were created using RAxML-ng (v0.9.0.git-mpi) with a general time reversible (GTR) evolutionary model and gamma correction with iterations until bootstrapping converged with cut-off value of 3%. Visualisation of phylogenetic trees and incorporation of metadata were performed using iTOL (v5). Snippy (v4.4.5) was used to generate whole genome SNP-based alignments for each selected sequence type. Recombination removal was performed using Gubbins (v2.3.4) and pairwise SNP distance matrices were generated using pairsnp (v0.0.7). The ancestral sequence at each node including the root was inferred using pyjar and the pairwise SNP distance between the roots and each isolate was calculated using pairsnp (v0.0.7).

**Phylogeographic analysis**

Time-calibrated evolutionary analysis of the major clonal lineages associated with carbapenem resistance was performed using the BEAST package (v1.10.4) to estimate the date of the most recent common ancestor (MRCA). The following parameters were applied for Markov Chain Monte Carlo (MCMC) runs: 1) HKY substitution model (as default), Gamma plus invariant sites heterogeneity model with estimated base frequency of 4 relative rates of mutations across sites 2) four different models (strict clock and constant population size, strict clock and exponential growth, relaxed clock and constant population size, and relaxed clock and exponential growth) were assessed 3) MCMC chains were carried out for 10^8^ generations. Each MCMC run was performed in triplicate. The best fit model was selected by comparing marginal likelihood estimates for each model and selecting the one with the highest Bayes factor (BF) (Baele et al., 2013). Convergence of MCMC runs was assessed using Tracer (v1.7.1) and MCMC runs were only accepted if the effective sample size (ESS) for each parameter >200. Maximum clade credibility (MCC) trees were obtained from the tree’s posterior distributions, after a 10% burn-in, with Tree-Annotator (v1.10.4) from the BEAST package. The trees were visualized using FigTree (v1.4.4).

**Reference**

1. General Assembly of the World Medical Association. World Medical Association Declaration of Helsinki: ethical principles for medical research involving human subjects. J Am Coll Dent 2014, 81: 14-18.
2. Agarwal A. Social Classification: The Need to Update in the Present Scenario. Indian J Community Med 2008, 33: 50-51.
3. [Ghosh A](https://www.ncbi.nlm.nih.gov/pubmed/?term=Ghosh%20A%5BAuthor%5D&cauthor=true&cauthor_uid=20061591), [Ghosh T](https://www.ncbi.nlm.nih.gov/pubmed/?term=Ghosh%20T%5BAuthor%5D&cauthor=true&cauthor_uid=20061591). Modification of Kuppuswamys Socioeconomic Status Scale in Context to Nepal. [Indian Pediatr](https://www.ncbi.nlm.nih.gov/pubmed/20061591) 2009, 46: 1104-1105.
4. Trading Economics. Bangladesh Consumer Price Index (CPI). Available at: <https://tradingeconomics.com/bangladesh/consumer-price-index-cpi>. Accessed 15 August 2018.

**Titles of Supplementary Figures**

**Supplementary Figure 1. Time calibrated phylogenetic tree generated from of *E. coli* genomes belonged to ST448.**

**Figure legend.** Total number of isolates in this analysis was 45. Closely related isolates from other STs (ST2083, ST1702, and novel allele) identified by core-genome phylogeny and pair-wise SNPs count (if isolates were differed by ≤100 SNPs from any isolate of ST448) were included in this analysis. Putative transmission clades (0-10 SNPs differences) are highlighted by green. MRCA and clock rate are stated in Supplementary Table 13. Isolates retrieved from NCBI for the phylogenetic analysis in this figure are stated in Supplementary Table 17. PSU, paediatric surgery.

**Supplementary Figure 2. Time calibrated phylogenetic tree generated from of *E. coli* genomes belonged to ST8346.**

**Figure legend.** Total number of isolates in this analysis was 32. Putative transmission clades (0-10 SNPs differences) are highlighted by green. MRCA and clock rate are stated in Supplementary Table 13. Isolates retrieved from NCBI for the phylogenetic analysis in this figure are stated in Supplementary Table 17. PSU, paediatric surgery.

**Supplementary Figure 3. Time calibrated phylogenetic tree generated from of *E. coli* genomes belonged to ST405.**

**Figure legend.** Total number of isolates in this analysis was 77. Closely related isolates from other STs (ST5954) identified by core-genome phylogeny and pair-wise SNPs count (if isolates were differed by ≤100 SNPs from any isolate of ST405) were included in this analysis. Putative transmission clades (0-10 SNPs differences) are highlighted by green. MRCA and clock rate are stated in Supplementary Table 13. Isolates retrieved from NCBI for the phylogenetic analysis in this figure are stated in Supplementary Table 17. PSU, paediatric surgery.

**Supplementary Figure 4. Time calibrated phylogenetic tree generated from of *E. coli* genomes belonged to ST648.**

**Figure legend.** Total number of isolates in this analysis was 57. Closely related isolates from other STs (ST2011, ST6870, and ST9666) identified by core-genome phylogeny and pair-wise SNPs count (if isolates were differed by ≤100 SNPs from any isolate of ST648) were included in this analysis. Putative transmission clades (0-10 SNPs differences) are highlighted by green. MRCA and clock rate are stated in Supplementary Table 13. Isolates retrieved from NCBI for the phylogenetic analysis in this figure are stated in Supplementary Table 17. PSU, paediatric surgery.

**Supplementary Figure 5. Time calibrated phylogenetic tree generated from of *K. pneumoniae* genomes belonged to ST16.**

**Figure legend.** Total number of isolates in this analysis was 37. Putative transmission clades (0-10 SNPs differences) are highlighted by blue and green. Putative transmission clades (0-10 SNPs differences) are highlighted by blue, green and pink. MRCA and clock rate are stated in Supplementary Table 13. Isolates retrieved from NCBI for the phylogenetic analysis in this figure are stated in Supplementary Table 17.

**Supplementary Figure 6. Heatmap representing the percentage of ARGs present in different plasmid backgrounds coharbouring carbapenem resistant genes.**

**Figure legend.** NDM- or OXA-positive plasmids characterized in this study were included in this figure. Number of plasmids belonged to different Inc type were included with column ID. Values in the cells indicate row percentage.

**Supplementary Figure 7.** **Characterization of IncFII plasmids harbouring *bla*_NDM-5_ (FII_N5_2).**

**Figure legend. A.** Schematic layout of IncFII plasmid of group FII_N5_2 carrying *bla*_NDM-5_ identified in this study. Arrows represent the position and transcriptional direction of the open reading frames. Resistance genes are represented by red, genes for mobile elements by green, genes associated with conjugation in blue, replication-associated genes in pink, regulatory/accessory/hypothetical proteins in yellow. **B.** Colinear alignment of IncFII plasmids harbouring *bla*_NDM-5_ of group FII_N5_2 (n=7). One plasmid from each clonal type was selected for the alignment. Alignment was performed by Mauve using DNASTAR (v17.1). Accession numbers of specific plasmids’ sequences are stated in Supplementary Table 18. The layout of genetic context has been outlined using Geneious (v11.0.2).

**Supplementary Figure 8.** **Characterization of** **IncFII plasmids carrying *bla*_NDM-5_ (FII_N5_3).**

**Figure legend. A.** Schematic layout of IncFII plasmid of group FII_N5_3 carrying *bla*_NDM-5_ identified in this study. Arrows represent the position and transcriptional direction of the open reading frames. Resistance genes are represented by red, genes for mobile elements by green, genes associated with conjugation in blue, replication-associated genes in pink, regulatory/accessory/hypothetical proteins in yellow. **B.** Colinear alignment of IncFII plasmids harbouring *bla*_NDM-5_ of group FII_N5_3 (n=31). One plasmid from each clonal type was selected for the alignment. Alignment was performed by Mauve using DNASTAR (v17.1). Accession numbers of specific plasmids’ sequences are stated in Supplementary Table 18. The layout of genetic context has been outlined using Geneious (v11.0.2).

**Supplementary Figure 9. Characterization of IncX3 plasmids carrying *bla*_NDM-5_ (X3_N5_1).**

**Figure legend. A.** Schematic layout of IncX3 plasmid of group X3_N5_1 carrying *bla*_NDM-5_ identified in this study. Arrows represent the position and transcriptional direction of the open reading frames. Resistance genes are represented by red, genes for mobile elements by green, genes associated with conjugation in blue, replication-associated genes in pink, regulatory/accessory/hypothetical proteins in yellow. **B.** Colinear alignment of IncX3 plasmids harbouring *bla*_NDM-5_ of group X3_N5_1 (n=31). One plasmid from each clonal type was selected for the alignment. Alignment was performed by Mauve using DNASTAR (v17.1). Accession numbers of specific plasmids’ sequences are stated in Supplementary Table 18. The layout of genetic context has been outlined using Geneious (v11.0.2).

**Supplementary Figure 10.** **Characterization of** **IncFIB & IncHI1B plasmids carrying *bla*_NDM-1_ (FIB&HI1B_N1_1).**

**Figure legend. A.** Schematic layout of IncFIB & IncHI1B plasmid of group FIB&HI1B_N1_1 carrying *bla*_NDM-1_ identified in this study. Arrows represent the position and transcriptional direction of the open reading frames. Resistance genes are represented by red, genes for mobile elements by green, genes associated with conjugation in blue, replication-associated genes in pink, regulatory/accessory/hypothetical proteins in yellow. **B.** Colinear alignment of IncFIB & IncHI1B plasmids harbouring *bla*_NDM-1_ of group FIB&HI1B_N1_1 (n=6). One plasmid from each clonal type was selected for the alignment. Alignment was performed by Mauve using DNASTAR (v17.1). Accession numbers of specific plasmids’ sequences are stated in Supplementary Table 18. The layout of genetic context has been outlined using Geneious (v11.0.2).

**Supplementary Figure 11. Characterization of IncFIB(pQil) plasmids carrying *bla*_NDM-1_ (FIB(pQil)_N1_1).**

**Figure legend. A.** Schematic layout of IncFIB(pQil) plasmid of group FIB(pQil)_N1_1 carrying *bla*_NDM-1_ identified in this study. Arrows represent the position and transcriptional direction of the open reading frames. Resistance genes are represented by red, genes for mobile elements by green, genes associated with conjugation in blue, replication-associated genes in pink, regulatory/accessory/hypothetical proteins in yellow. **B.** Colinear alignment of IncFIB(pQil) plasmids harbouring *bla*_NDM-1_ of group FIB(pQil)_N1_1 (n=2). One plasmid from each clonal type was selected for the alignment. Alignment was performed by Mauve using DNASTAR (v17.1). Accession numbers of specific plasmids’ sequences are stated in Supplementary Table 18. The layout of genetic context has been outlined using Geneious (v11.0.2).

**Supplementary Figure 12.** **Characterization of** **IncFIA plasmids carrying *bla*_NDM-1_ (FIA_N1_1)**

**Figure legend. A.** Schematic layout of IncFIA plasmid of group FIA_N1_1 carrying *bla*_NDM-1_ identified in this study. Arrows represent the position and transcriptional direction of the open reading frames. Resistance genes are represented by red, genes for mobile elements by green, genes associated with conjugation in blue, replication-associated genes in pink, regulatory/accessory/hypothetical proteins in yellow. **B.** Colinear alignment of IncFIA plasmids harbouring *bla*_NDM-1_ of group FIA_N1_1 (n=2). One plasmid from each clonal type was selected for the alignment. Alignment was performed by Mauve using DNASTAR (v17.1). Accession numbers of specific plasmids’ sequences are stated in Supplementary Table 18. The layout of genetic context has been outlined using Geneious (v11.0.2).

**Supplementary Figure 13.** **Characterization of** **IncX3 plasmids carrying *bla*_NDM-7_ (X3_N7_1).**

**Figure legend. A.** Schematic layout of IncX3 plasmid of group X3_N7_1 carrying *bla*_NDM-7_ identified in this study. Arrows represent the position and transcriptional direction of the open reading frames. Resistance genes are represented by red, genes for mobile elements by green, genes associated with conjugation in blue, replication-associated genes in pink, regulatory/accessory/hypothetical proteins in yellow. **B.** Colinear alignment of IncX3 plasmids harbouring *bla*_NDM-7_ of group X3_N7_1 (n=6). One plasmid from each clonal type was selected for the alignment. Alignment was performed by Mauve using DNASTAR (v17.1). Accession numbers of specific plasmids’ sequences are stated in Supplementary Table 18. The layout of genetic context has been outlined using Geneious (v11.0.2).

**Supplementary Figure 14.** **Characterization of** **IncA/C2 plasmids carrying *bla*_OXA-181_ (A/C2_O181_1).**

**Figure legend. A.** Schematic layout of IncA/C2 plasmid of group A/C2_O181_1 carrying *bla*_OXA-181_ identified in this study. Arrows represent the position and transcriptional direction of the open reading frames. Resistance genes are represented by red, genes for mobile elements by green, genes associated with conjugation in blue, replication-associated genes in pink, regulatory/accessory/hypothetical proteins in yellow. **B.** Colinear alignment of IncA/C2 plasmids harbouring *bla*_OXA-181_ of group A/C2_O181_1 (n=2). One plasmid from each clonal type was selected for the alignment. Alignment was performed by Mauve using DNASTAR (v17.1). Accession numbers of specific plasmids’ sequences are stated in Supplementary Table 18. The layout of genetic context has been outlined using Geneious (v11.0.2).

**Titles of Supplementary Tables**

**Supplementary Table 1. The recent update of AMR surveillance in the countries of SA according to GLASS Early Implementation Report 2020.**

**Supplementary Table 2. Scoping findings relevant to this study from previous literature searches.**

**Supplementary Table 3. Total number of culture-positive clinical specimens from different wards of DMCH in this study (n=1893*).**

**Supplementary Table 4. The frequency of isolation of CRE and CSE from different clinical samples.**

**Supplementary Table 5. Total number of clinical isolates identified in this study (n=1583).**

**Supplementary Table 6. The associations of prevalent ARGs with** ***bla*_NDM-5_-positive isolates compared to *bla*_NDM-5_-negative isolates.**

**Supplementary table 7.** **The associations of prevalent ARGs with *bla*_NDM-1_-positive isolates compared to *bla*_NDM-1_-negative isolates.**

**Supplementary Table 8.** **The associations of prevalent ARGs with *bla*_OXA-181_-positive isolates compared to *bla*_OXA-181_-negative isolates.**

**Supplementary Table 9. Range of carbapenems’ MIC of OXA-232 and OXA-181 producing Enterobacterales.**

**Supplementary Table 10. The frequency of patients with at least one effective antimicrobial stratified based on patients’ outcome.**

**Supplementary Table 11. Distribution of carbapenem-resistant *E. coli* among major clonal types compared to carbapenem-sensitive *E. coli*.**

**Supplementary Table 12. Distribution of *E. coli* among different phylogroups and their corresponding STs (n=226).**

**Supplementary Table 13. The overall information obtained from time-scaled tress in this study.**

**Supplementary Table 14.** **Distribution of carbapenem resistant *K. pneumoniae* among major clonal types compared to CSE.**

**Supplementary Table 15. The prevalence of different capsular K loci among *K. pneumoniae* isolated in this study along with corresponding STs.**

**Supplementary Table 16. Brief description of microbiology capacity of the major MCHs of Bangladesh (Scoping exercise done by Timothy R. Walsh and Refath Farzana on May 2019).**

**Supplementary Table 17. Genome attributes of the isolates retrieved from NCBI.**
