# Supplementary figures and images for: Genomic Insights Into the Mechanism of Carbapenem Resistance Dissemination in Enterobacterales From a Tertiary Public Heath Setting in South Asia
Source: Clin Infect Dis. 2022 Apr 27;76(1):119–33. doi: 10.1093/cid/ciac287 (PMC9825829; doi:10.1093/cid/ciac287)

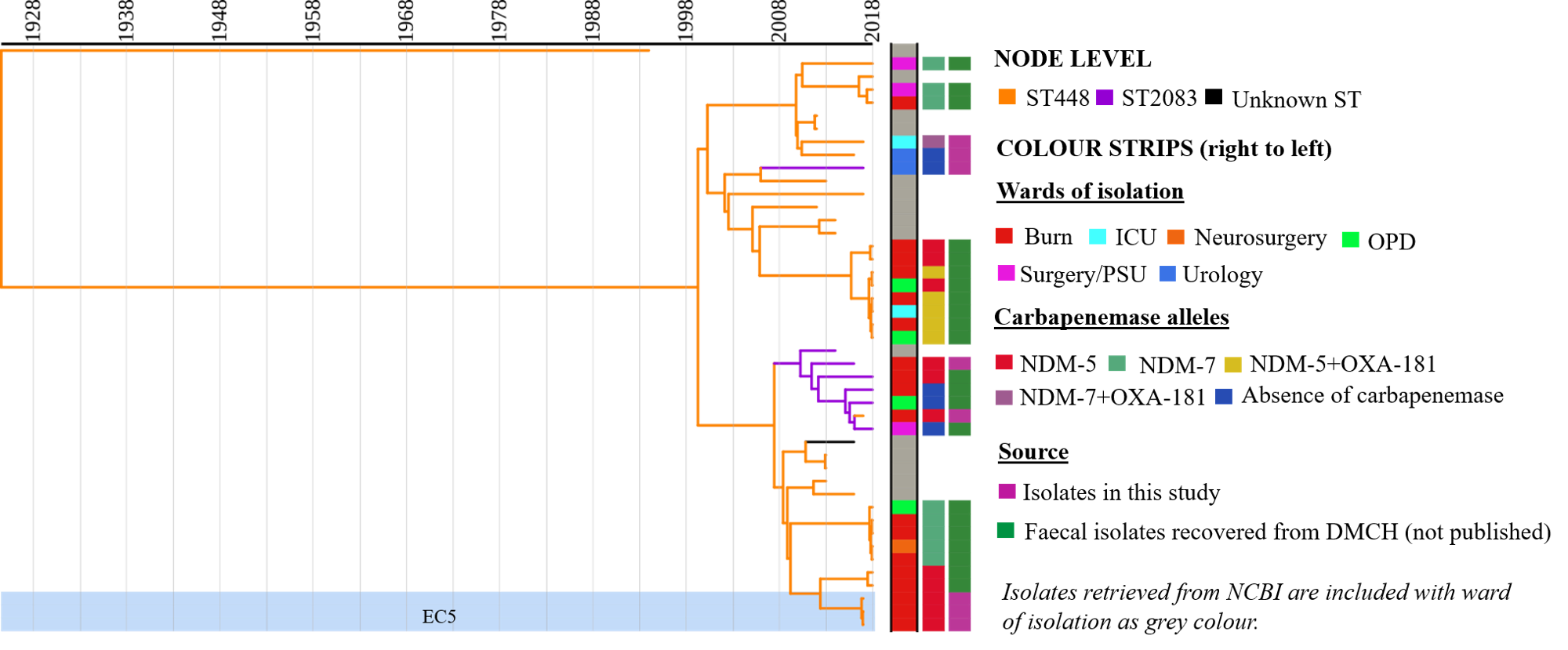

Supplement: ciac287_Supplementary_Data [file ciac287_supplementary_data.zip › Supplementary Figure 1.tif]

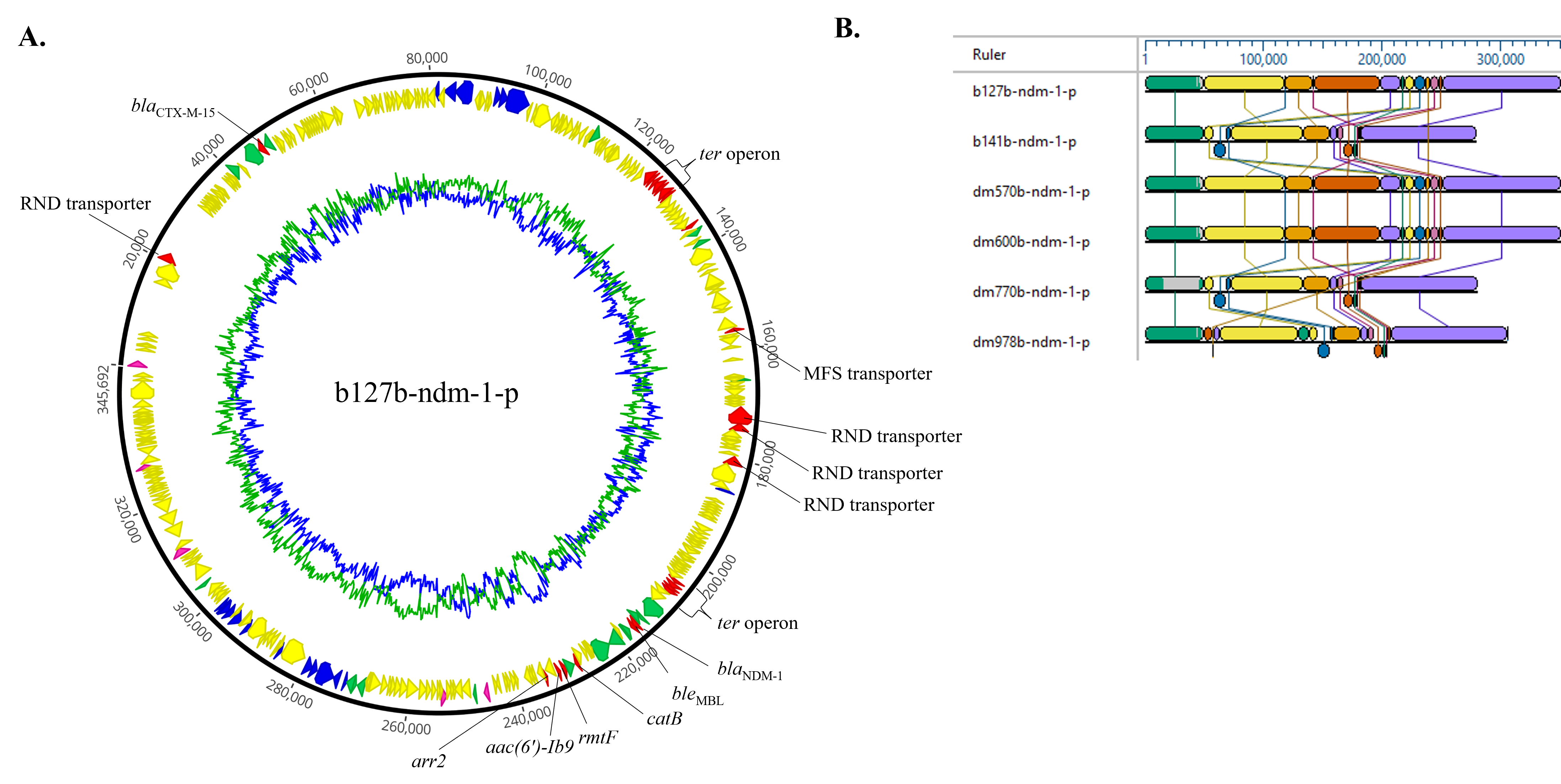

Supplement: ciac287_Supplementary_Data [file ciac287_supplementary_data.zip › Supplementary Figure 10.tif]

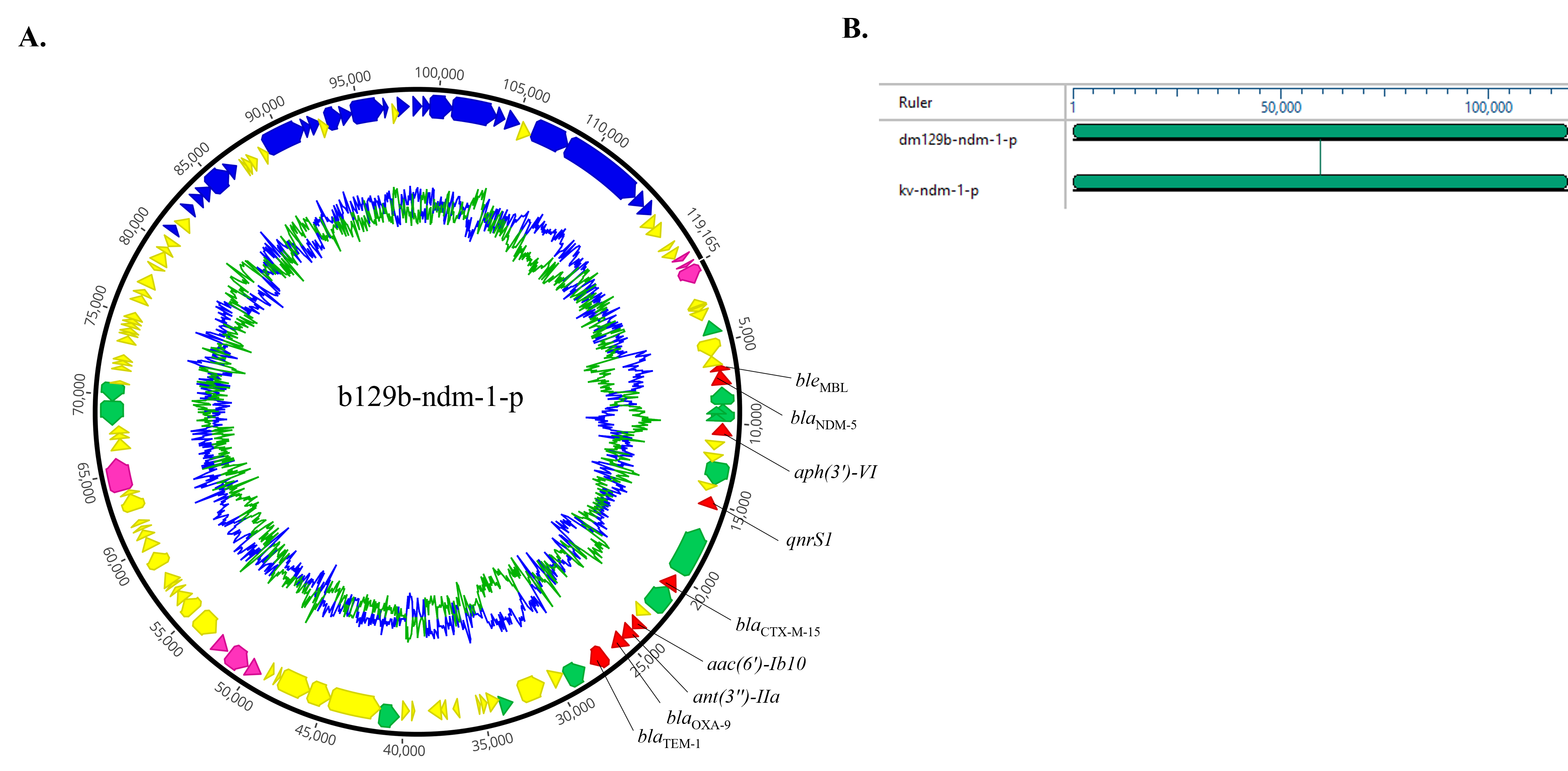

Supplement: ciac287_Supplementary_Data [file ciac287_supplementary_data.zip › Supplementary Figure 11.tif]

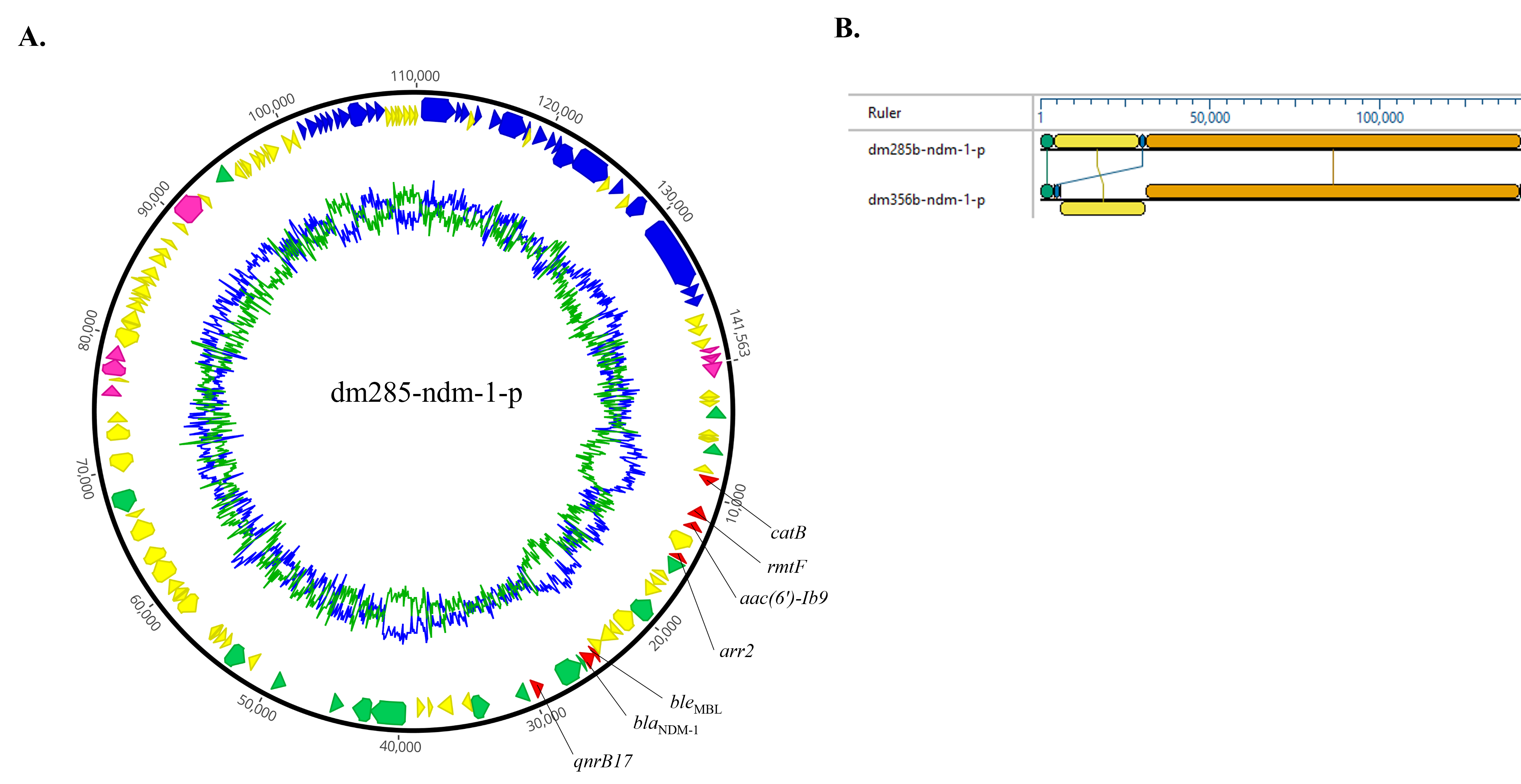

Supplement: ciac287_Supplementary_Data [file ciac287_supplementary_data.zip › Supplementary Figure 12.tif]

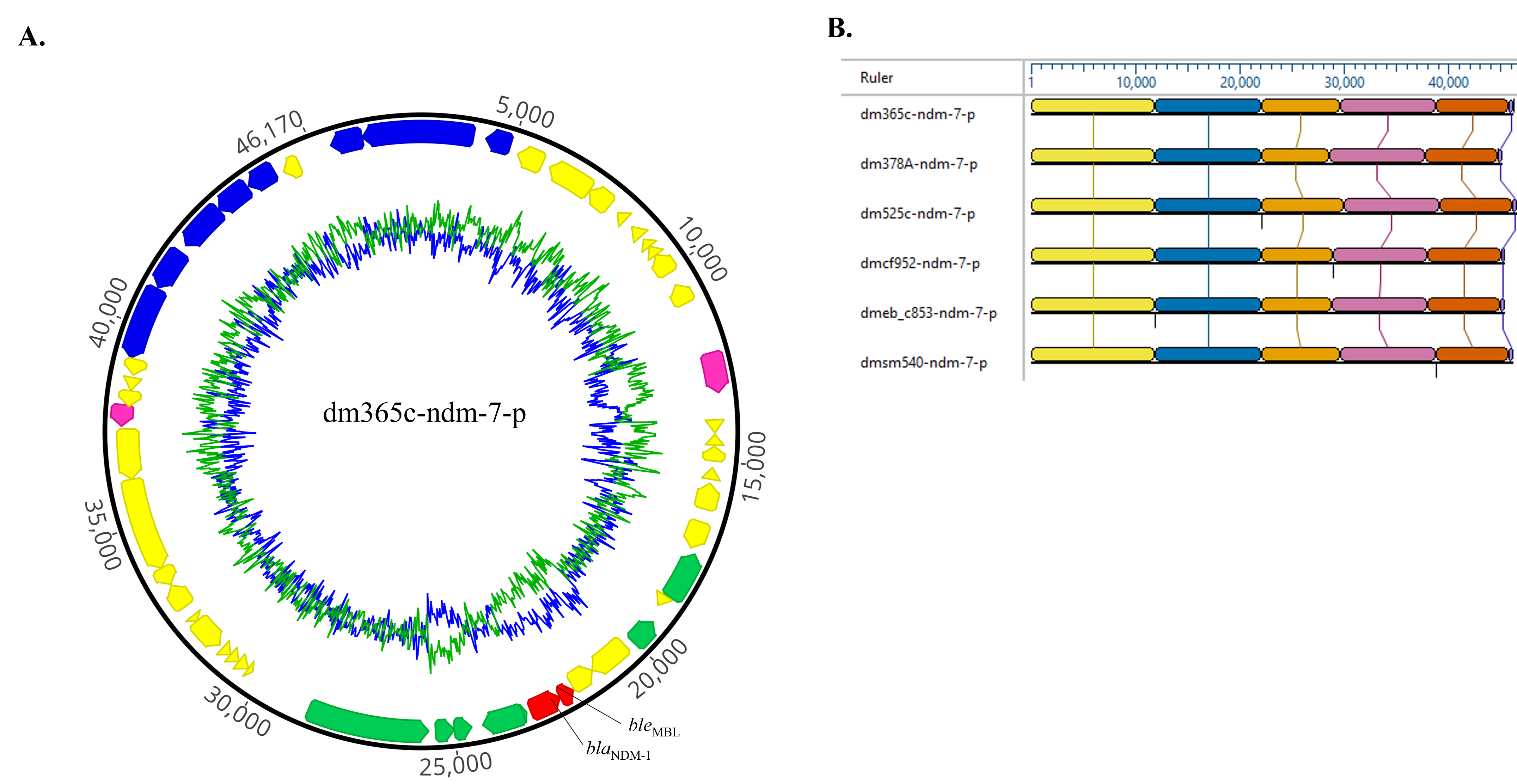

Supplement: ciac287_Supplementary_Data [file ciac287_supplementary_data.zip › Supplementary Figure 13.tif]

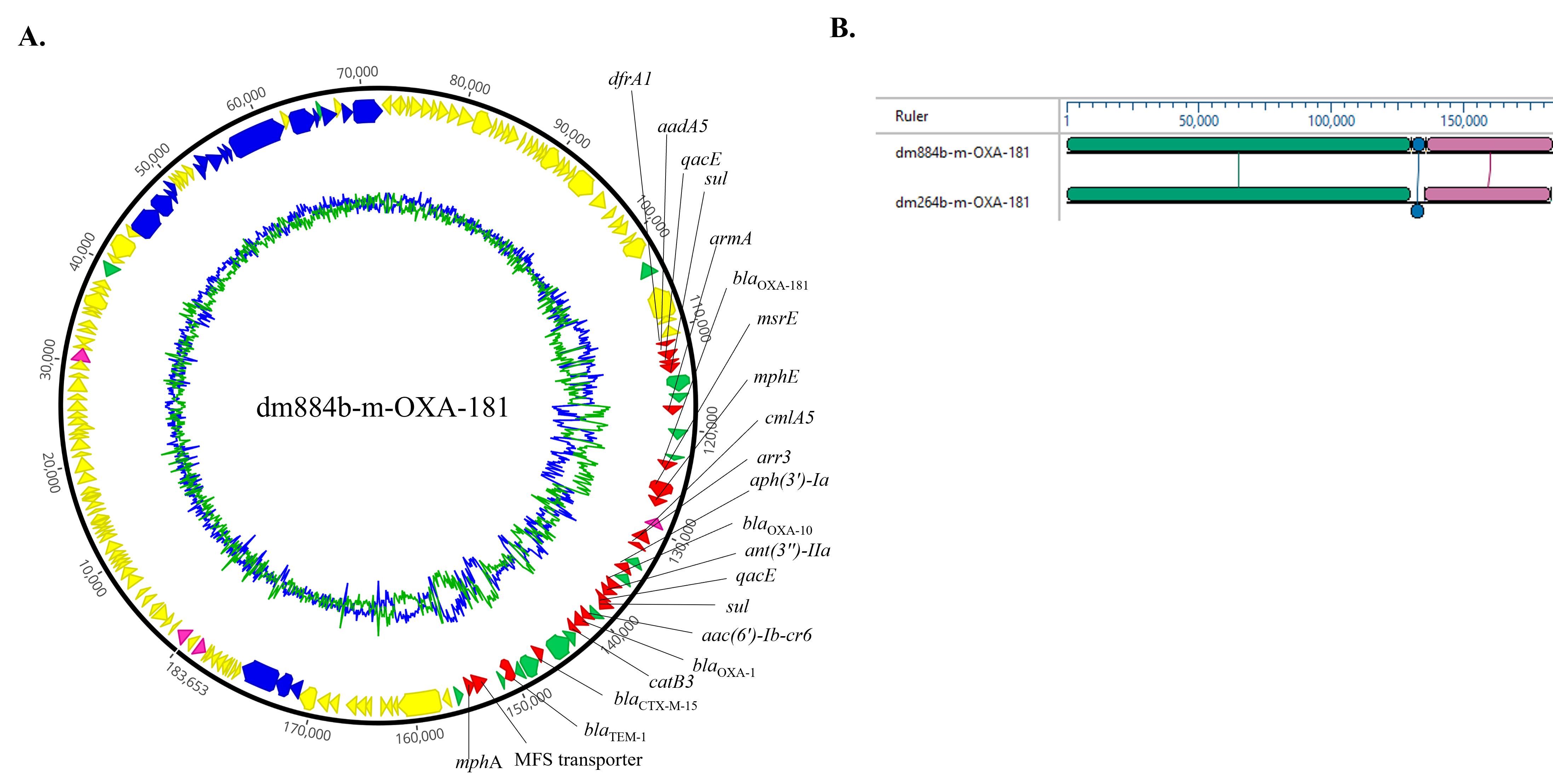

Supplement: ciac287_Supplementary_Data [file ciac287_supplementary_data.zip › Supplementary Figure 14.tif]

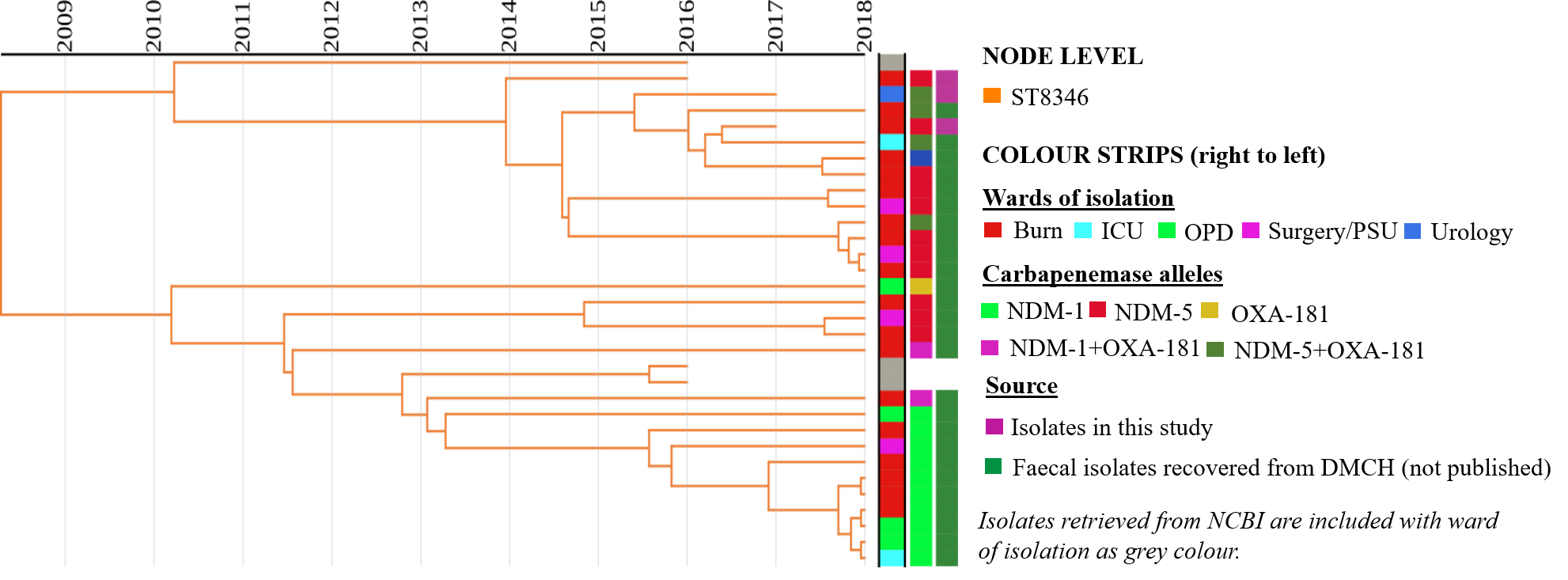

Supplement: ciac287_Supplementary_Data [file ciac287_supplementary_data.zip › Supplementary Figure 2.tif]

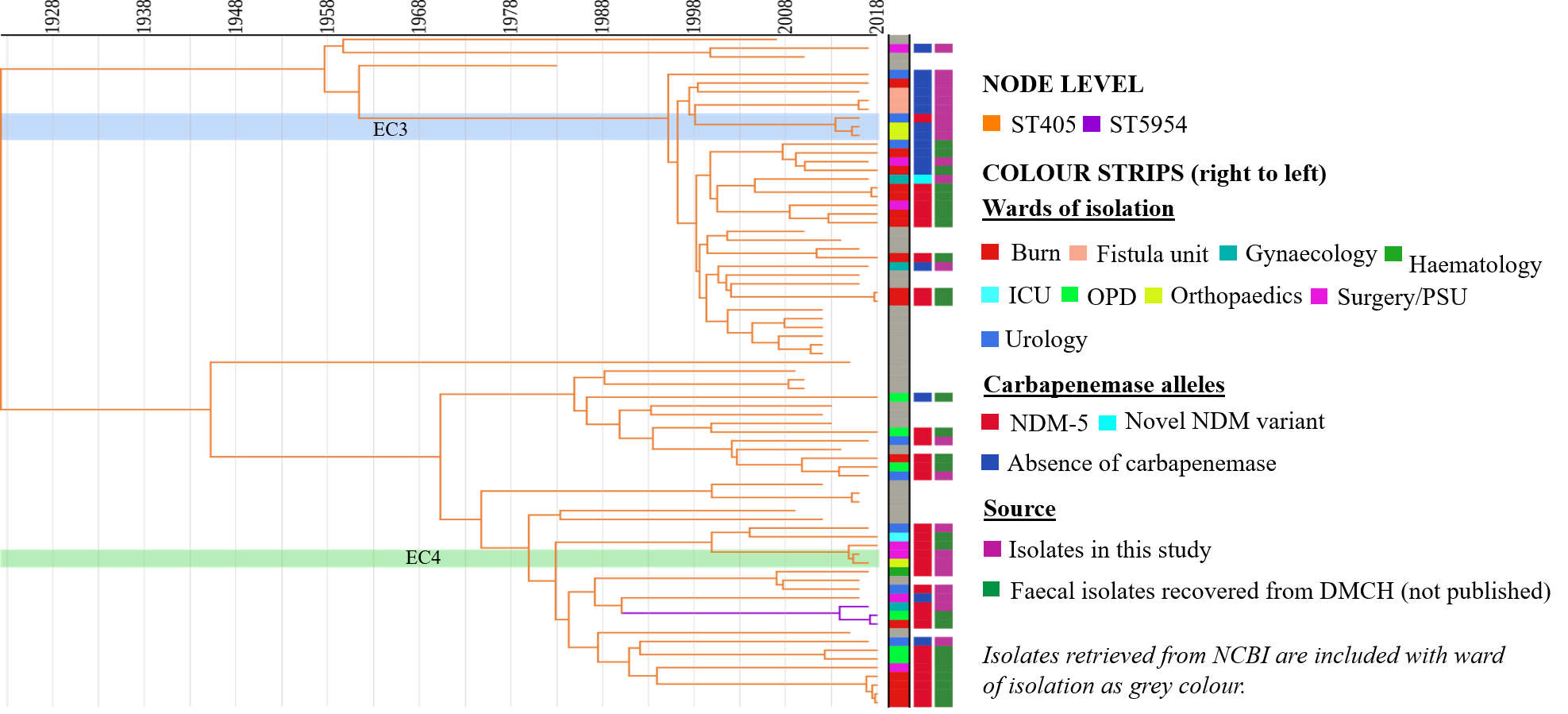

Supplement: ciac287_Supplementary_Data [file ciac287_supplementary_data.zip › Supplementary Figure 3.tif]

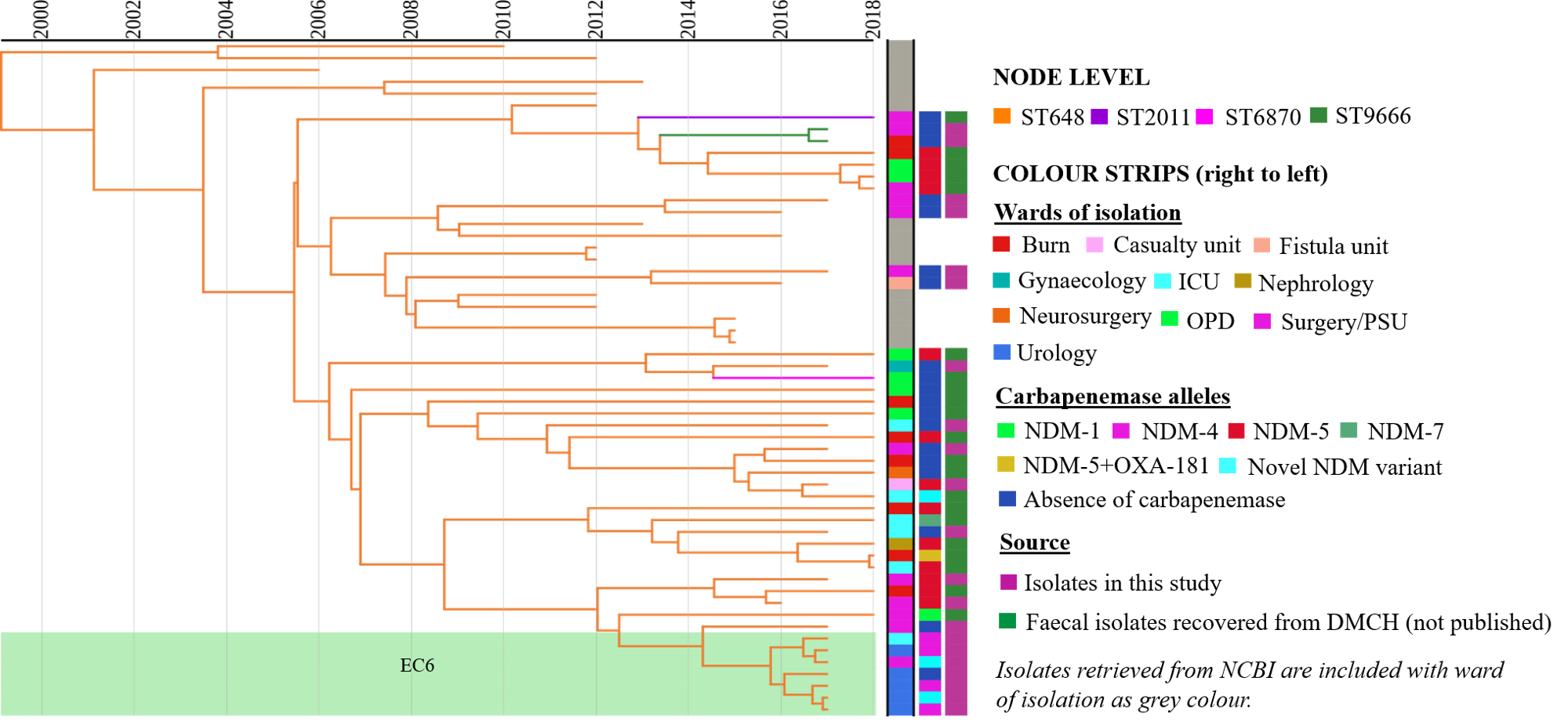

Supplement: ciac287_Supplementary_Data [file ciac287_supplementary_data.zip › Supplementary Figure 4.tif]

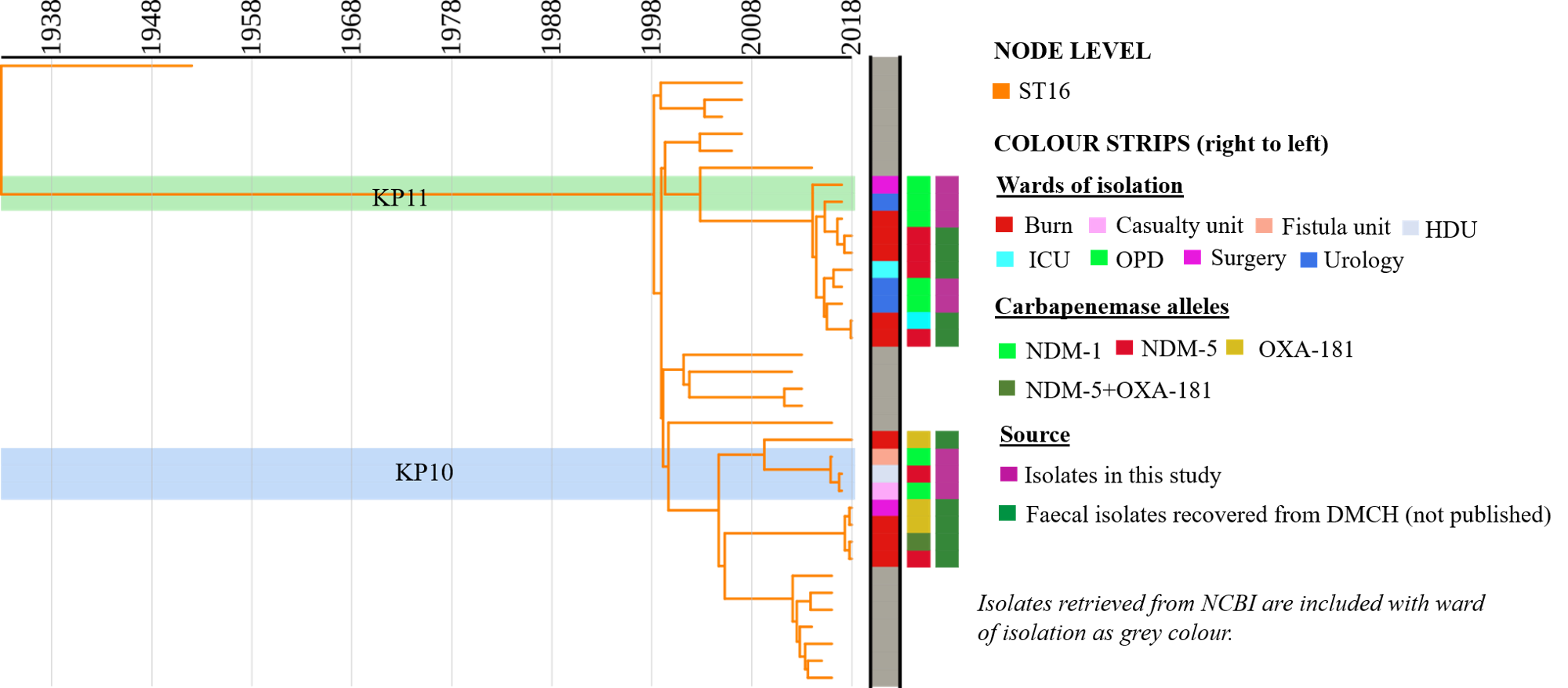

Supplement: ciac287_Supplementary_Data [file ciac287_supplementary_data.zip › Supplementary Figure 5.tif]

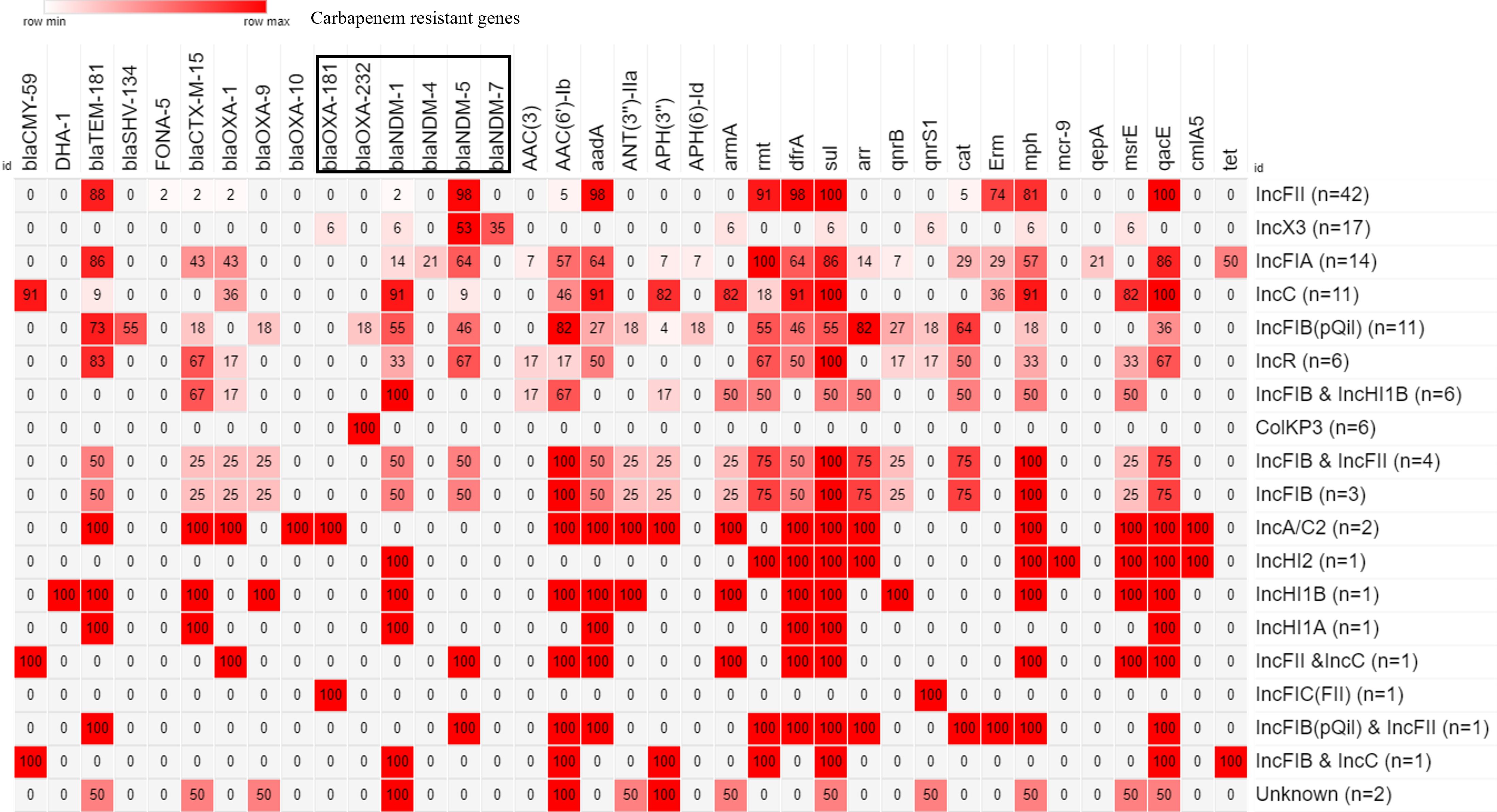

Supplement: ciac287_Supplementary_Data [file ciac287_supplementary_data.zip › Supplementary Figure 6.tif]

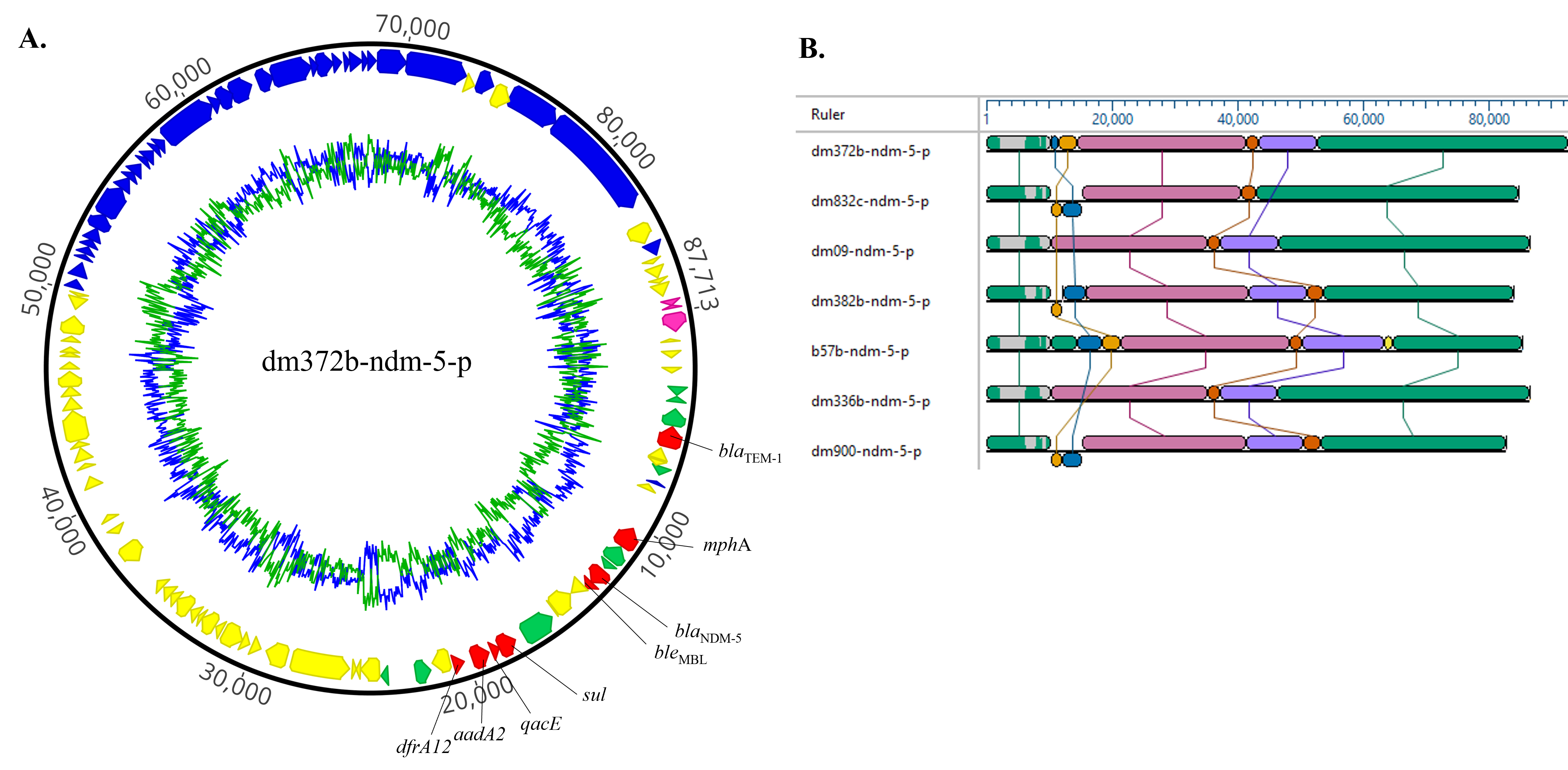

Supplement: ciac287_Supplementary_Data [file ciac287_supplementary_data.zip › Supplementary Figure 7.tif]

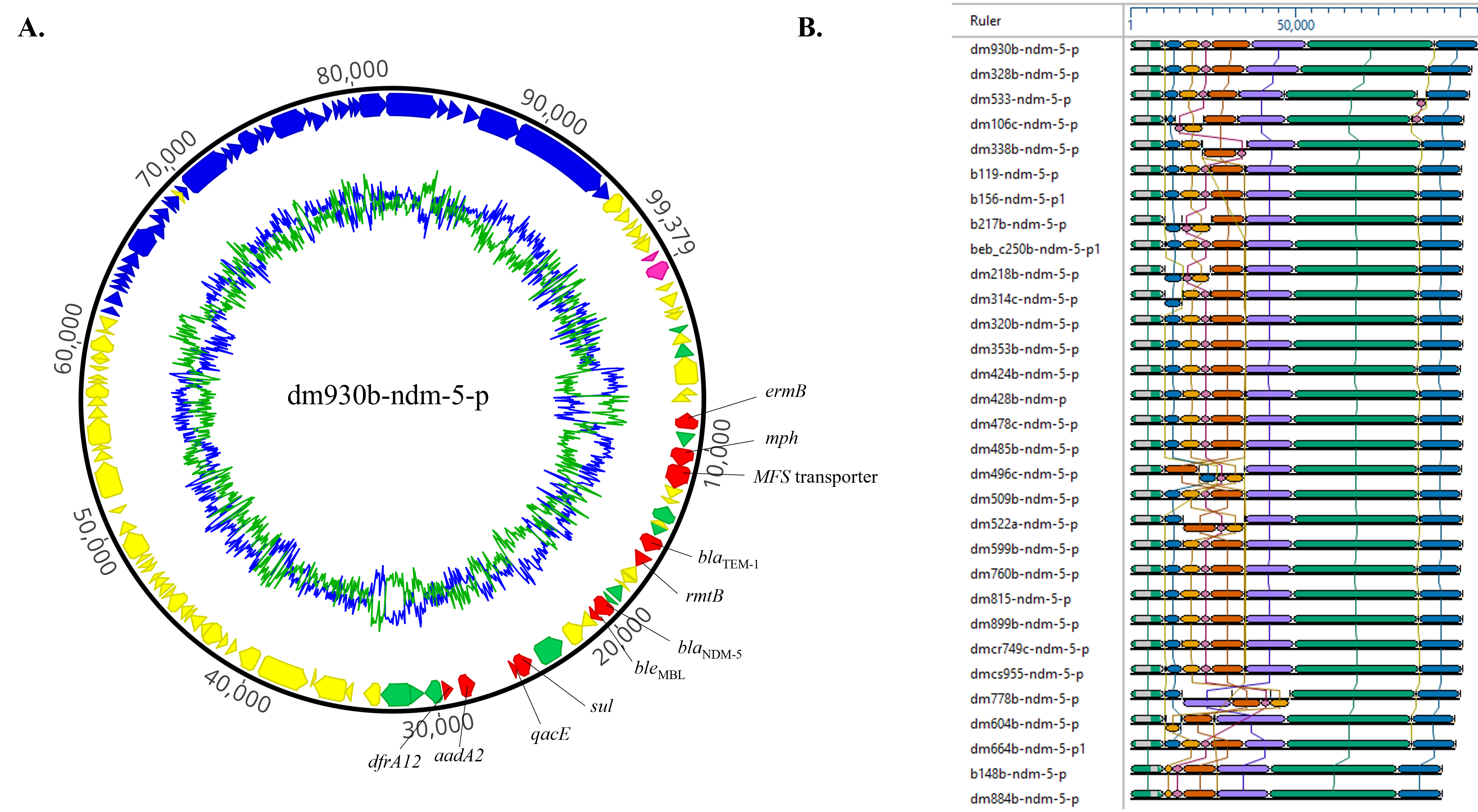

Supplement: ciac287_Supplementary_Data [file ciac287_supplementary_data.zip › Supplementary Figure 8.tif]

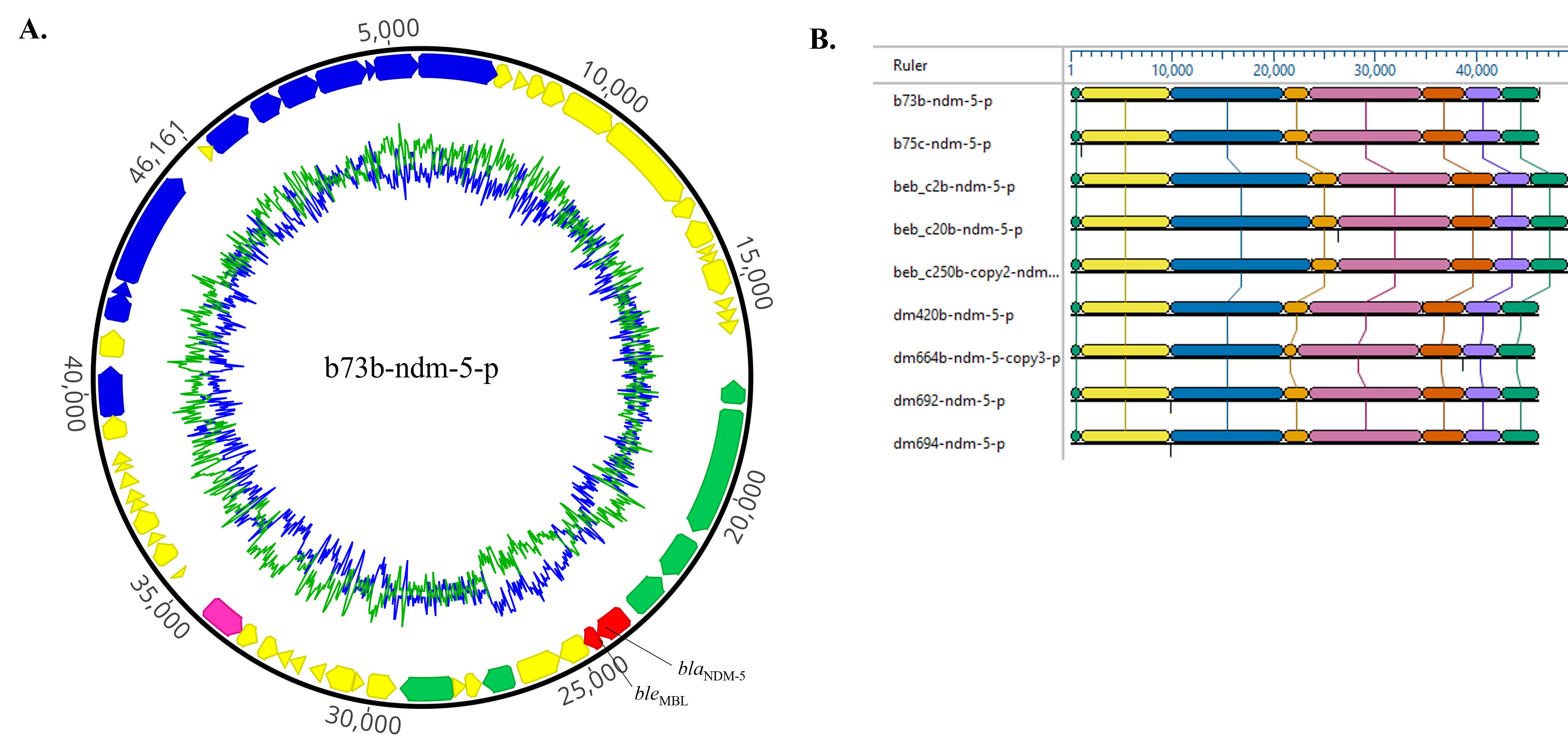

Supplement: ciac287_Supplementary_Data [file ciac287_supplementary_data.zip › Supplementary Figure 9.tif]
